# Transition-metal-free decarboxylative bromination of aromatic carboxylic acids†
†Electronic supplementary information (ESI) available. See DOI: 10.1039/c8sc01016a


**DOI:** 10.1039/c8sc01016a

**Published:** 2018-03-26

**Authors:** Jacob M. Quibell, Gregory J. P. Perry, Diego M. Cannas, Igor Larrosa

**Affiliations:** a School of Chemistry , University of Manchester , Oxford Road , Manchester , M13 9PL , UK . Email: igor.larrosa@manchester.ac.uk; b Institute of Transformative Bio-Molecules (WPI-ITbM) and Graduate School of Science , Nagoya University , Chikusa , Nagoya 464-8602 , Japan

## Abstract

Methods for the conversion of aliphatic acids to alkyl halides have progressed significantly over the past century, however, the analogous decarboxylative bromination of aromatic acids has remained a longstanding challenge. The development of efficient methods for the synthesis of aryl bromides is of great importance as they are versatile reagents in synthesis and are present in many functional molecules. Herein we report a transition metal-free decarboxylative bromination of aromatic acids. The reaction is applicable to many electron-rich aromatic and heteroaromatic acids which have previously proved poor substrates for Hunsdiecker-type reactions. In addition, our preliminary mechanistic study suggests that radical intermediates are not involved in this reaction, which is in contrast to classical Hunsdiecker-type reactivity. Overall, the process demonstrates a useful method for producing valuable reagents from inexpensive and abundant starting materials.

## Abstract 2

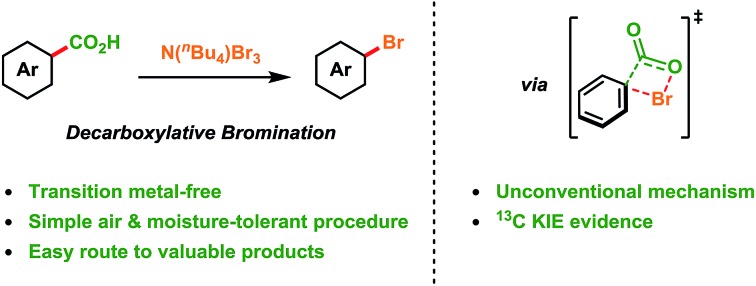
Aromatic acids are converted into aryl bromides simply and efficiently *via* decarboxylation providing new depth and insight into Hunsdiecker-type reactivity.

## Introduction

**Scheme 1:**
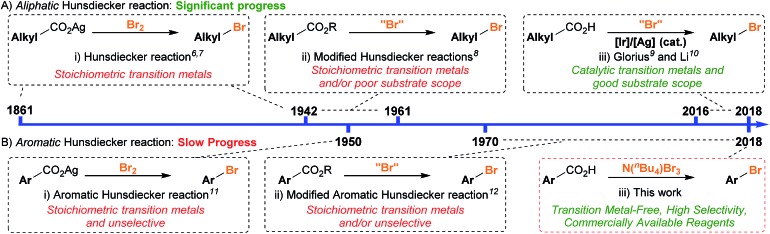
Timeline of the contrasting progress in (A) aliphatic *vs.* (B) aromatic decarboxylative bromination.

Aryl bromides are the substrate of choice when performing transition metal-catalysed cross-coupling reactions^1^ or preparing Grignard and organolithium reagents.^2^ They are also used in a variety of other transformations, such as nucleophilic substitution and HalEx reactions,^3^ and are the core structures in many natural products and dyes.^4^ Consequently, developing efficient methods for the synthesis of aryl bromides remains an important objective.^5^ The ability to directly substitute a carboxyl group with a bromo group has interested the synthetic community for many years. This transformation was first demonstrated by Borodine over a century ago, but came to bear the name “The Hunsdiecker Reaction” during the 1940's and is now a fundamental reaction in organic synthesis.^6^,^7^ The reaction involves the mixing of an aliphatic carboxylic acid with bromine in the presence of a silver salt to produce the desired alkyl halide. Various developments in this area were made during the latter half of the 20^th^ century,^8^ however, the applicability of all these methods was limited due to the requirement of stoichiometric transition metal salts and/or poor generality. It is only recently that significant progress has been made in the decarboxylative bromination of aliphatic acids (Scheme 1A). Namely, the groups of Glorius^9^ and Li^10^ have demonstrated that primary, secondary and tertiary carboxylic acids can be converted into the corresponding alkyl bromides in the presence of either an iridium or silver catalyst.

**Scheme 2:**
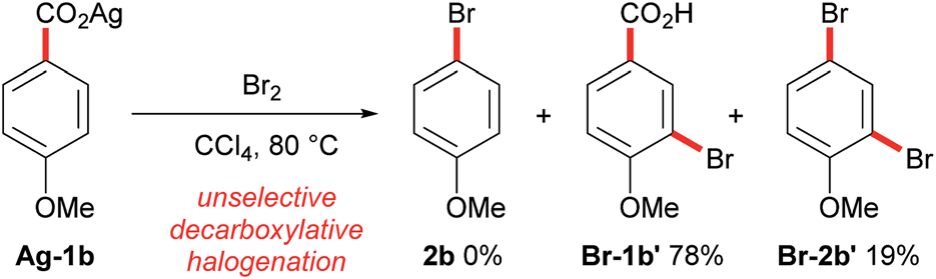
Current status of the aromatic Hunsdiecker reaction.

Conversely, the decarboxylative bromination of aromatic acids has suffered from a slower pace of development (Scheme 1B). Early findings demonstrated that the bromination of aromatic acids could indeed proceed, however, the reaction with electron-rich substrates was unselective and electron-deficient aromatics gave varying yields (Scheme 2).^11^ Many have looked to solve these issues, but current procedures still suffer as they (a) require stoichiometric transition metals, (b) are of poor generality, and/or (c) are unselective.^12^ This is disappointing as decarboxylative bromination holds potential as an economic route for aryl halide formation;^13^ benzoic acids are inexpensive and abundant, and the bromide group is a handle for selective transformations. We were eager to reinvestigate the aromatic Hunsdiecker reaction in order to establish a more efficient and selective route for aryl bromide formation. Herein we reveal the development of a transition metal-free decarboxylative bromination that is applicable to a variety of electron-rich aromatic acids. In addition, preliminary investigations begin to shed light on the mechanism of this reaction.

## Results and discussion

We have recently reported a transition-metal-free decarboxylative iodination of aromatic acids.^14^ The success of this procedure lies in the ability to prepare a variety of aryl iodides, simply by heating a benzoic acid in the presence of I_2_. We therefore questioned whether a similarly efficient and low-cost decarboxylative bromination could be developed. We began our investigation by exposing the benzoic acid **1a** to our previous conditions, but switching the halogen source from I_2_ to Br_2_ (Table 1). Unfortunately, this resulted in the undesired formation of the brominated acid **1a′** and dibrominated product **2a′** and none of the desired product **2a** (Table 1, entry 1). This reactivity is comparable with the aromatic Hunsdiecker reaction shown in Scheme 2 and demonstrates the challenges for achieving a selective bromination. By lowering the equivalents of Br_2_ the selectivity could be improved, however, a large amount of the brominated acid **2a′** was still produced (entry 2). We then investigated less electrophilic bromine sources, such as NBS (*N*-bromosuccinimide) and DBH (1,3-dibromo-5,5-dimethylhydantoin), however a mixture of products was still obtained and the desired product was formed in low yield (entries 3 and 4). We then turned to the use of tribromide reagents as bromine sources for this transformation. Although pyridinium tribromide performed poorly in this reaction (entry 5), we found that tetraalkyl ammonium tribromide salts, N(Me_4_)Br_3_ and N(^*n*^Bu_4_)Br_3_, displayed good reactivity and high selectivity for the desired decarboxylative bromination (entries 6 and 7). N(^*n*^Bu_4_)Br_3_ was chosen as the brominating reagent of choice, allowing the product to be isolated in 90% yield. Further control experiments revealed that the reaction does not proceed in the absence of a base (entry 8), but that performing the reaction in the dark or adding one equivalent of water had little effect on the reaction (entries 9 and 10).^15^

**Table 1:** Optimisation of the transition metal free decarboxylative bromination^*a*^

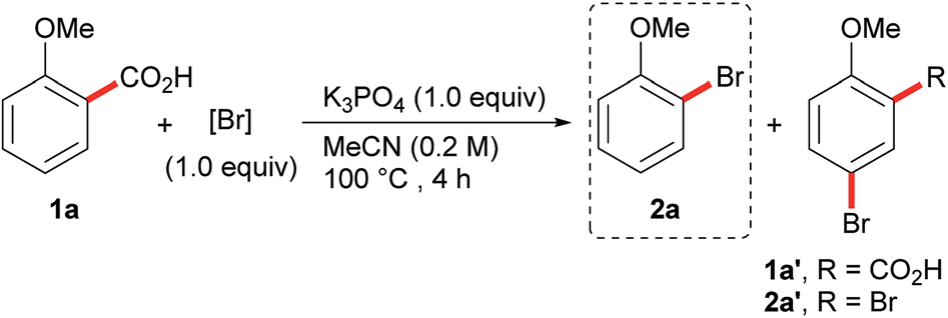					
Entry	[Br]	**1a**	**2a**	**1a′**	**2a′**
1^*b*^	Br_2_	Trace	0	23	72
2	Br_2_	8	26	56	3
3	NBS^*c*^	58	21	9	Trace
4	DBH^*d*^	6	40	32	7
5	PyHBr_3_^*e*^	39	6	54	0
6	N(Me_4_)Br_3_	6	85	5	2
7	N(^*n*^Bu_4_)Br_3_	5	91 (90)^*f*^	1	Trace
8^*g*^	N(^*n*^Bu_4_)Br_3_	100	0	0	0
9^*h*^	N(^*n*^Bu_4_)Br_3_	11	84	2	Trace
10^*i*^	N(^*n*^Bu_4_)Br_3_	10	85	Trace	3

^*a*^Reaction conditions: **1a** (0.2 mmol), [Br] (0.2 mmol, 1.0 equiv.), K_3_PO_4_ (0.2 mmol, 1.0 equiv.), MeCN (1.0 mL), 100 °C, 4 h.

^*b*^Br_2_ (0.6 mmol, 3.0 equiv.).

^*c*^ *N*-bromosuccinimide.

^*d*^1,3-Dibromo-5,5-dimethylhydantoin.

^*e*^Pyridinium tribromide.

^*f*^Yield in parenthesis is of isolated material. Isolated as a mixture with **2a′** (**2a** : **2a′**, >150 : 1).

^*g*^No K_3_PO_4_ added.

^*h*^Performed in the dark.

^*i*^1.0 equiv. H_2_O added.

**Scheme 3:**
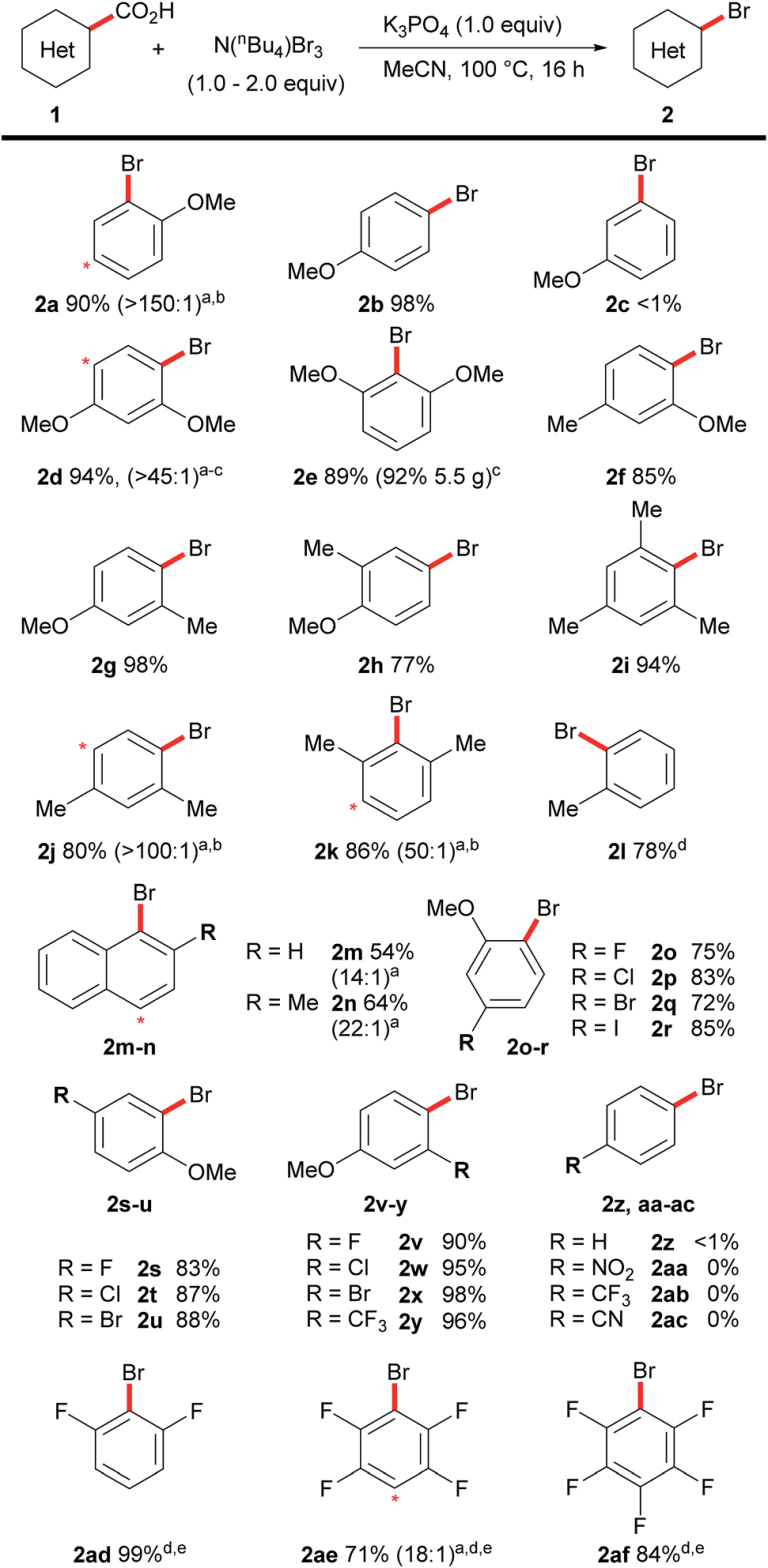
Scope of the decarboxylative bromination of aromatic acids. Reactions carried out on a 0.5 mmol scale. ^a^Ratios in brackets indicate mono : dibrominated material by NMR analysis before separation. Asterisk indicates position of dibromination. ^b^Isolated as mixture. ^c^Room temperature. ^d^Yields determined by NMR analysis. ^e^N(^*n*^Bu_4_)Br_3_ (4.0 equiv.).

**Scheme 4:**
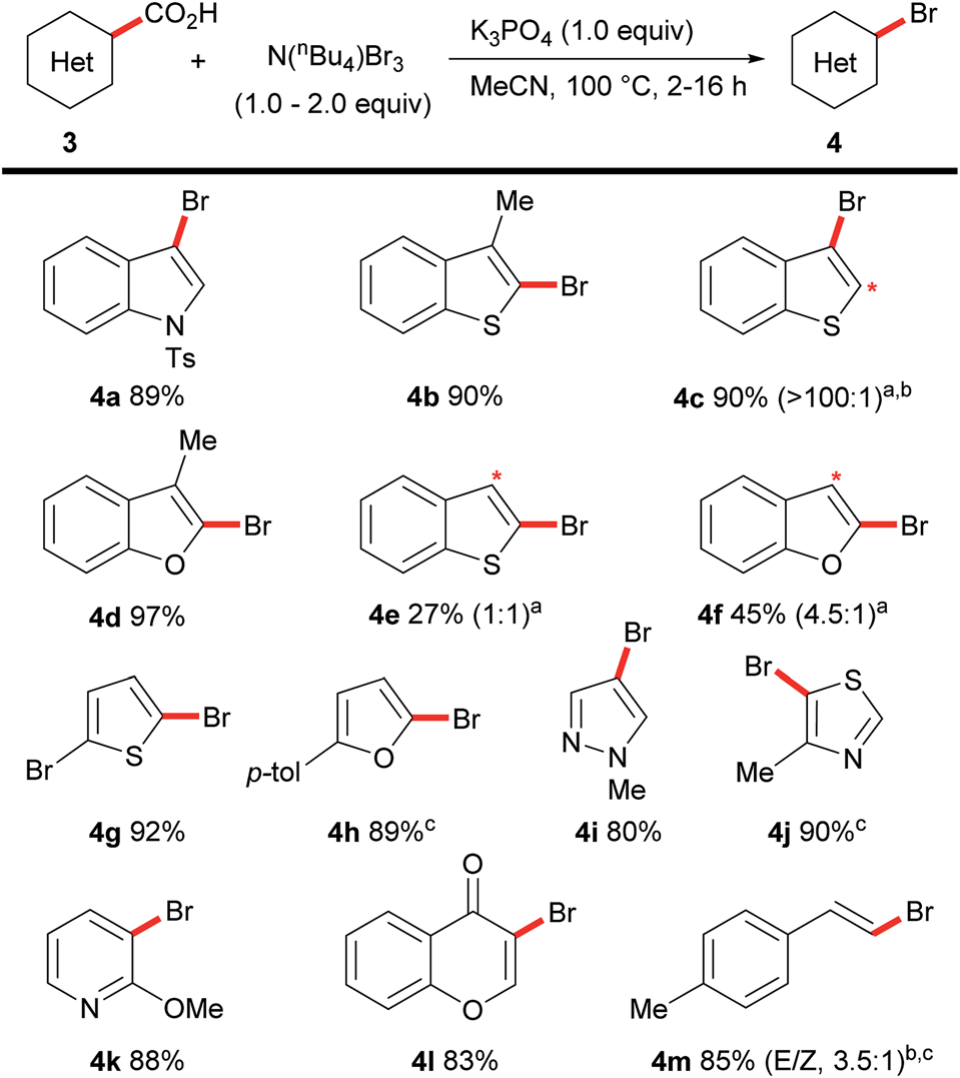
Scope of the decarboxylative bromination of heteroaromatic acids. Reactions carried out on a 0.5 mmol scale ^a^Ratios in brackets indicate mono : dibrominated material by NMR analysis before separation. Asterisk indicates position of dibromination. ^b^Isolated as mixture. ^c^50 °C.

Having demonstrated an efficient transition metal-free decarboxylative bromination, we then turned to exploring the scope of this reaction (Scheme 3). Previously, the decarboxylative bromination of 4-methoxybenzoic acid under Hunsdiecker-type conditions resulted in a mixture of products (Scheme 2), therefore we were impressed to observe the formation of the aryl bromide **2b** in high yield and high selectivity. This clearly demonstrates the advantages of our procedure over previous techniques. The desired product, **2c**, was not observed when using 3-methoxybenzoic acid, suggesting the position of decarboxylation must be sufficiently nucleophilic for the decarboxylative bromination to occur. Other highly electron-rich substrates (**1d–h**) could also undergo the desired transformation, including non-*ortho*-substituted benzoic acids (**1b**, **1h**), which are generally unreactive in transition metal-mediated decarboxylative functionalisations. The procedure can also be applied on a large scale, thus, 5.5 g of the brominated product **2e** was prepared using the standard conditions on the bench top at room temperature, without requiring column chromatography. Polymethylated benzoic acids are poorly reactive substrates in transition metal-mediated decarboxylations, but they, and even simple toluic acid, showed good reactivity under our conditions (**2i–2l**). The procedure could also be applied to napthoic acids, despite a slight loss in selectivity (**2m**, **2n**). The position of dibromination is indicated in both Schemes 3 and 4 by a red asterisk. A range of halogenated and trifluoromethylated benzoic acids were successfully decarboxylated, however, the presence of a methoxy group was necessary to maintain efficient reactivity (**2o–2y**). Benzoic acids that do not bear electron-donating substituents were unreactive under these conditions (**2z–2ac**). In light of this, we were surprised to observe good reactivity with electron-poor polyfluorinated benzoic acids (**2ad–2af**). This goes against the general trend of reactivity in this reaction and we are currently investigating the cause of this unique behaviour.

The procedure could also be applied to a range of heteroaromatic acids (Scheme 4). The bromination of heteroaromatic acids is highly limited as elevated temperatures (>160 °C) and stoichiometric transition metals are generally necessary.^16^ Our conditions were applicable to indoles (**4a**), benzothiophenes (**4b**, **4c**) and benzofurans (**4d**). Unfortunately, under standard reaction conditions benzothiophene-(**4e**) and, to an extent, benzofuran-2-carboxylic acid (**4f**) afforded undesirable levels of dihalogenation. This side-reaction, namely the β-bromination of benzo-fused five-membered heterocycles, is well-known to proceed readily at room-temperature with a variety of brominating agents; thus representing a limitation of our methodology. A range of 5-membered heterocycles (**4g–4j**) as well as pyridine (**4k**), chromone (**4l**) and cinnamic acid (**4m**) derivatives all underwent the desired decarboxylative bromination selectively.

**Fig. 1:**
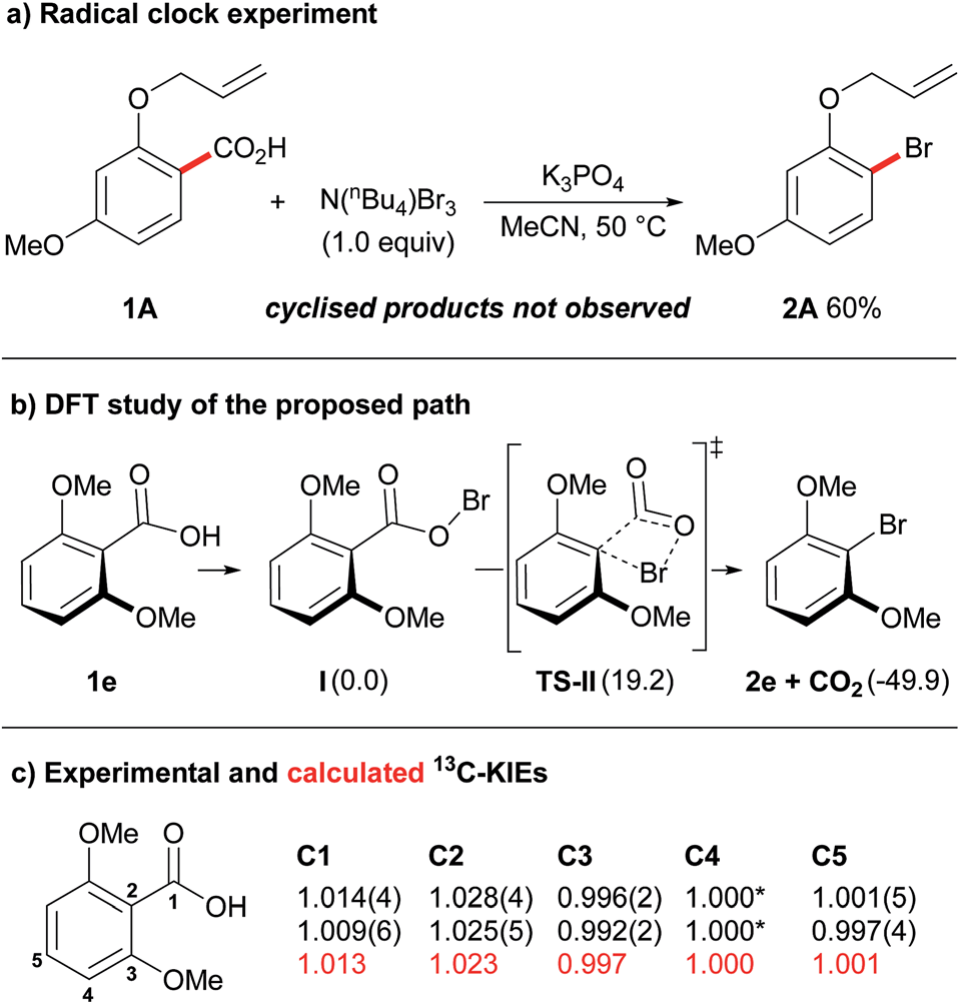
Mechanistic investigations. (a) Standard reaction conditions with benzoic acid **1A** at 50 °C for 30 min. (b) Energies calculated in acetonitrile (B3LYP-D3BJ/6-31+G(d)).^24^ Gibbs free energies (*G*) in kcal mol^–1^. (c) Experimental KIEs (in black), the uncertainty on the last figure is reported in brackets. For C4 a KIE of 1.000 was assumed.^25^ Calculated KIEs (in red) for the proposed path.^26^

Having established an efficient protocol for the decarboxylative bromination of aromatic acids, we began a preliminary mechanistic investigation. Our initial experiment involved the use of the oxyallyl-substituted benzoic acid **1A** as a radical clock (Fig. 1a). The formation of cyclised products, *via* attack of an aryl radical on the pendent allyl chain of this compound, is an extremely fast process (*k* = 8 × 10^9^ s^–1^),^17^ therefore, if cyclised products were to be observed in this reaction a radical mechanism could be suggested. Upon exposing oxyallyl-substituted benzoic acid **1A** to our standard reaction conditions we only observed the formation of the aryl bromide **2A** and none of the cyclised product. In light of this, we can suggest that either the reaction does not proceed through a radical mechanism or, if radicals are involved, then the rate at which the product is formed from the radical intermediate is an exceptionally fast process. This is an interesting observation as similar experiments that have previously been conducted on Hunsdiecker-type decarboxylations of aliphatic carboxylic acids have strongly supported a radical mechanism.^18^ Likewise, previous Hunsdiecker-type decarboxylations of aromatic acids have also been proposed to proceed through radical intermediates.12k–l,15b,^18^ Overall, although the above result does not definitively rule out a radical mechanism, it calls for a more thorough evaluation of Hunsdiecker-type reactivity.

Our previous work on decarboxylative iodinations established a concerted decarboxylation-iodination process, *via* a 4-membered transition state, as a possible non-radical pathway for decarboxylative halogenations.^14^ Following a similar protocol (Fig. 1b), our DFT study found a pathway for decarboxylative bromination proceeding through an analogous concerted decarboxylation-bromination transition state, **TS-II**. Thus, our current mechanistic hypothesis is as follows: the benzoic acid is initially transformed into the hypobromite species **I** upon exposure to K_3_PO_4_ and N(^*n*^Bu_4_)Br_3_.^19^ The hypobromite then undergoes decarboxylation *via* a 4-membered transition state (**TS-II**), to provide the product **2e** with concomitant loss of CO_2_. The barrier for this transformation was calculated to be 19.2 kcal mol^–1^, which is consistent with a process that proceeds at room temperature.^20^

We further probed the mechanism of this reaction by conducting ^13^C/^12^C KIE experiments. Heavy atom isotope effects have been widely used as a means to study decarboxylation events in chemical and enzymatic processes.^21^ The KIE at the C1 position for decarboxylation processes can be accurately determined at natural abundance by measuring the isotopic composition of the evolved CO_2_ through mass-spectroscopy technique.^22^ Unfortunately, with this method no information is gained on the other carbon atoms. Exploiting quantitative ^13^C-NMR, Singleton and co-workers have devised a useful procedure for determining intermolecular competitive ^13^C/^12^C KIEs at all positions at natural abundance.^23^ Over the course of the reaction, the starting material is progressively enriched in the slowest reacting isotopologues. By evaluating the isotopic composition in the starting material before and after the reaction the KIEs can be determined. This has proved a powerful tool in elucidating reaction mechanisms and we were eager to test its value on our system. Two independent experiments were performed on 2,6-dimethoxybenzoic acid (**1e**) under standard reaction conditions at 30 °C for 70 minutes (Fig. 1c). Remarkably, a primary KIE was observed at both C1 and C2 positions: KIEs of 1.014 ± 0.004 and 1.009 ± 0.006 were obtained for C1, while larger KIEs of 1.028 ± 0.004 and 1.025 ± 0.005 were measured for C2. These values are consistent with the proposed pathway in which a concerted decarboxylation-bromination transition state is involved in the product determining step (Fig. 1b). The lower KIE for C1 in comparison to C2 suggests either an early transition state,^27^ or that another kinetically relevant step is occurring prior to the product determining step.22e Examination of the reaction path by DFT (**TS-II** to **2e**) revealed an early formation of the C1–Br bond, resulting in an “hidden” Wheland intermediate.^28^ Extrusion of CO_2_ from this transient species took place late along the reaction coordinates, thus in agreement with the experimental observations.^29^ To further probe our mechanistic hypothesis, the KIE values for the proposed path were calculated (Fig. 1c). Computed and experimental values were found to be in excellent agreement, lending strong support to the proposed concerted decarboxylation-bromination pathway.

We have conducted a preliminary mechanistic study of the developed decarboxylative bromination of aromatic acids. At present, we have strong evidence that excludes the intermediacy of aryl radicals (Fig. 1a). By measuring the ^13^C/^12^C KIEs and performing DFT calculations we identified a concerted decarboxylation-bromination as a possible pathway for this transformation (Fig. 1b and c). This represents an alternative mechanism for Hunsdiecker-type reactivity, as radicals are usually considered key intermediates in similar processes. While further investigations are necessary to better establish the mechanism of this reaction, we believe that these initial studies highlight previously unrealised features of our system. We hope that these results inspire future studies that may greatly impact this and related procedures, and lead to the development of more efficient decarboxylative technologies.

## Conclusions

Due to slow progress and issues with selectivity, the utility of the aromatic Hunsdiecker reaction has previously failed to be fully realised. In this report we have detailed the successful development of a high yielding aromatic Hunsdiecker-type reaction. This has led to the development of a decarboxylative bromination of electron-rich aromatic acids using low-cost and abundant starting materials. The avoidance of transition metals and the ability to scale-up the reaction make the process attractive for its simplicity and low cost. The Hunsdiecker reaction is commonly proposed to proceed *via* a radical pathway, however, our combined experimental and theoretical mechanistic study has suggested an alternative mechanism that does not involve radical intermediates. These results directly challenge a long-held view of Hunsdiecker-type reactivity. Further studies are necessary, but we hope that future investigations will better elucidate this mechanism. Overall, we believe that this report demonstrates the potential of decarboxylative halogenation as an efficient route to value-added chemical commodities.

## Conflicts of interest

There are no conflicts to declare.

## Supplementary Material

Supplementary information
