# Supplementary material for: Transition-metal-free decarboxylative bromination of aromatic carboxylic acids
Source: Chem Sci. 2018 Mar 26;9(15):3860–5. doi: 10.1039/c8sc01016a (PMC5935059; doi:10.1039/c8sc01016a)
Supplement: Supplementary file 1 [file SC-009-C8SC01016A-s001.pdf]

Electronic Supplementary Material (ESI) for Chemical Science.
This journal is © The Royal Society of Chemistry 2018

SUPPORTING INFORMATION

Transition-Metal-Free Decarboxylative Bromination of Aromatic Carboxylic Acids

Jacob M. Quibell,[†] Gregory J. P. Perry,[†] Diego M. Cannas and Igor Larrosa*

School of Chemistry, University of Manchester, Oxford Road, Manchester, M13 9PL (UK)

*Corresponding Author: igor.larrosa@manchester.ac.uk
[†]G.J.P.P and J.M.Q contributed equally to this work.

Contents

General Information 3

General Procedure A for the Decarboxylative Bromination of Aromatic Acids 4

General Procedure B for the Decarboxylative Bromination of Aromatic Acids 4

Experimental details, Spectroscopic data and Analytical data 5

Procedure for Multi-Gram Scale Synthesis of 2-Bromo-1,3-dimethoxybenzene (2e) 21

Computed reaction path 22

Determination of ^{13}C-KIEs at natural abundance 24

  Experimental procedure 24

  Quantitative ^{13}C NMR 25

  Experiment 1 25

  Experiment 2 26

Calculated ^{13}C isotope effects for the proposed pathway 27

Coordinates and energies for the computed structures 28

NMR Spectra 33

References 79

General Information

Unless otherwise indicated, all reactions were carried out in 10 mL microwave vials using reagents obtained from commercial sources and used without further purification. K_3PO_4 was kept in a vacuum oven at 200 °C for 24 h prior to use and stored in a glove box. All other starting materials and solvents were purchased from Acros, Aldrich, Alfa Aesar, Fluorochem, Apollo Scientific and Manchester Organics, and used without further purification unless otherwise stated. 2-(allyloxy)-4-methoxybenzoic acid (**1A**) was synthesised according to a reported procedure.[1] Column chromatography was performed on silica gel (40–63 μm) or on a Biotage Isolera Four purification system using pre-packed silica cartridges. $AgNO_3$ impregnated silica gel was prepared by absorbing a solution of $AgNO_3$ in MeCN (10% wt of $AgNO_3$ to silica) on silica. The MeCN was removed under reduced pressure on a rotary evaporator and the silica was further dried under vacuum overnight. Thin layer chromatography (TLC) was carried out on pre-coated silica gel F254 plates with visualization under UV light or using an aqueous basic $KMnO_4$ solution. Melting points were obtained using a Stuart SMP11 apparatus and are uncorrected. IR spectra were recorded using a Thermo Scientific Nicolet iS5 FTIR machine, relevant bands are quoted in cm^{-1}. High resolution mass spectra were performed by the School of Chemistry Mass Spectrometry Service (University of Manchester) employing a Thermo Finnigan MAT95XP or Thermo Exactive Plus EMR spectrometer. ^{1}H NMR, ^{19}F NMR and ^{13}C NMR spectra were recorded at 400 or 500 MHz on Bruker machines. ^{1}H NMR are referenced to the residual solvent peak at 7.26 ppm ($CDCl_3$) and quoted in ppm to 2 decimal places with coupling constants (*J*) to the nearest 0.1 Hz. ^{13}C NMR spectra, recorded at 101 MHz or 126 MHz, are referenced to the solvent peak at 77.16 ppm ($CDCl_3$) and quoted in ppm to 1 decimal place with coupling constants (*J*) to the nearest 0.1 Hz. ^{19}F NMR spectra were recorded at 376 or 471 MHz in $CDCl_3$ and quoted in ppm to 2 decimal places and with coupling constants (*J*) to the nearest 0.1 Hz.

General Procedure A for the Decarboxylative Bromination of Aromatic Acids

A flame-dried 10 mL microwave vial was charged with a given amount of Bu_4NBr_3, capped and flushed with N_2. The vial was transferred to a glove box, then benzoic acid (0.50 mmol, 1.0 equiv), K_3PO_4 (106.1 mg, 0.50 mmol, 1.0 equiv) and MeCN (2.5 mL, 0.2 M) were added. The vial was capped, transferred out of the glove box and stirred at the given temperature for the given time. On completion of the reaction, the mixture was cooled to room temperature then 15% aq. $Na_2S_2O_3$ (10.0 mL) and sat. Na_2CO_3 (10.0 mL) were added. The organic phase was collected by washing with CH_2Cl_2 (3 x 15.0 mL), dried with $MgSO_4$ and concentrated *in vacuo*. The mixture was dissolved in pentane/EtOAc (5.0 mL, 98:2) and filtered through a short plug of celite with further washings of pentane/EtOAc (3 x 10.0 mL, 98:2) Removal of the solvent *in vacuo* gave the desired product.

General Procedure B for the Decarboxylative Bromination of Aromatic Acids

A flame-dried 10 mL microwave vial was charged with a given amount of Bu_4NBr_3, capped and flushed with N_2. The vial was transferred to a glove box, then benzoic acid (0.50 mmol, 1.0 equiv), K_3PO_4 (106.1 mg, 0.50 mmol, 1.0 equiv) and MeCN (2.5 mL, 0.2 M) were added. The vial was capped, transferred out of the glove box and stirred at the given temperature for the given time. On completion of the reaction, the mixture was cooled to room temperature then 15% aq. $Na_2S_2O_3$ (10.0 mL) and sat. Na_2CO_3 (10.0 mL) were added. The organic phase was collected by washing with pentane/EtOAc (3 x 15.0 mL, 95:5) and dried with $MgSO_4$. Removal of the solvent *in vacuo* gave the desired product.

Note on the use of glove box: while the reactions in the scope table were prepared in a glove box, this is not required, and similar yields are obtained when the reactions are set up without using any inert atmosphere. See page S21, for a large scale preparation of **2e** which was carried out under air.

Experimental details, Spectroscopic data and Analytical data

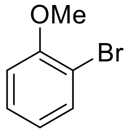

2-bromoanisole (2a)

The general procedure **A** was applied with Bu_4NBr_3 (241.1 mg, 0.50 mmol, 1.0 equiv) and 2-methoxybenzoic acid (76.1 mg, 0.50 mmol, 1.0 equiv) at 100 °C for 4 h. The general work-up procedure gave the desired product as a colourless oil (84.2 mg, 90%) as a >150:1 mixture with 2,4-dibromoanisole.

Spectroscopic data matched those previously reported.[2]

^{1}H NMR (500 MHz, $CDCl_3$) δ 7.54 (d, *J* = 7.8 Hz, 1H), 7.28 (app t, *J* = 7.9 Hz, 1H), 6.91 (d, *J* = 8.2 Hz, 1H), 6.84 (app t, *J* = 7.6 Hz, 1H), 3.90 (s, 3H); ^{13}C NMR (126 MHz, $CDCl_3$) δ 156.0, 133.5, 128.6, 121.9, 112.1, 111.8, 56.3.

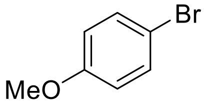

4-bromoanisole (2b)

The general procedure **A** was applied with Bu_4NBr_3 (482.2 mg, 1.00 mmol, 2.0 equiv) and 4-methoxybenzoic acid (76.1 mg, 0.50 mmol, 1.0 equiv) at 100 °C for 6 h. The general work-up procedure gave the desired product as a colourless oil (91.6 mg, 98%).

Spectroscopic data matched those previously reported.[3]

^{1}H NMR (500 MHz, $CDCl_3$) δ 7.38 (d, *J* = 8.0 Hz, 2H), 6.79 (d, *J* = 8.0 Hz, 2H), 3.78 (s, 3H); ^{13}C NMR (126 MHz, $CDCl_3$) δ 158.8, 132.4, 115.9, 112.9, 55.6.

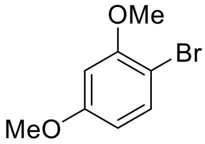

1-bromo-2,4-dimethoxybenzene (2d)

The general procedure **A** was applied with Bu_4NBr_3 (241.1 mg, 0.50 mmol, 1.0 equiv) and 2,4-dimethoxybenzoic acid (91.1 mg, 0.50 mmol, 1.0 equiv) for 16 h at 23 °C. The general work-up procedure gave the desired product as a colourless oil (102.0 mg, 94%) as a >45:1 mixture with 1,5-dibromo-2,4-dimethoxybenzene.

Spectroscopic data matched those previously reported.[4]

^{1}H NMR (500 MHz, $CDCl_3$) δ 7.41 (d, *J* = 8.5 Hz, 1H), 6.49 (d, *J* = 2.1 Hz, 1H), 6.39 (dd, *J* = 8.5, 2.1 Hz, 1H), 3.87 (s, 3H), 3.80 (s, 3H); ^{13}C NMR (126 MHz, $CDCl_3$) δ 160.4, 156.7, 133.3, 106.0, 102.6, 100.1, 56.3, 55.7.

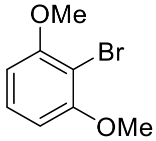

2-bromo-1,3-dimethoxybenzene (2e)

The general procedure A was applied with Bu_4NBr_3 (241.1 mg, 0.5 mmol, 1.0 equiv) and 2,6-dimethoxybenzoic acid (91.1 mg, 0.50 mmol, 1.0 equiv) at 100 °C for 16 h. The general work-up procedure gave the desired product as a colourless solid (96.6 mg, 89%)

Spectroscopic data matched those previously reported.[5]

^{1}H NMR (500 MHz, $CDCl_3$) δ 7.23 (t, *J* = 8.4 Hz, 1H), 6.58 (d, *J* = 8.4 Hz, 2H), 3.90 (s, 6H); ^{13}C NMR (101 MHz, $CDCl_3$) δ 157.3, 128.4, 104.8, 101.1, 56.6.

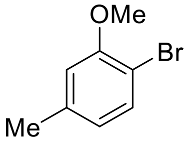

1-bromo-2-methoxy-4-methylbenzene (2f)

The general procedure **A** was applied with Bu_4NBr_3 (241.1 mg, 0.5 mmol, 1.0 equiv) and 2-methoxy-4-methylbenzoic acid (83.1 mg, 0.50 mmol, 1.0 equiv) at 100 °C for 16 h. The general work-up procedure gave the desired product as a colourless oil (85.5 mg, 85%)

Spectroscopic data matched those previously reported.[6]

^{1}H NMR (500 MHz, $CDCl_3$) δ 7.39 (d, *J* = 8.0 Hz, 1H), 6.72 (s, 1H), 6.66 (d, *J* = 8.0 Hz, 1H), 3.88 (s, 3H), 2.32 (s, 3H); ^{13}C NMR (126 MHz, $CDCl_3$) δ 155.7, 138.9, 133.0, 122.7, 113.1, 108.4, 56.2, 21.6.

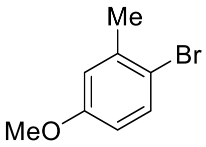

1-bromo-4-methoxy-2-methylbenzene (2g)

The general procedure **A** was applied with Bu_4NBr_3 (241.1 mg, 0.5 mmol, 1.0 equiv) and 4-methoxy-2-methylbenzoic acid (83.1 mg, 0.50 mmol, 1.0 equiv) for 16 h. The general work-up procedure gave the desired product as a colourless oil (98.5 mg, 98%).

Spectroscopic data matched those previously reported.[7]

^{1}H NMR (500 MHz, $CDCl_3$) δ 7.40 (d, *J* = 8.7 Hz, 1H), 6.79 (s, 1H), 6.62 (d, *J* = 8.7 Hz, 1H), 3.78 (s, 3H), 2.37 (s, 3H); ^{13}C NMR (126 MHz, $CDCl_3$) δ 158.9, 138.9, 132.9, 116.6, 115.5, 113.0, 55.5, 23.3.

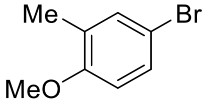

4-bromo-1-methoxy-2-methylbenzene (2h)

The general procedure **A** was applied with Bu_4NBr_3 (241.1 mg, 0.5 mmol, 1.0 equiv) and 3-methyl-4-methoxy benzoic acid (83.1 mg, 0.50 mmol, 1.0 equiv) at 100 °C for 16 h. The general work-up procedure was followed gave the desired product as a colourless solid (77.4 mg, 77%)

Spectroscopic data matched those previously reported.[7]

^1H NMR (500 MHz, $CDCl_3$) δ 7.28 – 7.26 (m, 2H), 6.70 (d, *J* = 8.2 Hz, 1H), 3.82 (s, 3H), 2.20 (s, 3H). ^{13}C NMR (126 MHz, $CDCl_3$) δ 157.0, 133.3, 129.5, 129.1, 112.5, 111.6, 55.6, 16.2.

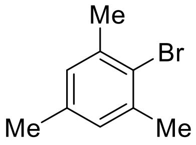

2-bromo-1,3,5-trimethylbenzene (2i)

The general procedure **B** was applied with Bu_4NBr_3 (482.2 mg, 1.0 mmol, 2.0 equiv) and 2,4,6-trimethylbenzoic acid (82.1 mg, 0.50 mmol, 1.0 equiv) at 50 °C for 16 h. The general work-up procedure gave the desired product as a colourless oil (93.6 mg, 94%).

Spectroscopic data matched those previously reported.[8]

^1H NMR (500 MHz, $CDCl_3$) δ 6.90 (s, 2H), 2.38 (s, 6H), 2.24 (s, 3H); ^{13}C NMR (126 MHz, $CDCl_3$) δ 138.0, 136.4, 129.2, 124.3, 23.9, 20.8.

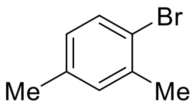

1-bromo-2,4-dimethylbenzene (2j)

The general procedure **B** was applied with Bu_4NBr_3 (482.2 mg, 1.0 mmol, 2.0 equiv) and 2,4-dimethylbenzoic acid (75.1 mg, 0.50 mmol, 1.0 equiv) at 100 °C for 16 h. The general work-up procedure was followed then the mixture was purified by column chromatography (Hexane 100%). Removal of the solvent *in vacuo* gave the desired product as a yellow oil (74.0 mg, 80%) as a >100:1 mixture with 1,5-dibromo-2,4-dimethylbenzene.

Spectroscopic data matched those previously reported.[9]

^1H NMR (400 MHz, $CDCl_3$) δ 7.39 (d, *J* = 8.1 Hz, 1H), 7.05 (s, 1H), 6.86 (d, *J* = 8.1 Hz, 1H), 2.36 (s, 3H), 2.27 (s, 3H); ^{13}C NMR (126 MHz, $CDCl_3$) δ 137.4, 137.0, 132.0, 131.7, 128.1, 121.5, 22.8, 20.8.

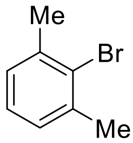

2-bromo-1,3-dimethylbenzene (2k)

The general procedure **B** was applied with Bu_4NBr_3 (482.2 mg, 1.0 mmol, 2.0 equiv) and 2,6-dimethylbenzoic acid (75.1 mg, 0.50 mmol, 1.0 equiv) at 100 °C for 16 h. The general work-up procedure gave the desired product as a yellow oil (79.6 mg, 86%) as a 50:1 mixture with 1,3-dibromo-2,4-dimethylbenzene.

Spectroscopic data matched those previously reported.[10]

^1H NMR (500 MHz, $CDCl_3$) δ 7.14 – 7.01 (m, 3H), 2.42 (s, 6H); ^{13}C NMR (126 MHz, $CDCl_3$) δ 138.4, 128.3, 127.8, 126.7, 24.0.

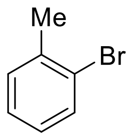

1-bromo-2-methylbenzene (2l)

The general procedure **B** was applied with Bu_4NBr_3 (723.3 mg, 1.5 mmol, 3.0 equiv) and 2-methylbenzoic acid (68.1 mg, 0.50 mmol, 1.0 equiv) at 100 °C for 16 h. On completion of the reaction, the mixture was cooled to room temperature then 15% aq. $Na_2S_2O_3$ (10.0 mL) and sat. Na_2CO_3 (10.0 mL) were added. The organic phase was collected by washing with Chloroform (3 x 15.0 mL), dried with $MgSO_4$ and concentrated *in vacuo*. The product was then purified by column chromatography (Hexane 100%). Removal of the solvent *in vacuo* gave the desired product as a colourless oil (45.3 mg, 53%). Due to the volatility of 1-bromo-2-methylbenzene (**2l**), this product could not be isolated with a yield comparable to the ^{1}H NMR yield (78%).

Spectroscopic data matched those previously reported.[11]

^{1}H NMR (500 MHz, $CDCl_3$) δ 7.54 (d, *J* = 7.7 Hz, 1H), 7.29 – 7.17 (m, 2H), 7.06 (app t, *J* = 7.4 Hz, 1H), 2.42 (s, 3H); ^{13}C NMR (126 MHz, $CDCl_3$) δ 138.0, 132.5, 131.0, 127.5, 127.4, 125.1, 23.1.

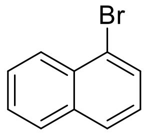

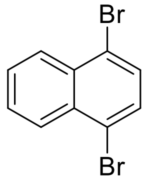

1-bromonaphthalene and 1,4-dibromonaphthalene (2m and 2m')

The general procedure **B** was applied with Bu_4NBr_3 (723.3 mg, 1.5 mmol, 3.0 equiv) and 1-naphthoic acid (86.1 mg, 0.50 mmol, 1.0 equiv) at 100 °C for 16 h. The general work-up procedure gave the desired product as a yellow oil (59.2 mg, 57%) as a 14:1 mixture with 1,8-dibromonaphthalene. The mixture was purified by column chromatography (Hexane 100%). Removal of the solvent *in vacuo* gave 1-bromonaphthalene as a yellow oil (55.9 mg, 54%) and 1,8-dibromonaphthalene as a white solid (4.3 mg, 3%).

Spectroscopic data matched those previously reported.[12]

1-bromonaphthalene (2m)

^{1}H NMR (500 MHz, $CDCl_3$) δ 8.25 (d, *J* = 8.5 Hz, 1H), 7.83 (app dd, *J* = 11.8, 8.2 Hz, 2H), 7.79 (d, *J* = 7.4 Hz, 1H), 7.60 (app t, *J* = 7.6 Hz, 1H), 7.54 (app t, *J* = 7.5 Hz, 1H), 7.33 (app t, *J* = 7.8 Hz, 1H); ^{13}C NMR (126 MHz, $CDCl_3$) δ 134.8, 132.1, 130.1, 128.5, 128.1, 127.5, 127.3, 126.9, 126.3, 123.0.

1,8-dibromonaphthalene (2m')

^{1}H NMR (400 MHz, $CDCl_3$) δ 8.28 – 8.25 (m, 2H), 7.69 – 7.62 (m, 4H); ^{13}C NMR (126 MHz, $CDCl_3$) δ 133.1, 130.2, 128.3, 127.9, 122.7.

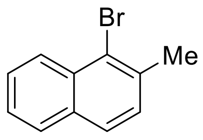

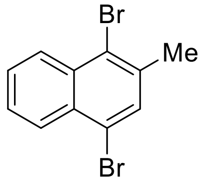

1-bromo-2-methylnaphthalene and 1,4-dibromo-2-methylnaphthalene (2n and 2n')

The general procedure **B** was applied with Bu_4NBr_3 (723.3 mg, 1.5 mmol, 3.0 equiv) and 2-methyl-1-naphthoic acid (93.1 mg, 0.50 mmol, 1.0 equiv) at 120 °C for 16 h. The general work-up procedure gave the desired product as a yellow oil (74.1 mg, 67%) as a 22:1 mixture with 1,4-dibromo-2-methylnaphthalene. The mixture was purified by column chromatography (Hexane 100 %). Removal of the solvent *in vacuo* gave the desired product as a colourless oil (70.8 mg, 64%) and 1,4-dibromo-2-methylnaphthalene as a white solid (3.0 mg, 2%).

Spectroscopic data matched those previously reported.[13]

1-bromo-2-methylnaphthalene (2n)

^{1}H NMR (500 MHz, $CDCl_3$ δ 8.31 (d, *J* = 8.6 Hz, 1H), 7.80 (d, *J* = 8.1 Hz, 1H), 7.72 (d, *J* = 8.3 Hz, 1H), 7.58 (app t, *J* = 7.7 Hz, 1H), 7.47 (app t, *J* = 7.5 Hz, 1H), 7.36 (d, *J* = 8.3 Hz, 1H), 2.64 (s, 3H); ^{13}C NMR (126 MHz, $CDCl_3$) δ 136.1, 133.1, 132.7, 128.8, 128.1, 127.4, 127.4, 127.1, 125.8, 124.2, 24.3.

1,4-dibromo-2-methylnaphthalene (2n')

^{1}H NMR (400 MHz, $CDCl_3$) δ 8.25 (d, *J* = 8.0 Hz, 1H), 8.13 (d, *J* = 8.0 Hz, 1H), 7.63 (s, 1H), 7.53 (app p, *J* = 7.2 Hz, 2H), 2.54 (s, 3H); ^{13}C NMR (101 MHz, $CDCl_3$) δ 136.1, 133.1, 132.7, 128.8, 128.1, 127.4, 127.4, 127.4, 127.1, 125.8, 124.2, 24.3.

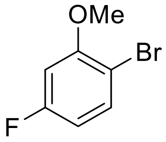

1-bromo-4-fluoro-2-methoxybenzene (2o)

The general procedure **A** was applied with Bu_4NBr_3 (241.1 mg, 0.5 mmol, 1.0 equiv) and 4-fluoro-2-methoxybenzoic acid (85.1 mg, 0.50 mmol, 1.0 equiv) at 100 °C for 16 h. The general work-up procedure gave the desired product as a colourless oil (76.9 mg, 75%).

Spectroscopic data matched those previously reported.[14]

^{1}H NMR (500 MHz, $CDCl_3$) δ 7.40 (dd, *J* = 8.7, 6.2 Hz, 1H), 6.58 (dd, *J* = 10.4, 2.7 Hz, 1H), 6.51 (app td, *J* = 8.3, 2.7 Hz, 1H), 3.81 (s, 3H). ^{13}C NMR (126 MHz, $CDCl_3$) δ 163.0 (d, *J* = 246.2 Hz), 157.0 (d, *J* = 10.0 Hz), 133.7 (d, *J* = 9.6 Hz), 108.5 (d, *J* = 22.5 Hz), 106.0 (d, *J* = 3.6 Hz), 100.6 (d, *J* = 26.9 Hz), 56.5; ^{19}F NMR (471 MHz, CDCl) δ -111.76 (ddd, *J* = 10.5, 7.9, 6.2 Hz).

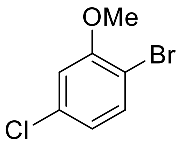

1-bromo-4-chloro-2-methoxybenzene (2p)

The general procedure **A** was applied with Bu_4NBr_3 (241.1 mg, 0.5 mmol, 1.0 equiv) and 4-chloro-2-methoxybenzoic acid (93.3 mg, 0.50 mmol, 1.0

equiv) at 100 °C for 16 h. The general work-up procedure gave the desired product as a colourless oil (91.9 mg, 83%)

Spectroscopic data matched those previously reported.[15]

^{1}H NMR (500 MHz, $CDCl_3$) δ 7.45 (d, *J* = 8.4 Hz, 1H), 6.88 (d, *J* = 2.2 Hz, 1H), 6.84 (dd, *J* = 8.4, 2.1 Hz, 1H), 3.89 (s, 3H); ^{13}C NMR (126 MHz, $CDCl_3$) δ 156.6, 134.2, 133.9, 121.9, 112.8, 109.9, 56.6.

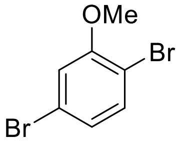

1,4-dibromo-2-methoxybenzene (2q)

The general procedure **A** was applied with Bu_4NBr_3 (241.1 mg, 0.5 mmol, 1.0 equiv) and 4-bromo-2-methoxybenzoic acid (115.5 mg, 0.50 mmol, 1.0 equiv) at 100 °C for 16 h. The general work-up procedure gave the desired product as a colourless solid (95.7 mg, 72%).

Spectroscopic data matched those previously reported.[16]

^{1}H NMR (400 MHz, $CDCl_3$) δ 7.38 (d, *J* = 8.3 Hz, 1H), 7.01 (d, *J* = 2.0 Hz, 1H), 6.97 (dd, *J* = 8.3, 2.1 Hz, 1H), 3.89 (s, 3H); ^{13}C NMR (101 MHz, $CDCl_3$) δ 156.6, 134.2, 124.9, 121.7, 115.5, 110.6, 56.6.

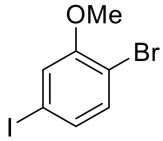

1-bromo-4-iodo-2-methoxybenzene (2r)

The general procedure **A** was applied with Bu_4NBr_3 (241.1 mg, 0.5 mmol, 1.0 equiv) and 4-iodo-2-methoxybenzoic acid (139.0 mg, 0.50 mmol, 1.0 equiv) at 100 °C for 16 h. The general work-up procedure gave the desired product as a colourless solid (132.9 mg, 85%).

Spectroscopic data matched those previously reported.[17]

^{1}H NMR (500 MHz, $CDCl_3$) δ 7.24 (d, *J* = 8.0 Hz, 1H), 7.19 – 7.13 (m, 2H), 3.88 (s, 3H); ^{13}C NMR (126 MHz, $CDCl_3$) δ 156.6, 134.7, 131.1, 121.4, 112.0, 92.6, 56.6.

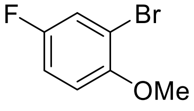

2-bromo-4-fluoro-1-methoxybenzene (2s)

The general procedure **B** was applied with Bu_4NBr_3 (482.2 mg, 1.0 mmol, 2.0 equiv) and 5-fluoro-2-methoxybenzoic acid (85.1 mg, 0.50 mmol, 1.0 equiv) at 100 °C for 16 h. The general work-up procedure gave the desired product as a yellow oil (85.1 mg, 83%).

Spectroscopic data matched those previously reported.[18]

^{1}H NMR (500 MHz, $CDCl_3$) δ 7.30 (dd, *J* = 7.8, 3.0 Hz, 1H), 6.99 (ddd, *J* = 8.4, 7.9, 3.0 Hz, 1H), 6.83 (dd, *J* = 9.1, 4.7 Hz, 1H), 3.86 (s, 3H); ^{13}C NMR (126 MHz, $CDCl_3$) δ 156.7 (d, *J* = 242.6 Hz), 152.7 (d, *J* = 2.6 Hz), 120.6 (d, *J* = 25.8 Hz), 114.8 (d, *J* = 22.5 Hz), 112.4 (d, *J* = 8.4 Hz), 111.8 (d, *J* = 9.8 Hz), 56.9; ^{19}F NMR (471 MHz, $CDCl_3$) δ -122.12 – -122.20 (m).

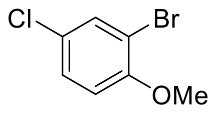

2-bromo-4-chloro-1-methoxybenzene (2t)

The general procedure **B** was applied with Bu_4NBr_3 (482.2 mg, 1.0 mmol, 2.0 equiv) and 5-chloro-2-methoxybenzoic acid (93.3 mg, 0.50 mmol, 1.0 equiv) at 100 °C for 16 h. The general work-up procedure gave the desired product as a yellow oil (96.3 mg, 87%).

Spectroscopic data matched those previously reported.[19]

^{1}H NMR (500 MHz, $CDCl_3$) δ 7.53 (d, *J* = 2.4 Hz, 1H), 7.23 (dd, *J* = 8.8, 2.4 Hz, 1H), 6.81 (d, *J* = 8.8 Hz, 1H), 3.87 (s, 3H); ^{13}C NMR (126 MHz, $CDCl_3$) δ 154.8, 132.9, 128.4, 126.0, 112.6, 112.1, 56.6.

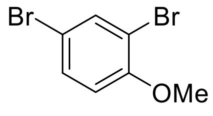

2,4-dibromo-1-methoxybenzene (2u)

The general procedure **B** was applied with Bu_4NBr_3 (482.2 mg, 1.0 mmol, 2.0 equiv) and 5-bromo-2-methoxybenzoic acid (115.5 mg, 0.50 mmol, 1.0 equiv) at 100 °C for 16 h. The general work-up gave the desired product as a red oil (117.0 mg, 88%).

Spectroscopic data matched those previously reported.[8]

^{1}H NMR (500 MHz, $CDCl_3$) δ^{1}H NMR (400 MHz, $CDCl_3$ δ 7.66 (d, *J* = 2.4 Hz, 1H), 7.37 (dd, *J* = 8.8, 2.4 Hz, 1H), 6.76 (d, *J* = 8.8 Hz, 1H), 3.87 (s, 3H); ^{13}C NMR (101 MHz, $CDCl_3$) δ 155.2, 135.5, 131.2, 113.1, 112.9, 112.6, 56.4.

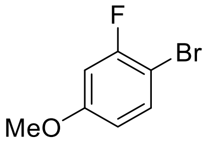

1-bromo-2-fluoro-4-methoxybenzene (2v)

The general procedure **B** was applied with Bu_4NBr_3 (482.2 mg, 1.0 mmol, 2.0 equiv) and 2-fluoro-4-methoxybenzoic acid (85.1 mg, 0.50 mmol, 1.0 equiv) at 100 °C for 16 h. The general work-up procedure gave the desired product as a yellow oil (92.3 mg, 90%).

Spectroscopic data matched those previously reported.[20]

^{1}H NMR (500 MHz, $CDCl_3$) ^{1}H NMR (500 MHz, $CDCl_3$) δ 7.49 – 7.36 (m, 1H), 6.69 (dd, *J* = 10.4, 2.8 Hz, 1H), 6.61 (ddd, *J* = 8.9, 2.8, 0.9 Hz, 1H), 3.78 (s, 3H); δ ^{13}C NMR (126 MHz, $CDCl_3$) δ 160.5 (d, *J* = 27.2 Hz), 159.5 (d, *J* = 209.3 Hz), 133.4 (d, *J* = 1.9 Hz), 111.5 (d, *J* =

3.1 Hz), 103.0 (d, J = 25.6 Hz), 99.4 (d, J = 21.2 Hz), 55.9.; ^{19}F NMR (471 MHz, $CDCl_3$) δ -104.78 – -106.51 (m).

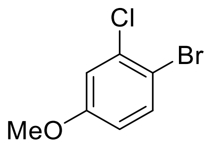

1-bromo-2-chloro-4-methoxybenzene (2w)

The general procedure **A** was applied with Bu_4NBr_3 (482.2 mg, 1.0 mmol, 2.0 equiv) and 2-chloro-4-methoxybenzoic acid (93.3 mg, 0.50 mmol, 1.0 equiv) at 100 °C for 16 h. The general work-up procedure gave the desired product as a yellow oil (105.2 mg, 95%)

Spectroscopic data matched those previously reported.[21]

^{1}H NMR (500 MHz, $CDCl_3$) δ 7.47 (d, J = 8.9 Hz, 1H), 7.01 (d, J = 2.9 Hz, 1H), 6.70 (dd, J = 8.9, 2.9 Hz, 1H), 3.78 (s, 3H); ^{13}C NMR (126 MHz, $CDCl_3$) δ 159.5, 135.0, 134.0, 115.9, 114.6, 113.0, 55.8.

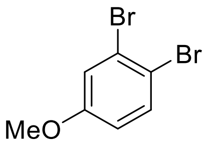

1,2-dibromo-4-methoxybenzene (2x)

The general procedure **A** was applied with Bu_4NBr_3 (482.2 mg, 1.0 mmol, 2.0 equiv) and 2-bromo-4-methoxybenzoic acid (115.5 mg, 0.50 mmol, 1.0 equiv) at 100 °C for 16 h. The general work-up procedure gave the desired product as a yellow oil (130.3 mg, 98%).

Spectroscopic data matched those previously reported.[21]

^{1}H NMR (500 MHz, $CDCl_3$) δ 7.48 (d, J = 8.9 Hz, 1H), 7.17 (d, J = 2.6 Hz, 1H), 6.74 (dd, J = 8.8, 2.6 Hz, 1H), 3.78 (s, 3H); ^{13}C NMR (126 MHz, $CDCl_3$) δ 159.3, 133.9, 125.1, 119.1, 115.4, 115.3, 55.9.

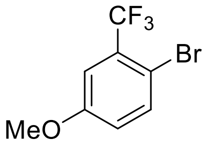

1-bromo-4-methoxy-2-(trifluoromethyl)benzene (2y)

The general procedure **A** was applied with Bu_4NBr_3 (482.2 mg, 1.0 mmol, 2.0 equiv) and 2-(trifluoromethyl)-4-methoxybenzoic acid (110.1 mg, 0.50 mmol, 1.0 equiv) at 100 °C for 16 h. The general work-up procedure gave the desired product as a yellow oil (122.4 mg, 96%).

^{1}H NMR (500 MHz, $CDCl_3$ δ 7.58 (d, J = 8.8 Hz, 1H), 7.22 (d, J = 2.9 Hz, 1H), 6.92 (dd, J = 8.8, 2.9 Hz, 1H), 3.83 (s, 3H); ^{13}C NMR (126 MHz, $CDCl_3$) δ 158.8, 135.9, 131.0 (q, J = 31.4 Hz), 122.8 (q, J = 273.5 Hz), 118.5, 114.0 (q, J = 5.6 Hz), 110.0, 55.9; ^{19}F NMR (376 MHz, $CDCl_3$) δ –62.90; IR (ATR) 3012, 2942, 2840, 1331, 1259, 1130, 1036, 882; HRMS (EI) *m/z* calcd. $C_8H_6BrF_3$: 253.9549; found $[M]^+$ 253.9549.

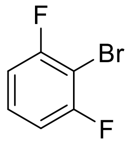

2-bromo-1,3-difluorobenzene (2ad)

The general procedure **A** was applied with Bu_4NBr_3 (964.4 mg, 2.00 mmol, 4.0 equiv), 2,6-difluorobenzoic acid (79.1 mg, 0.50 mmol, 1.0 equiv) at 100 °C for 20 h. Then 15% aq. $Na_2S_2O_3$ (2.0 mL), $CDCl_3$ (1.0 mL) and fluorobenzene (46.9 µL, 0.50 mmol, 1.0 equiv) were added. An aliquot (200 µL) of the organic layer was passed through a plug of $MgSO_4$ directly into an NMR tube and diluted with $CDCl_3$ (400 µL) for quantitative ^{19}F NMR analysis to yield the crude product (99%). Due to the volatility of 2-bromo-1,3-difluorobenzene **2ad**, this product could not be isolated with a yield comparable to the ^{19}F NMR yield.

Spectroscopic data matched those previously reported.[22]

Quantitative ^{19}F NMR ($CDCl_3$)

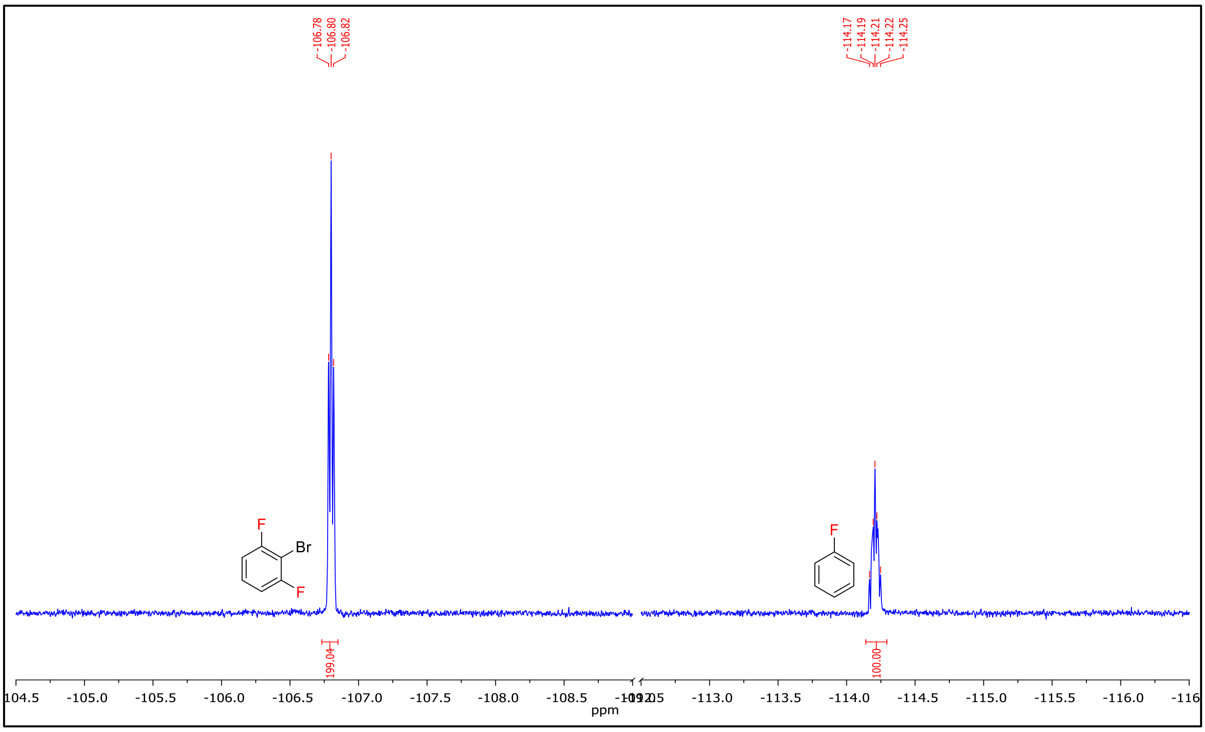

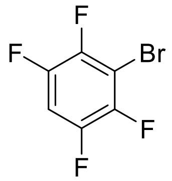

3-bromo-1,2,4,5-tetrafluorobenzene (2ae)

The general procedure **A** was applied with Bu_4NBr_3 (385.7 mg, 0.8 mmol, 4.0 equiv) 2,3,5,6-tetrafluorobenzoic acid (38.9 mg, 0.20 mmol, 1.0 equiv) at 100 °C for 16 h. Then 15% aq. $Na_2S_2O_3$ (2.0 mL), $CDCl_3$ (1.0 mL) and 1,4-difluorobenzene (6.3 µL, 0.08 mmol, 0.4 equiv) were added. An aliquot (200 µL) of the organic layer was passed through a plug of $MgSO_4$ directly into an NMR tube and diluted with $CDCl_3$ (400 µL) for quantitative ^{19}F NMR analysis to yield the crude product (71%) as a mixture with 1,4-dibromo-2,3,5,6-tetrafluorobenzene (4%). Due to the volatility of 3-bromo-1,2,4,5-tetrafluorobenzene **2ae**, this product could not be isolated with a yield comparable to the ^{19}F NMR yield.

Spectroscopic data matched those previously reported.[23]

Quantitative ^{19}F NMR ($CDCl_3$)

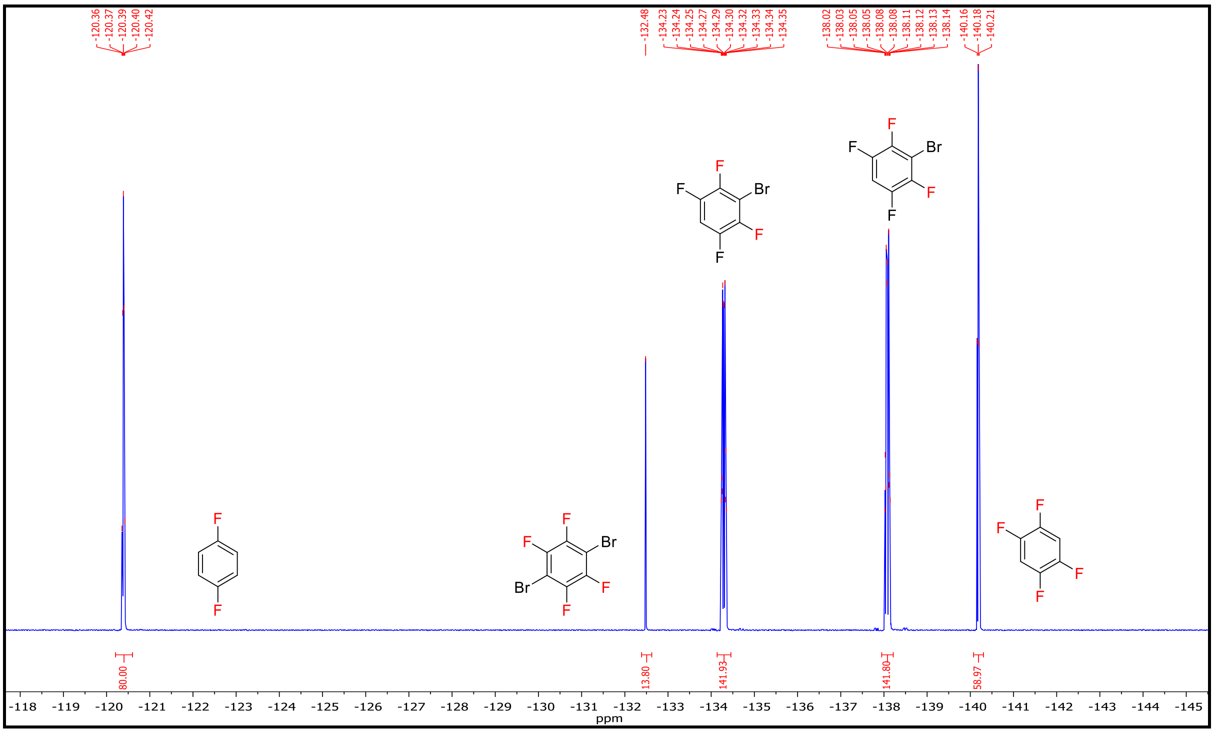

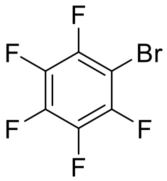

1-bromo-2,3,4,5,6-pentafluorobenzene (2af)

The general procedure **A** was applied with Bu_4NBr_3 (385.7 mg, 0.8 mmol, 4.0 equiv) 2,3,4,5,6-pentafluorobenzoic acid (42.4 mg, 0.20 mmol, 1.0 equiv) at 100 °C for 16 h. Then 15% aq. $Na_2S_2O_3$ (2.0 mL), $CDCl_3$ (1.0 mL) and 1,4-difluorobenzene (6.3 µL, 0.08 mmol, 0.4 equiv) were added. An aliquot (200 µL) of the organic layer was passed through a plug of $MgSO_4$ directly into an NMR tube and diluted with $CDCl_3$ (400 µL) for quantitative ^{19}F NMR analysis to yield the crude product (84%). Due to the volatility of 1-bromo-2,3,4,5,6-pentafluorobenzene **2af**, this product could not be isolated with a yield comparable to the ^{19}F NMR yield.

Spectroscopic data matched those previously reported.[24]

Quantitative ^{19}F NMR ($CDCl_3$)

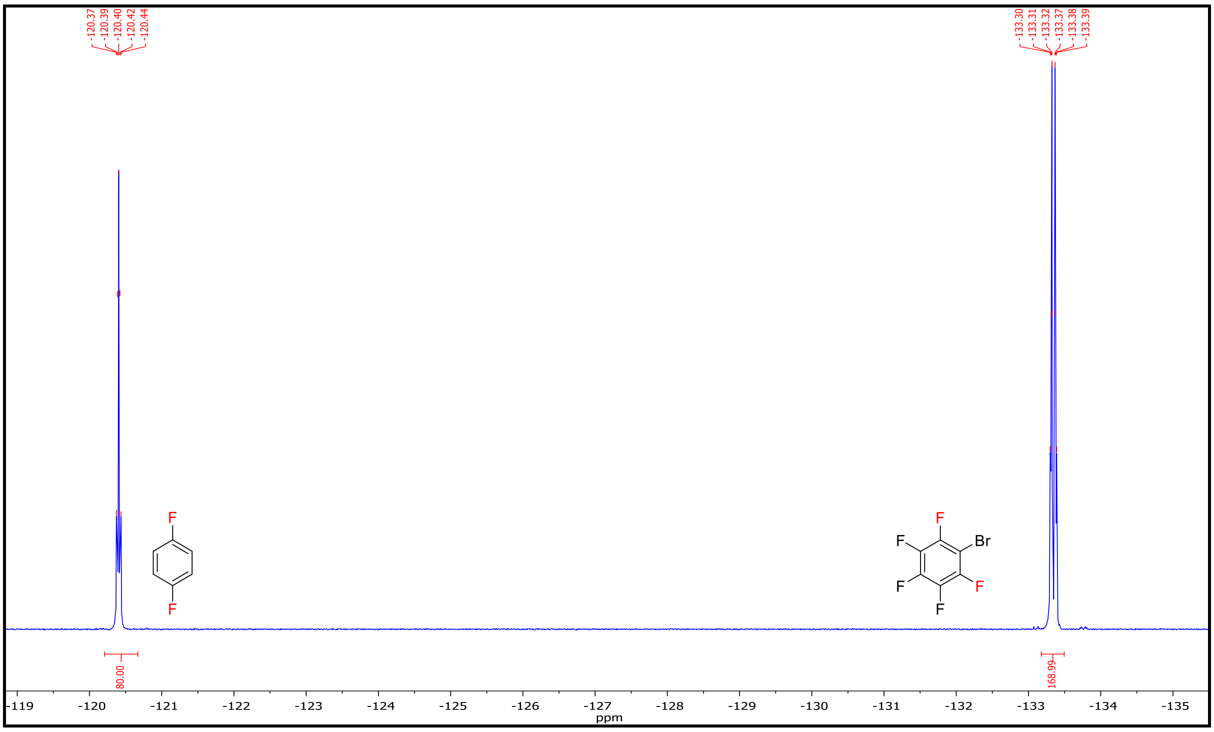

3-bromo-1-tosyl-1H-indole (4a)

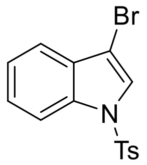

The general procedure **A** was applied with Bu_4NBr_3 (241.1 mg, 0.50 mmol, 1.0 equiv) and 1-tosyl-1H-indole-3-carboxylic acid (157.7 mg, 0.50 mmol, 1.0 equiv) at 100 °C for 2 h. The general work-up procedure gave the desired product as a white solid (155.8 mg, 89%).

Spectroscopic data matched those previously reported.[25]

^{1}H NMR (500 MHz, $CDCl_3$) δ 8.00 (d, *J* = 8.3 Hz, 1H), 7.77 (app d, *J* = 8.4 Hz, 2H), 7.62 (s, 1H), 7.49 (d, *J* = 7.9 Hz, 1H), 7.39 - 7.36 (m, 1H), 7.33 - 7.29 (m, 1H), 7.24 (app d, *J* = 8.0 Hz, 2H), 2.35 (s, 3H); ^{13}C NMR (126 MHz, $CDCl_3$) δ 145.5, 135.0, 134.4, 130.2, 129.9, 127.1, 125.9, 124.9, 124.0, 120.2, 113.7, 99.7, 21.7.

2-bromo-3-methylbenzo[b]thiophene (4b)

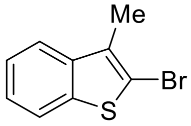

The general procedure **A** was applied with Bu_4NBr_3 (482.2 mg, 1.0 mmol, 2.0 equiv) and 3-methylbenzo[b]thiophene-2-carboxylic acid (96.1 mg, 0.50 mmol, 1.0 equiv) at 100 °C for 16 h. The general work-up procedure was gave the desired product as a yellow oil (102.2 mg, 90%)

Spectroscopic data matched those previously reported.[5]

^{1}H NMR (500 MHz, $CDCl_3$) δ 7.72 (d, *J* = 7.6 Hz, 1H), 7.64 (d, *J* = 7.6 Hz, 1H), 7.36 (app td, *J* = 7.6, 1.2 Hz, 1H), 7.32 (app td, *J* = 7.6, 1.2 Hz, 1H), 2.38 (s, 3H); ^{13}C NMR (126 MHz, $CDCl_3$) δ 139.8, 139.1, 131.9, 124.7, 124.6, 121.9, 121.8, 112.6, 13.3.

3-bromobenzo[b]thiophene (4c)

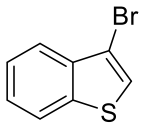

The general procedure **B** was applied with Bu_4NBr_3 (482.2 mg, 1.0 mmol, 2.0 equiv) and benzo[b]thiophene-3-carboxylic acid (89.1 mg, 0.50 mmol, 1.0 equiv) at 100 °C for 16 h. The general work-up procedure was followed then the mixture was purified by column chromatography (Hexane 100 %). Removal of the solvent *in vacuo* gave the desired product as a colourless oil (95.9 mg, 90%) as a >100:1 mixture with 2,3-dibromobenzo[b]thiophene.

Spectroscopic data matched those previously reported.[26]

^{1}H NMR (400 MHz, $CDCl_3$) δ 7.90 – 7.80 (m, 2H), 7.48 (app t, *J* = 7.5 Hz, 1H), 7.45 (s, 1H), 7.42 (app t, *J* = 7.5 Hz, 1H); ^{13}C NMR (126 MHz, $CDCl_3$) δ 138.7, 137.6, 125.4, 125.2, 123.6, 123.2, 122.9, 107.8.

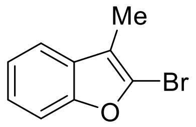

2-bromo-3-methylbenzofuran (4d)

The general procedure **B** was applied with Bu_4NBr_3 (482.2 mg, 1.0 mmol, 2.0 equiv) and 3-methylbenzofuran-2-carboxylic acid (88.1 mg, 0.50 mmol, 1.0 equiv) at 100 °C for 16 h. The general work-up procedure gave the desired product as a yellow oil (102.4 mg, 97%).

Spectroscopic data matched those previously reported.[5]

^{1}H NMR (500 MHz, $CDCl_3$) δ 7.47 – 7.38 (m, 2H), 7.30 – 7.16 (m, 2H), 2.20 (s, 3H); ^{13}C NMR (126 MHz, $CDCl_3$) δ 155.2, 129.5, 126.4, 124.3, 123.1, 118.8, 115.3, 111.0, 8.9.

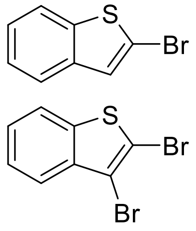

2-bromobenzo[b]thiophene and 2,3-dibromobenzo[b]thiophene (4e + 4e')

The general procedure **B** was applied with Bu_4NBr_3 (241.1 mg, 0.5 mmol, 1.0 equiv) and benzo[b]thiophene-2-carboxylic acid (89.1 mg, 0.50 mmol, 1.0 equiv) at 100 °C for 16 h. The general work-up procedure gave the desired product as a colourless oil (89.2 mg, 70%) as a (1:1) mixture with 2,3-dibromobenzo[b]thiophene. The mixture was then purified by column chromatography with 10% $AgNO_3$-impregnated silica (hexane/EtOAc, 95:5). Removal of the solvent *in vacuo* gave the desired product as a colourless solid (28.8 mg, 27%). A sample of 2,3-dibromobenzo[b]thiophene was also isolated for characterisation.

Spectroscopic data matched those previously reported.[27]

2-bromobenzo[b]thiophene (4e)

^{1}H NMR (400 MHz, $CDCl_3$) δ 7.77 – 7.66 (m, 2H), 7.39 – 7.29 (m, 3H); ^{13}C NMR (101 MHz, $CDCl_3$) δ 141.0, 139.6, 126.7, 124.9, 124.6, 122.8, 121.7, 115.5.

2,3-dibromobenzo[b]thiophene (4e')

^{1}H NMR (400 MHz, $CDCl_3$) δ 7.75 (d, J = 7.6 Hz, 1H), 7.72 (d, J = 7.6 Hz, 1H), 7.43 (td, J = 7.6, 1.3 Hz, 1H), 7.38 (td, J = 7.6, 1.4 Hz, 1H); ^{13}C NMR (101 MHz, $CDCl_3$) δ 139.0, 137.7, 125.8, 125.7, 123.5, 122.0, 114.4, 111.9.

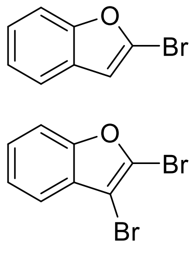

2-bromobenzofuran and 2,3-dibromobenzofuran (4f and 4f')

The general procedure **B** was applied with Bu_4NBr_3 (241.1 mg, 0.5 mmol, 1.0 equiv) and benzofuran-2-carboxylic acid (81.1 mg, 0.50 mmol, 1.0 equiv) at 100 °C for 6 h The general work-up procedure gave the desired product as a colourless oil (58.1 mg, 55%) as a 4.5:1 mixture with 2,3-dibromobenzofuran. Then the mixture was purified by column chromatography with 10% $AgNO_3$-impregnated silica (hexane/EtOAc, 95:5). Removal of the solvent *in vacuo* gave the desired product as a colourless oil (44.3 mg, 45%). A sample of 2,3-dibromobenzofuran was also isolated for characterisation.

Spectroscopic data matched those previously reported.[27,28]

2-bromobenzofuran (4f)
^{1}H NMR (400 MHz, $CDCl_3$) δ 7.52 (dd, *J* = 6.9, 2.1 Hz, 1H), 7.47 (d, *J* = 7.9 Hz, 1H), 7.26 (app pd, *J* = 7.2, 1.3 Hz, 2H), 6.74 (s, 1H); ^{13}C NMR (101 MHz, $CDCl_3$) δ 155.9, 128.8, 128.3, 124.4, 123.5, 120.2, 111.0, 108.4.

2,3-dibromobenzofuran (4f')
^{1}H NMR (500 MHz, $CDCl_3$) δ 7.48 (d, *J* = 7.9 Hz, 1H), 7.45 (d, *J* = 7.9 Hz, 1H), 7.36 – 7.29 (m, 2H); ^{13}C NMR (126 MHz, $CDCl_3$) δ 154.9, 128.7, 128.3, 125.6, 124.2, 119.5, 111.4, 100.1.

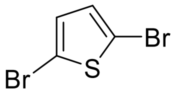

2,5-dibromothiophene (4g)
The general procedure **B** was applied with Bu_4NBr_3 (482.2 mg, 1.0 mmol, 2.0 equiv) and 5-bromothiophene-2-carboxylic acid (103.5 mg, 0.50 mmol, 1.0 equiv) at 100 °C for 16 h. The general work-up procedure gave the desired product as a yellow solid (111.3 mg, 92%).

Spectroscopic data matched those previously reported.[29]

^{1}H NMR (500 MHz, $CDCl_3$) δ 6.84 (s, 2H); ^{13}C NMR (126 MHz, $CDCl_3$) δ 130.5, 111.7.

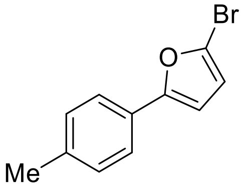

2-bromo-5-(p-tolyl)furan (4h)
The general procedure **B** was applied with Bu_4NBr_3 (241.1 mg, 0.5 mmol, 1.0 equiv) and 5-bromothiophene-2-carboxylic acid (101.1 mg, 0.50 mmol, 1.0 equiv) at 50 °C for 16 h. The general work-up procedure gave the desired product as a yellow solid (105.5 mg, 89%).

^{1}H NMR (500 MHz, $CDCl_3$) δ 7.52 (d, *J* = 8.0 Hz, 2H), 7.19 (d, *J* = 8.0 Hz, 2H), 6.55 (d, *J* = 3.3 Hz, 1H), 6.37 (d, *J* = 3.3 Hz, 1H), 2.37 (s, 3H); ^{13}C NMR (126 MHz, $CDCl_3$) δ 156.3, 137.8, 129.5, 127.4, 123.6, 121.0, 113.4, 106.7, 21.4; IR (ATR) 3021, 2911, 2853, 1513, 1479, 1197, 924, 781; HRMS (APCI) *m/z* calcd. $C_{11}H_{10}OBr$: 236.9910; found $[M+H]^+$ 236.9907.

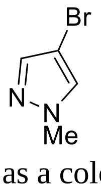

4-bromo-1-methyl-1H-pyrazole (4i)
The general procedure **A** was applied with Bu_4NBr_3 (241.1 mg, 0.50 mmol, 1.0 equiv) and 1-methyl-1H-pyrazole-4-carboxylic acid (63.1 mg, 0.50 mmol, 1.0 equiv) at 100 °C for 16 h. The general work-up procedure gave the desired product as a colourless oil (64.4 mg, 80%).

Spectroscopic data matched those previously reported.[30]

^{1}H NMR (500 MHz, $CDCl_3$) δ 7.43 (s, 1H), 7.37 (s, 1H), 3.89 (s, 3H); ^{13}C NMR (126 MHz, $CDCl_3$) δ 139.9, 130.1, 93.1, 39.6.

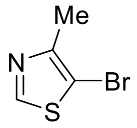

5-bromo-4-methylthiazole (4j)

The general procedure **A** was applied with Bu_4NBr_3 (482.2 mg, 1.0 mmol, 2.0 equiv) and 4-methylthiazole-5-carboxylic acid (71.5 mg, 0.50 mmol, 1.0 equiv) at 50 °C for 16 h. The general work-up procedure gave the desired product as a yellow oil (80.1 mg, 90%).

Spectroscopic data matched those previously reported.[31]

^{1}H NMR (500 MHz, $CDCl_3$) δ 8.68 (s, 1H), 2.44 (s, 3H); ^{13}C NMR (126 MHz, $CDCl_3$) δ 152.6, 152.4, 105.0, 15.7.

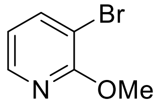

3-bromo-2-methoxypyridine (4k)

The general procedure **A** was applied with Bu_4NBr_3 (482.2 mg, 1.00 mmol, 2.0 equiv), 2-methoxynicotinic acid (76.6 mg, 0.50 mmol, 1.0 equiv) at 100 °C for 16 h. The general work-up procedure was followed then the mixture was dissolved in pentane/EtOAc (5.0 mL, 98:2) and filtered through a short plug of silica with further washings of pentane/EtOAc (4 x 10.0 mL, 98:2). Removal of the solvent *in vacuo* gave the desired product as a colourless oil (82.7 mg, 88%).

Spectroscopic data matched those previously reported.[32]

^{1}H NMR (500 MHz, $CDCl_3$) δ 8.10 (dd, J = 4.9, 1.7 Hz, 1H), 7.79 (dd, J = 7.6, 1.7 Hz, 1H), 6.77 (dd, J = 7.6, 4.9 Hz, 1H), 4.01 (s, 3H); ^{13}C NMR (126 MHz, $CDCl_3$) δ 160.2, 145.6, 141.7, 117.9, 107.2, 54.5.

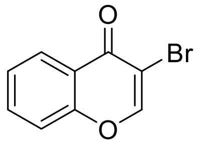

3-bromo-4H-chromen-4-one (4l)

The general procedure **B** was applied with Bu_4NBr_3 (482.2 mg, 1.0 mmol, 2.0 equiv) and chromone-3-carboxylic acid (95.1 mg, 0.50 mmol, 1.0 equiv) at 100 °C for 16 h. The general work-up gave the desired product as a yellow solid (93.4 mg, 83%)

Spectroscopic data matched those previously reported.[33]

^{1}H NMR (400 MHz, $CDCl_3$) δ 8.27 (dd, J = 8.0, 1.6 Hz, 1H), 8.24 (s, 1H), 7.72 (ddd, J = 8.6, 7.2, 1.7 Hz, 1H), 7.52 – 7.40 (m, 2H); ^{13}C NMR (101 MHz $CDCl_3$) δ 172.4, 156.2, 153.9, 134.3, 126.6, 126.0, 123.2, 118.2, 110.8.

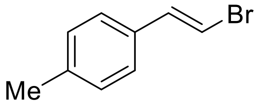

(E)-1-(2-bromovinyl)-4-methylbenzene (4m)

The general procedure **B** was applied with Bu_4NBr_3 (241.1 mg, 0.5 mmol, 1.0 equiv) and (E)-3-(p-tolyl)acrylic acid (81.1 mg, 0.50 mmol, 1.0 equiv) at 50 °C for 3.5 h. The general work-up gave the desired product as a colourless oil (83.8 mg, 85%) as a 3.5:1 mixture with (Z)-1-(2-bromovinyl)-4-methylbenzene.

Spectroscopic data matched those previously reported.[34]

^{1}H NMR (500 MHz, $CDCl_3$) δ 7.20 (d, *J* = 7.6 Hz, 2H), 7.14 (d, *J* = 7.8 Hz, 2H), 7.08 (d, *J* = 14.0 Hz, 1H), 6.71 (d, *J* = 14.0 Hz, 1H), 2.34 (s, 3H); ^{13}C NMR (126 MHz, $CDCl_3$) δ 138.4, 137.2, 133.3, 129.6, 126.3, 105.6, 21.4.

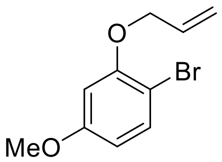

2-(allyloxy)-1-bromo-4-methoxybenzene (2A)

The general procedure **B** was applied with Bu_4NBr_3 (241.1 mg, 1.0 mmol, 1.0 equiv), 2-(allyloxy)-4-methoxybenzoic acid (104.1 mg, 0.50 mmol, 1.0 equiv) at 50 °C for 30 min. The general work-up procedure was followed then the mixture was purified by column chromatography (Hexane 100%). Removal of the solvent *in vacuo* gave the desired product as a colourless oil (73.0 mg, 60%).

^{1}H NMR (400 MHz, $CDCl_3$) δ 7.41 (d, *J* = 8.7 Hz, 1H), 6.48 (d, *J* = 2.7 Hz, 1H), 6.40 (dd, *J* = 8.7, 2.7 Hz, 1H), 6.06 (ddt, *J* = 17.2, 10.5, 5.0 Hz, 1H), 5.49 (dd, *J* = 17.2, 1.5 Hz, 1H), 5.31 (dd, *J* = 10.5, 1.5 Hz, 1H), 4.58 (d, *J* = 5.0 Hz, 2H), 3.78 (s, 3H); ^{13}C NMR (101 MHz, $CDCl_3$) δ 159.8, 155.4, 133.0, 132.3, 117.6, 106.1, 102.8, 101.2, 69.5, 55.4; IR (ATR) 3021, 2936, 1731, 1580, 1442, 1305, 1202, 1168, 1023, 628; HRMS (EI) *m/z* calcd. $C_{10}H_{11}O_2Br$: 241.9937; found $[M]^+$ 241.9937.

Procedure for Multi-Gram Scale Synthesis of 2-Bromo-1,3-dimethoxybenzene (2e)

On the benchtop a 1 L pear-shaped flask was charged with 2,6-dimethoxybenzoic acid (5.01 g, 27.5 mmol, 1.0 equiv), anhydrous K_3PO_4 (5.84 g, 27.5 mmol, 1.0 equiv), Bu_4NBr_3 (13.26 g, 27.5 mmol, 1.0 equiv) and MeCN (137.5 mL, 0.2 M). The mixture was stirred at room temperature (~18 °C) for 16 h. After this time H_2O (400 mL) was added and the reaction triturated with $Na_2S_2O_3$ (30.0 g). The mixture was transferred to a 1 L separating funnel and sat. Na_2CO_3 (aq, 100 mL) and pentane (250 mL) were added. The organic layer was collected and the aqueous layer was further washed with pentane (3 x 100 mL). The organic fractions were dried with $MgSO_4$ (30.0 g) and concentrated *in vacuo* to yield the desired product as a white solid (5.49 g, 92%).

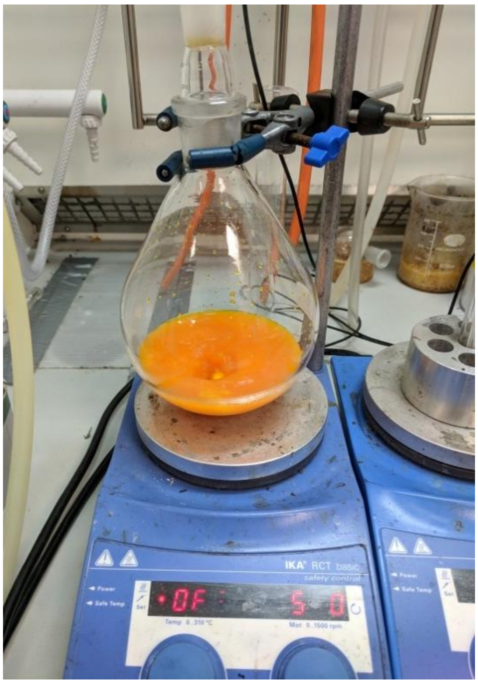

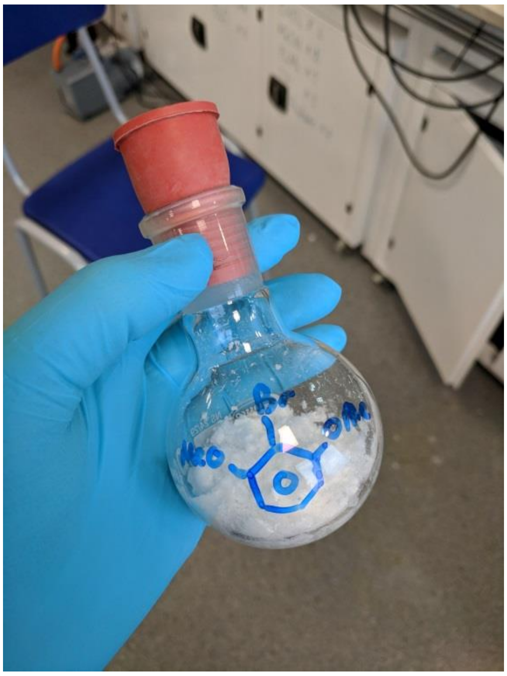

Computed reaction path

All quantum chemical calculations were performed with the Gaussian 09 d.01program package.[35] Geometries were optimized using DFT with the B3LYP functional and the 6-31+G(d) basis set in gas phase without symmetry constraints. Solvent correction for acetonitrile according to the SMD model and dispersion correction according to the DFT-D3 scheme with BJ dampening were applied as single point energies at the B3LYP/6-311+G(d) level. The stationary points were characterised as minima (no negative frequencies) or transition states (one negative frequency > 30 cm^{-1}) through frequency analysis. To verify that the located transition states connected the desired minima, the reaction paths were followed in both directions through IRC calculations.

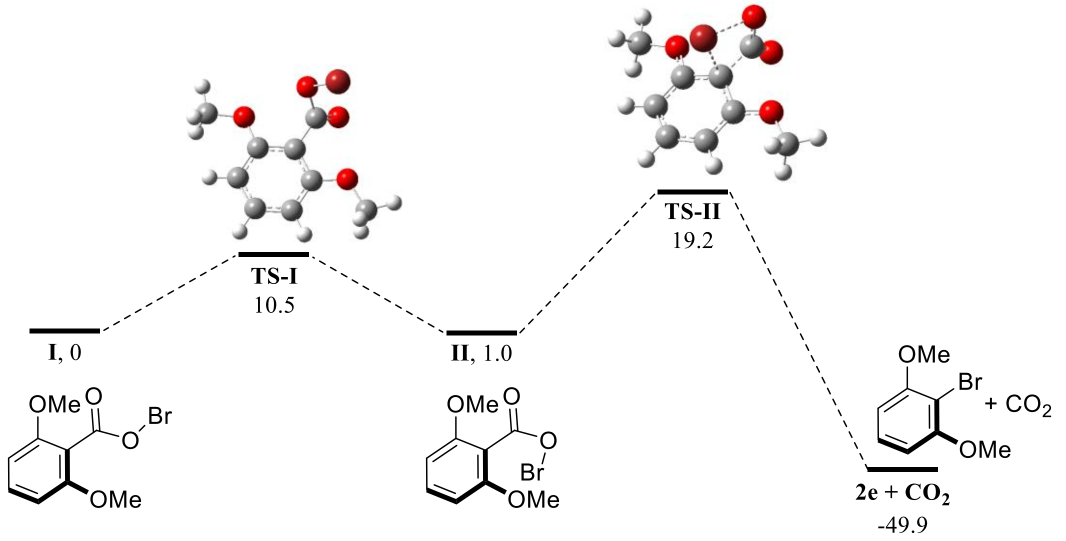

Scheme S1. Proposed reaction mechanism. **TS-I:** rotation transition state, **TS-II:** decarboxylative bromination transition state.

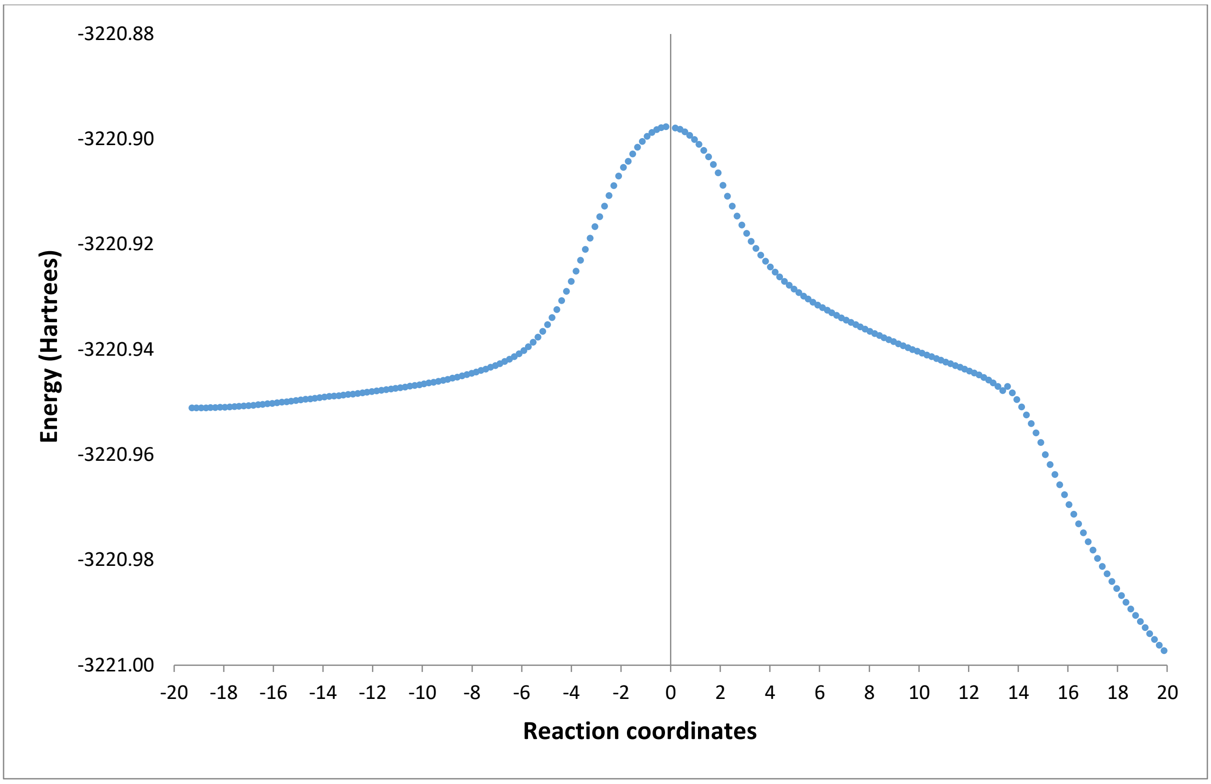

Scheme S2. Energy along the path from **II** (-19) to **TS-II** (0) and then to **2e** (20).

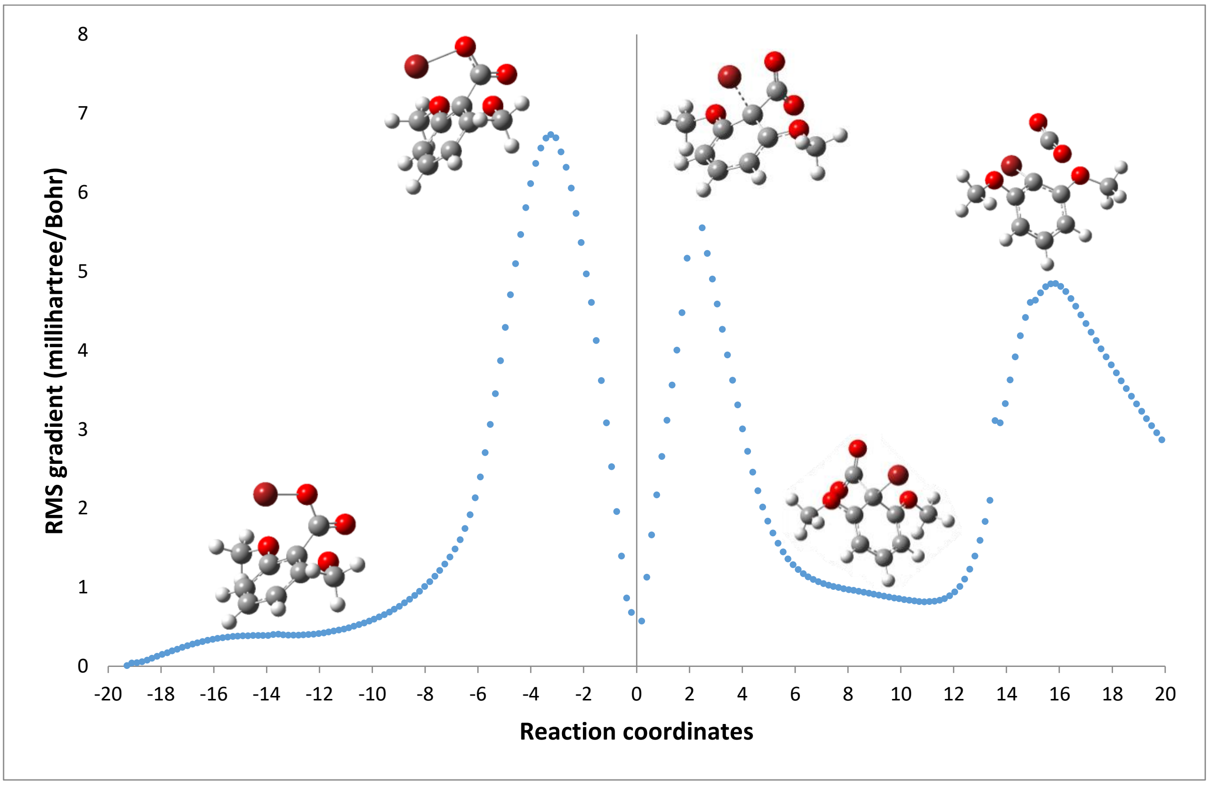

Scheme S3. RMS (root mean square) of the gradient along the path from **II** (-19.3) to **TS-II** (0) and then to **2e** (20). Representative structures along the path are shown.

Determination of ^{13}C-KIEs at natural abundance

The intermolecular competitive ^{13}C-KIEs were measured as previously reported, according to the following formulas.[36,37]

$$\mathrm{KIE} = \frac{\log(1-F)}{\log[(1-F)\mathrm{R/R_0}]}$$

$$\Delta\mathrm{KIE_F} = \Delta\mathrm{F}\,\frac{\partial\mathrm{KIE}}{\partial\mathrm{F}} = \frac{-\ln(\mathrm{R/R_0})\ \Delta\mathrm{F}}{(1-F)\ln^2[(1-F)\mathrm{R/R_0}]}$$

$$\Delta\mathrm{KIE_R} = \Delta(\mathrm{R/R_0})\frac{\partial\mathrm{KIE}}{\partial(\mathrm{R/R_0})} = \frac{-\ln(1-\mathrm{F})\ \Delta\mathrm{R/R_0}}{(\mathrm{R/R_0})\ln^2[(1-\mathrm{F})\mathrm{R/R_0}]}$$

$$\Delta\mathrm{KIE} = \sqrt{\Delta\mathrm{KIE_F}^{\,2} + \Delta\mathrm{KIE_R}^{2}}$$

F: starting material conversion

R/R_0: proportion of the minor isotopic component in recovered material compared to the original starting material, is the ratio between the area of the peaks of the starting material and the area of the area of the peaks of the starting material recovered after the reaction.

ΔKIE_F: uncertainty related to the measurement of the conversion

ΔKIE_R: uncertainty related to the quantitative ^{13}C NMR.

Experimental procedure

A 150 mL round-bottom flask equipped with a magnetic stirring bar was charged with Bu_4NBr_3 (7.715 g, 16 mmol, 1.0 equiv), 2,6-dimethoxybenzoic acid (2.915 g, 16 mmol, 1.0 equiv), anhydrous K_3PO_4 (3.396 g, 16 mmol, 1.0 equiv) and acetonitrile (80.0 mL, 0.2 M). The flask was sealed with a septum, equipped with a balloon, placed in an oil bath pre-heated at 30 °C and stirred for 70 minutes. After this time the reaction was quenched with 10% aq. $Na_2S_2O_3$ (8.0 mL), acidified with trifluoroacetic acid (6.2 mL, 80 mmol, 16 equiv) and dried over Na_2SO_4. The mixture was filtered through cotton in a 200 mL volumetric flask with acetonitrile. Six 1 mL aliquots were taken, 1 mL of a 0.08 M solution of 4-nitrotoluene in CD_3CN was added as internal standard and the samples were analysed by 1H-NMR to determine the conversion. To ensure complete relaxation the T1 was measured for each mixture. Spectra were recorded at 500 MHz using 40 s delays between calibrated $\pi/2$ pulses, 6.5 s acquisition time and 16 scans. A small amount of 2,6-dimethoxy-3-bromobenzoic acid (< 6%) was detected by NMR and was not considered in determining the conversion due to the small amount and its influence on only one of the two carbons used. The remaining crude was evaporated to dryness and the unreacted starting material was recovered by column chromathography on silica gel (hexane/Et_2O/THF, 7:1:2 with 2% AcOH). The fractions that contained no impurities (as judged by GC-MS analysis) were reunited and concentrated under reduced pressure. Pentane was added to the residual solution, the obtained solid was filtered, washed with pentane and dried *in vacuo*.

Quantitative ^{13}C NMR

The NMR samples were prepared using 2,6-dimethoxybenzoic acid (89 mg) in 0.5 mL of $(CD_3)_2SO$. To ensure complete relaxation the T1 was measured for each sample. The ^{13}C spectra were recorded at 125.81 MHz using inverse gated decoupling, 64 s delays between calibrated π/2 pulses, 1.4 s acquisition time and 32 scans collecting 32768 points, the FID was then zero-filled to 128K. Six spectra were recorded for each sample, a zeroth order baseline correction was generally applied, but in no case a first or higher correction was tested. Integrations were numerically determined using a constant region for each peak corresponding to a multiple of the peak widths at half height ($\pm 5w_{1/2}$) and referred to the area of the peak of C-4 and C-6, which was assigned a value of 2000.

C	fid1	fid2	fid3	fid4	fid5	fid6	AV	SD
C1	1038.90	1047.23	1043.72	1044.16	1046.78	1054.00	1045.80	5.00
C2	1025.09	1033.50	1036.58	1034.52	1035.82	1034.88	1033.40	4.21
C3	2051.09	2055.62	2059.13	2061.75	2056.55	2059.13	2057.21	3.70
C4	2000.00	2000.00	2000.00	2000.00	2000.00	2000.00	2000.00	0.00
C5	984.29	991.35	984.87	992.92	985.29	988.54	987.88	3.65

Table S1. ^{13}C NMR integrations of 2,6-dimethoxybenzoic acid starting material (AV= average, SD= standard deviation).

Experiment 1

1	2	3	4	5	6	AV	SD
75.32	74.96	75.61	75.60	74.92	76.17	75.43	0.47

Table S2. Conversion in experiment 1 (AV= average, SD= standard deviation).

C	fid1	fid2	fid3	fid4	fid5	fid6	AV	SD
C1	1064.96	1062.54	1067.76	1062.89	1067.70	1069.18	1065.84	2.78
C2	1072.49	1072.46	1075.28	1065.36	1077.87	1077.40	1073.48	4.60
C3	2046.35	2041.94	2042.25	2042.46	2049.65	2054.81	2046.24	5.17
C4	2000.00	2000.00	2000.00	2000.00	2000.00	2000.00	2000.00	0.00
C5	986.48	989.65	983.33	985.20	991.99	998.66	989.22	5.58

Table S3. ^{13}C NMR integrations of 2,6-dimethoxybenzoic acid starting material recovered from experiment 1 (AV= average, SD= standard deviation).

C	KIE	ΔKIE	R/R0	Δ(R/R0)	$ΔKIE_F$	$ΔKIE_R$
C1	1.014	0.004	1.019	0.006	< 0.001	0.004
C2	1.028	0.004	1.039	0.006	< 0.001	0.004
C3	0.996	0.002	0.995	0.003	< 0.001	0.002
C4	1.000	0.000	1.000	0.000	0.000	0.000
C5	1.001	0.005	1.001	0.007	< 0.001	0.005

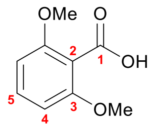

Table S4. Determination of ^{13}C NMR KIEs for experiment 1.

Experiment 2

1	2	3	4	5	6	AV	SD
64.38	64.38	64.85	65.04	65.00	65.05	64.78	0.32

Table S5. Conversion in experiment 2 (AV= average, SD= standard deviation).

C	fid1	fid2	fid3	fid4	fid5	fid6	AV	SD
C1	1052.18	1059.46	1055.75	1061.47	1052.31	1049.33	1055.08	4.68
C2	1058.66	1067.16	1057.56	1057.63	1060.38	1057.48	1059.81	3.77
C3	2036.93	2039.78	2039.08	2043.07	2045.11	2038.88	2040.48	3.02
C4	2000.00	2000.00	2000.00	2000.00	2000.00	2000.00	2000.00	0.00
C5	982.96	985.00	988.62	984.13	984.19	982.41	984.55	2.20

Table S6. ^{13}C NMR integrations of 2,6-dimethoxybenzoic acid starting material recovered from experiment 2 (AV= average, SD= standard deviation).

C	KIE	ΔKIE	R/R0	Δ(R/R0)	$ΔKIE_F$	$ΔKIE_R$
C1	1.009	0.006	1.009	0.007	< 0.001	0.006
C2	1.025	0.005	1.026	0.006	< 0.001	0.005
C3	0.992	0.002	0.992	0.002	< 0.001	0.002
C4	1.000	0.000	1.000	0.000	0.000	0.000
C5	0.997	0.004	0.997	0.004	< 0.001	0.004

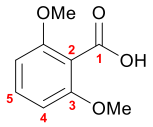

Table S7. Determination of ^{13}C NMR KIEs for experiment 2.

Calculated ^{13}C isotope effects for the proposed pathway

Kinetic isotope effects (KIEs) and equilibrium isotope effects (EIEs) were calculated according to the Bigelesein equation at 303.15 K using ISOEFF98. Frequencies were obtained at the B3LYP/6-31+G(d) level in gas phase,[38] the scaling factor of 0.9614 was applied.[39] The tunnelling correction on KIE-I was evaluated under equal conditions using Pyquiver according to Bell's infinite-parabola model.

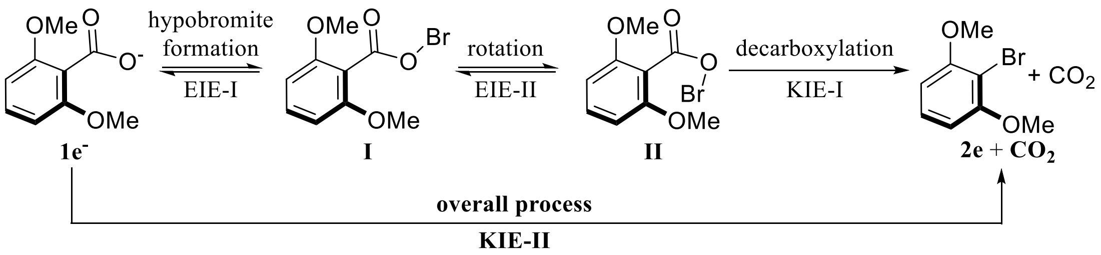

Scheme S4. EIEs and KIEs associated with the proposed pathway.

C	EIE-I[a]	EIE-II[a]	KIE-I[a]	KIE-II[a]	Exp-1[b]	Exp-2[b]
C1	0.999	1.000	1.013	1.013	1.014(4)	1.009(6)
C2	0.992	0.998	1.033	1.023	1.028(4)	1.025(5)
C3	0.995	1.001	1.002	0.997	0.996(2)	0.992(2)
C4	0.998	1.000	1.001	1.000	1.000*	1.000*
C5	0.997	1.000	1.004	1.001	1.001(5)	0.997(4)

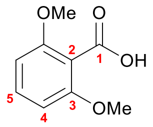

Table S8. Comparison of computed and experimental isotope effects. [a] Computed isotope effects, without approximation to the third decimal EIE-I×EIE-II×KIE-I = KIE-II. [b] Experimental values, for further information vide supra.

C	KIE-I[a]	KIE-I_t[b]	Δ_t[c]
C1	1.013	1.014	0.001
C2	1.033	1.036	0.003
C3	1.002	1.002	0.000
C4	1.001	1.001	0.000
C5	1.004	1.004	0.000

Table S9. Evaluation of tunnelling. [a] KIE-I uncorrected values obtained with Pyquiver. [b] KIE-I values corrected for tunnelling. [c] KIE-I_t – KIE-I.

Coordinates and energies for the computed structures

I (minimum)

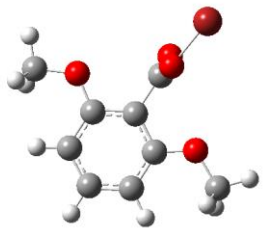

No imaginary frequencies
E = -3220.95463851 a.u.
G = -3220.828139 a.u.
Solvent correction = -0.020197 a.u.
Dispersion correction = -0.044230 a.u.

0 1

C	-2.65052400	1.65085500	-0.24191300
C	-3.15639100	-0.73600900	-0.29988000
C	-3.55225100	0.59819000	-0.40379300
C	-1.81332600	-1.02551500	-0.02121400
C	-1.30689800	1.35715600	0.02883200
C	-0.89072000	0.02006000	0.15051300
Br	3.15341000	-0.24119000	-0.25988300
H	-2.99328200	2.67489500	-0.32667000
H	-3.88234100	-1.52839900	-0.43585900
H	-4.59373500	0.82400100	-0.61747900
C	0.52807500	-0.28484900	0.53197900
O	0.91648700	-0.64765700	1.61180500
O	1.31283500	-0.06921800	-0.56217900
C	-0.68780100	3.66997300	0.17213800
H	-1.42455900	3.91886700	0.94609100
H	0.23874200	4.21362800	0.36155400
H	-1.07756800	3.94401800	-0.81609200
O	-0.33735200	2.29102600	0.22241300
O	-1.30193800	-2.27922000	0.09178000
C	-2.17420300	-3.39669000	-0.03775300
H	-1.54472900	-4.27568600	0.10724500
H	-2.95877500	-3.37685600	0.72877900
H	-2.62913300	-3.43130700	-1.03568700

TS-I (transition state)

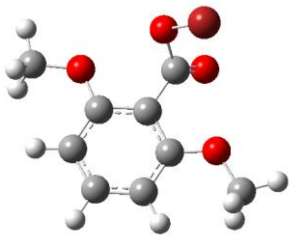

1 imaginary frequency = -72.30 cm-1
E = -3220.93685027 a.u.
G = -3220.810152 a.u.
Solvent correction = -0.020266 a.u.
Dispersion correction = -0.045357 a.u.

0 1

C	-2.25215500	1.72200000	-0.56279100
C	-2.84517300	-0.64326400	-0.58845500
C	-3.13542700	0.68998000	-0.87943500

C	-1.62349100	-0.95198300	0.02459100
C	-1.03694400	1.41217900	0.06495900
C	-0.71417700	0.07129000	0.35504700
Br	2.63207900	-0.32592000	-0.64276000
H	-2.50804000	2.74574600	-0.80691600
H	-3.55939700	-1.41956100	-0.83427800
H	-4.07959000	0.93224300	-1.36039600
C	0.55629300	-0.24921200	1.06505000
O	0.91327500	0.20119000	2.11741400
O	1.35232400	-1.20830900	0.38924000
C	-0.36571600	3.71142200	0.17679400
H	-1.25467300	4.03757800	0.73096500
H	0.51148800	4.24185400	0.54987300
H	-0.49524300	3.92299700	-0.89222100
O	-0.09786000	2.33224900	0.40154300
O	-1.25075300	-2.20415000	0.39793000
C	-2.10741200	-3.30188100	0.10236800
H	-1.58490200	-4.18567300	0.47080200
H	-3.07038300	-3.20224500	0.61897200
H	-2.27271900	-3.39676000	-0.97799300

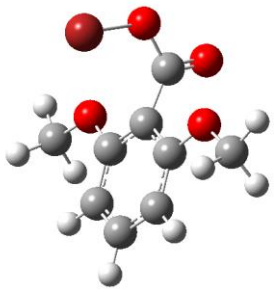

II (minimum)

No imaginary frequencies
E = -3220.95096065 a.u.
G = -3220.823738 a.u.
Solvent correction = -0.020542 a.u.
Dispersion correction = -0.046729 a.u.

0 1

C	-2.33970700	1.22091000	-0.68274100
C	-2.34132700	-1.21779500	-0.68284900
C	-2.91798100	0.00195700	-1.03959500
C	-1.14309900	-1.21286300	0.04327700
C	-1.14148100	1.21432500	0.04337600
C	-0.53566400	0.00031300	0.39941000
Br	2.19331400	-0.00135700	-0.90088800
H	-2.81505600	2.15107400	-0.96972100
H	-2.81789600	-2.14730300	-0.96993000
H	-3.84715300	0.00260300	-1.60335700
C	0.58972600	-0.00048700	1.38845800
O	0.45473900	-0.00042200	2.58498000
O	1.88997700	-0.00128800	0.94804600
C	-1.03707600	3.60830200	0.17616200
H	-2.02157800	3.71408300	0.64851700
H	-0.34437600	4.33154000	0.60853800
H	-1.12143100	3.78151100	-0.90402900

O	-0.47358500	2.32944300	0.44860400
O	-0.47668200	-2.32890100	0.44841600
C	-1.04188000	-3.60699300	0.17591200
H	-0.35016500	-4.33117500	0.60828600
H	-2.02653600	-3.71147100	0.64823300
H	-1.12643200	-3.78005300	-0.90428800

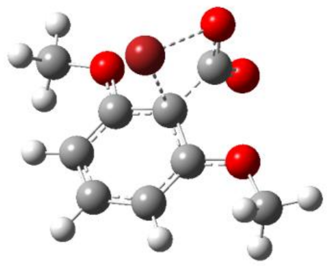

TS-II (transition state)

1 imaginary frequency = -366.75 cm-1
E = -3220.89764236 a.u.
G = -3220.773339 a.u.
Solvent correction = -0.039540 a.u.
Dispersion correction = -0.049100 a.u.

0 1

C	1.23398900	1.99108600	-0.48271300
C	-1.23397200	1.99110000	-0.48270000
C	0.00000900	2.52914300	-0.87432000
C	-1.23802700	0.82713900	0.27687700
C	1.23804000	0.82712500	0.27686200
C	0.00000400	0.11176300	0.50175200
Br	-0.00001000	-1.26574400	-1.44544700
H	2.15316500	2.48939000	-0.76514900
H	-2.15314600	2.48941400	-0.76512700
H	0.00001100	3.43022200	-1.48229000
C	-0.00000200	-1.11030400	1.44354900
O	0.00002200	-0.94679400	2.66159500
O	-0.00003300	-2.22345200	0.80197800
C	3.61138700	0.77526800	0.53898800
H	3.68090400	1.79078700	0.94492100
H	4.30645100	0.11795600	1.06058700
H	3.83020300	0.77751300	-0.53471600
O	2.31458100	0.22346900	0.78307000
O	-2.31457000	0.22350300	0.78310500
C	-3.61137700	0.77528600	0.53898700
H	-4.30644600	0.11796900	1.06057100
H	-3.68091500	1.79080700	0.94491300
H	-3.83016500	0.77752400	-0.53472300

2e (minimum)

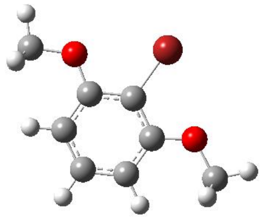

No imaginary frequencies
E = -3032.437746 a.u.
G = -3032.317164 a.u.
Solvent correction = -0.015101 a.u.
Dispersion correction = -0.038091 a.u.

0 1

C	0.00006400	-0.03033100	0.00000100
C	1.22000000	0.66432300	0.00000000
C	-1.21981600	0.66457700	0.00000000
C	1.21657600	2.06779200	0.00000000
C	-1.21605200	2.06799700	0.00000000
C	0.00035200	2.74975600	0.00000000
H	-2.14640300	2.62337600	0.00000000
H	0.00034100	3.83644800	0.00000000
O	-2.34641200	-0.09526600	0.00000000
C	-3.61271700	0.55036800	-0.00000100
H	-3.74563800	1.16832400	-0.89731900
H	-4.34963600	-0.25435100	-0.00000100
H	-3.74563900	1.16832400	0.89731800
H	2.14710300	2.62287600	0.00000000
Br	-0.00030500	-1.92633700	0.00000000
O	2.34654200	-0.09572100	0.00000000
C	3.61292900	0.54967600	-0.00000100
H	3.74596400	1.16753400	0.89738300
H	4.34957900	-0.25532300	-0.00000100
H	3.74596300	1.16753300	-0.89738600

CO_2 (minimum)

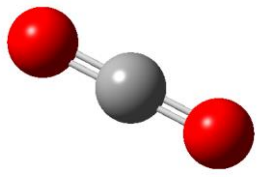

No imaginary frequencies
E = -188.590392621 a.u.
G = -188.599542 a.u.
Solvent correction = −0.000505 a.u.
Dispersion correction = −0.001642 a.u.

0 1

C	0.00000000	0.00000000	0.00000000
O	0.00000000	0.00000000	1.16935600
O	0.00000000	0.00000000	-1.16935600

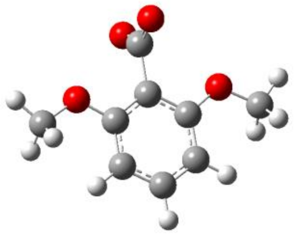

1e^{-} (minimum)

No imaginary frequencies
E = -649.3289882 a.u.
G = -649.202517 a.u.

-1 1

C	2.59626000	1.17262500	-0.31226600
C	2.59699000	-1.17142100	0.31192900
C	3.28905800	0.00079800	-0.00024600
C	1.19185900	-1.15751800	0.30825200
C	1.19113800	1.15793100	-0.30826700
C	0.46029600	0.00000000	0.00008100
H	1.72711300	-3.83579100	0.11387100
H	3.14992200	2.07467300	-0.55151900
H	3.15121500	-2.07315800	0.55104500
H	4.37757800	0.00110400	-0.00037200
C	-1.09370300	-0.00043200	0.00024500
O	-1.61648400	0.28876200	1.10278900
O	-1.61654900	-0.29011500	-1.10214000
C	1.09384800	3.46782900	-0.93826500
H	0.30360500	4.19711400	-1.13658800
H	1.72495400	3.83651200	-0.11410600
H	1.71687200	3.35784100	-1.83997800
O	0.43613900	2.27010500	-0.60345200
O	0.43754400	-2.27012000	0.60358700
C	1.09598400	-3.46750200	0.93818800
H	1.71914100	-3.35723300	1.83977500
H	0.30618400	-4.19723200	1.13663800

NMR Spectra

2-bromoanisole (2a)

^{1}H NMR ($CDCl_3$)

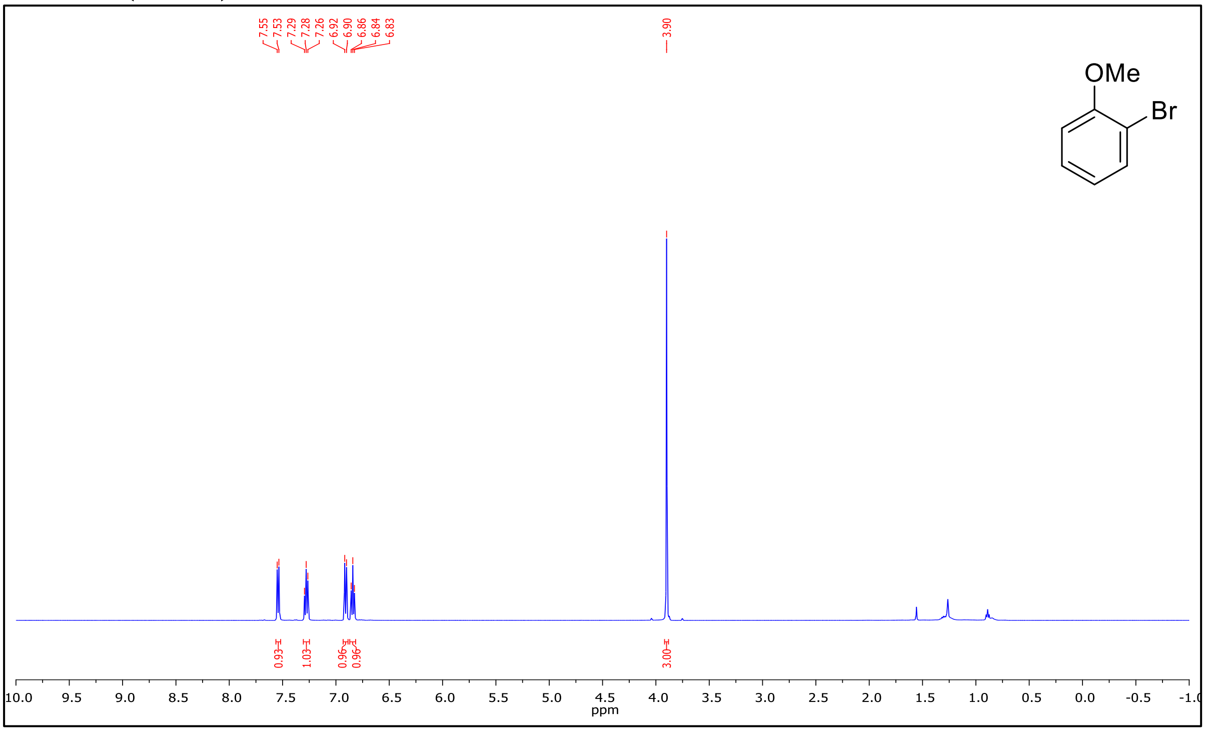

^{13}C NMR ($CDCl_3$)

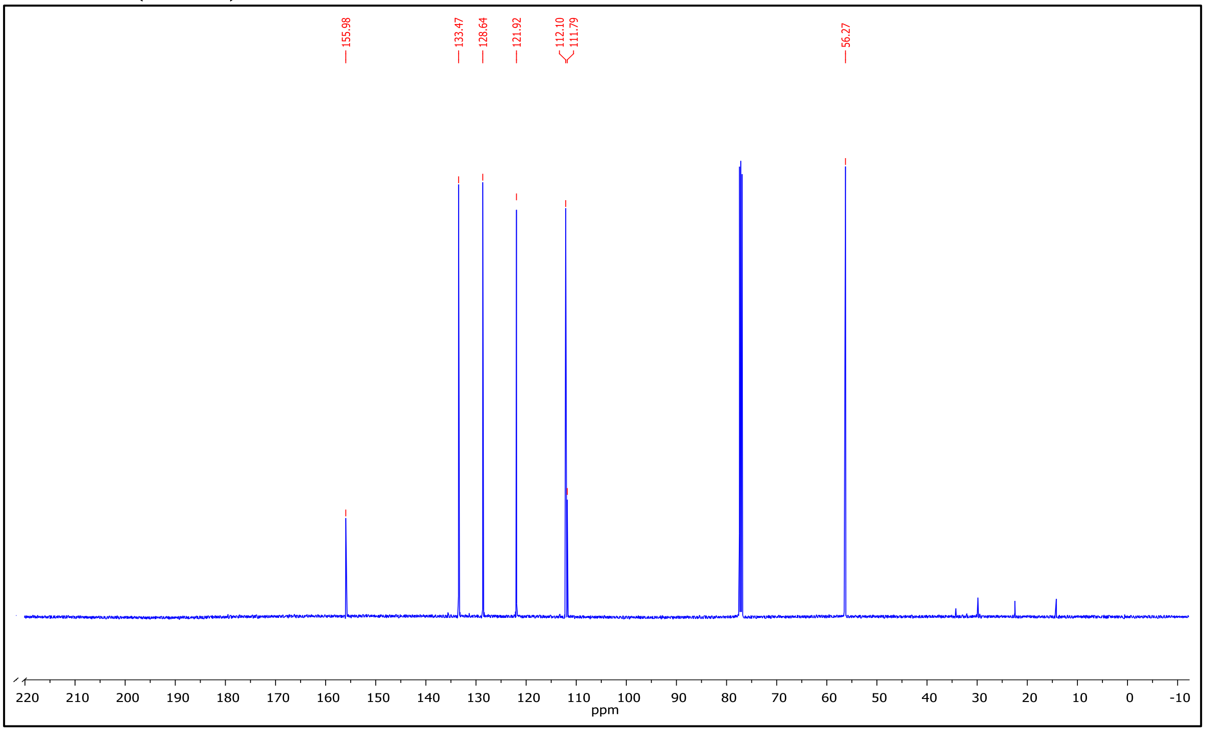

4-bromoanisole (2b)

^{1}H NMR ($CDCl_3$)

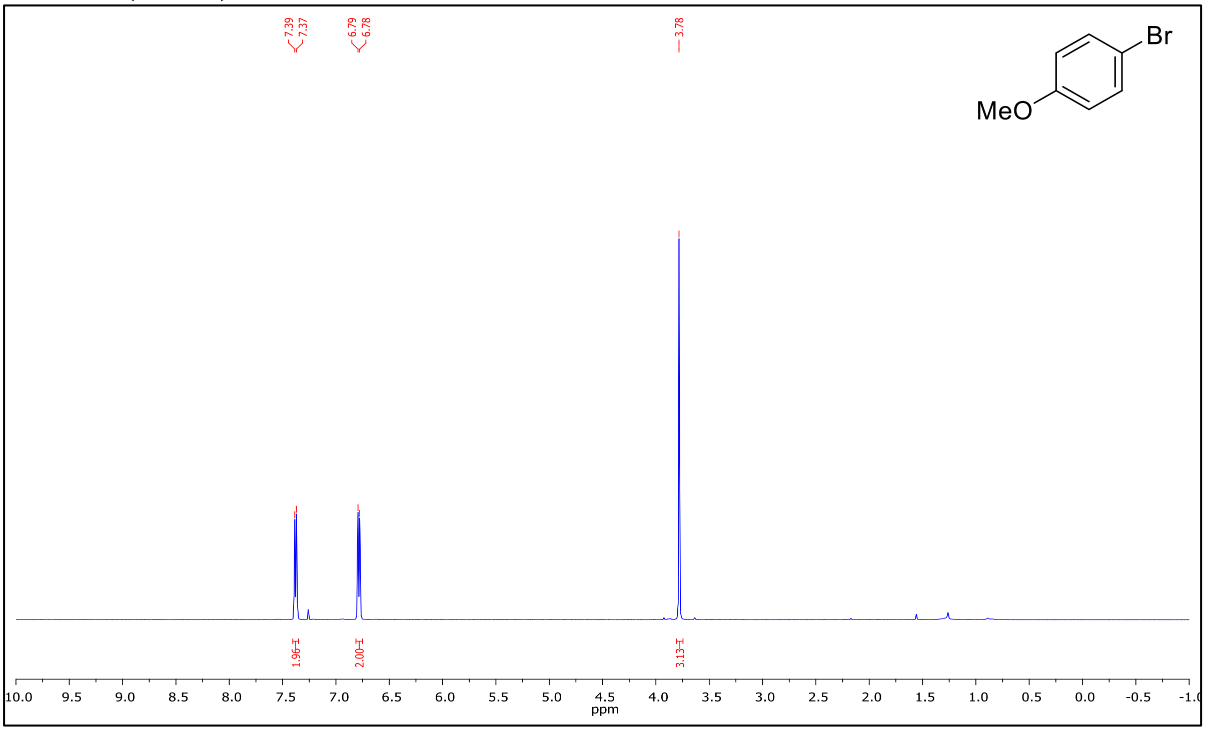

^{13}C NMR ($CDCl_3$)

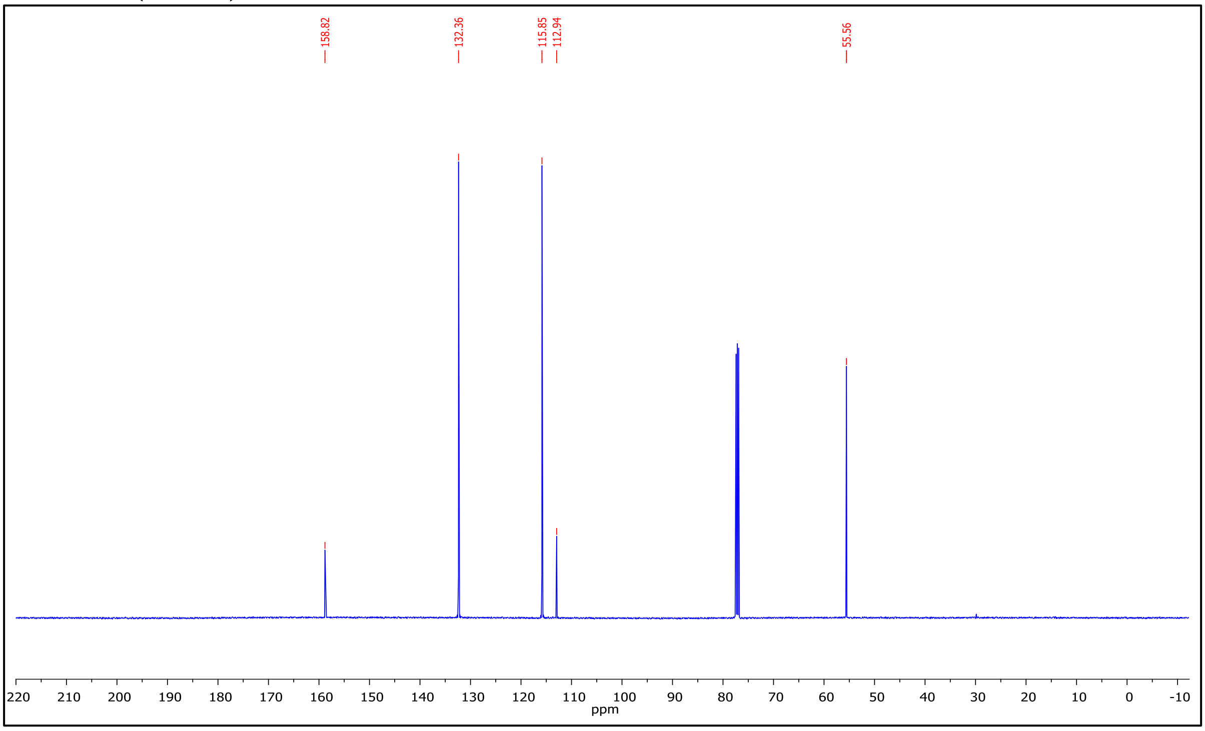

1-bromo-2,4-dimethoxybenzene (2d)

^{1}H NMR ($CDCl_3$)

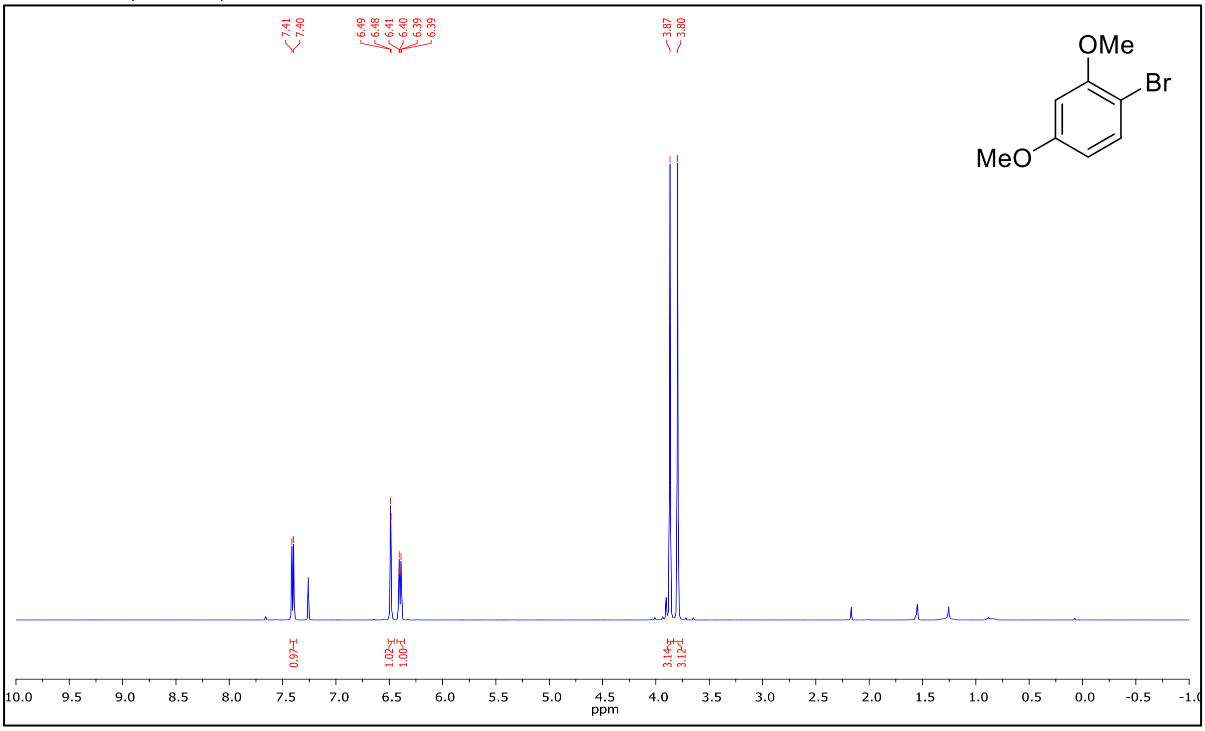

^{13}C NMR ($CDCl_3$)

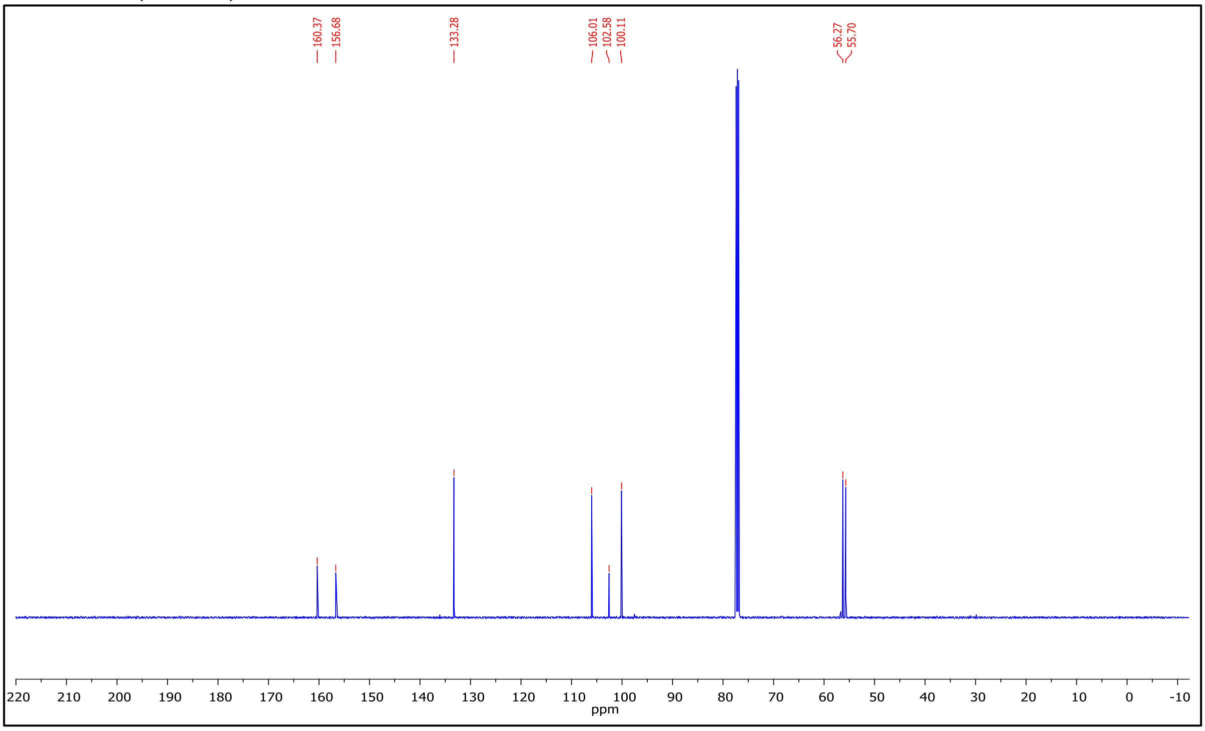

2-bromo-1,3-dimethoxybenzene (2e)

^{1}H NMR ($CDCl_3$)

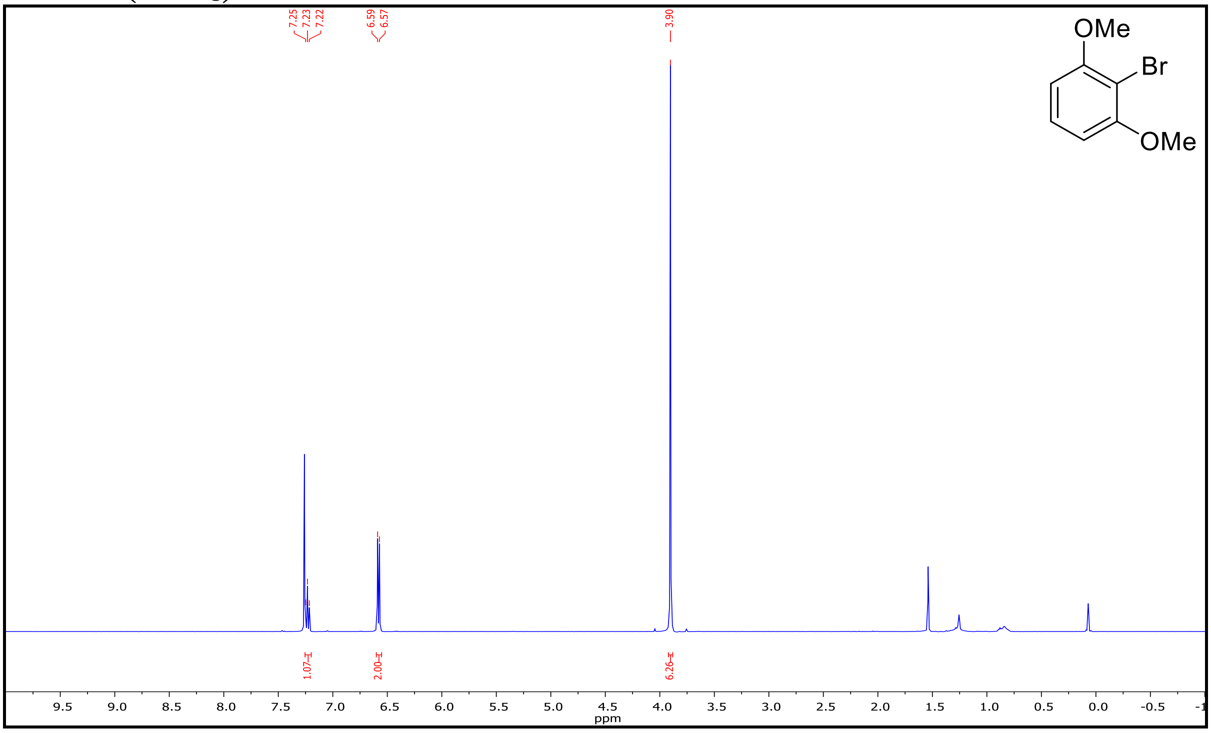

^{13}C NMR ($CDCl_3$)

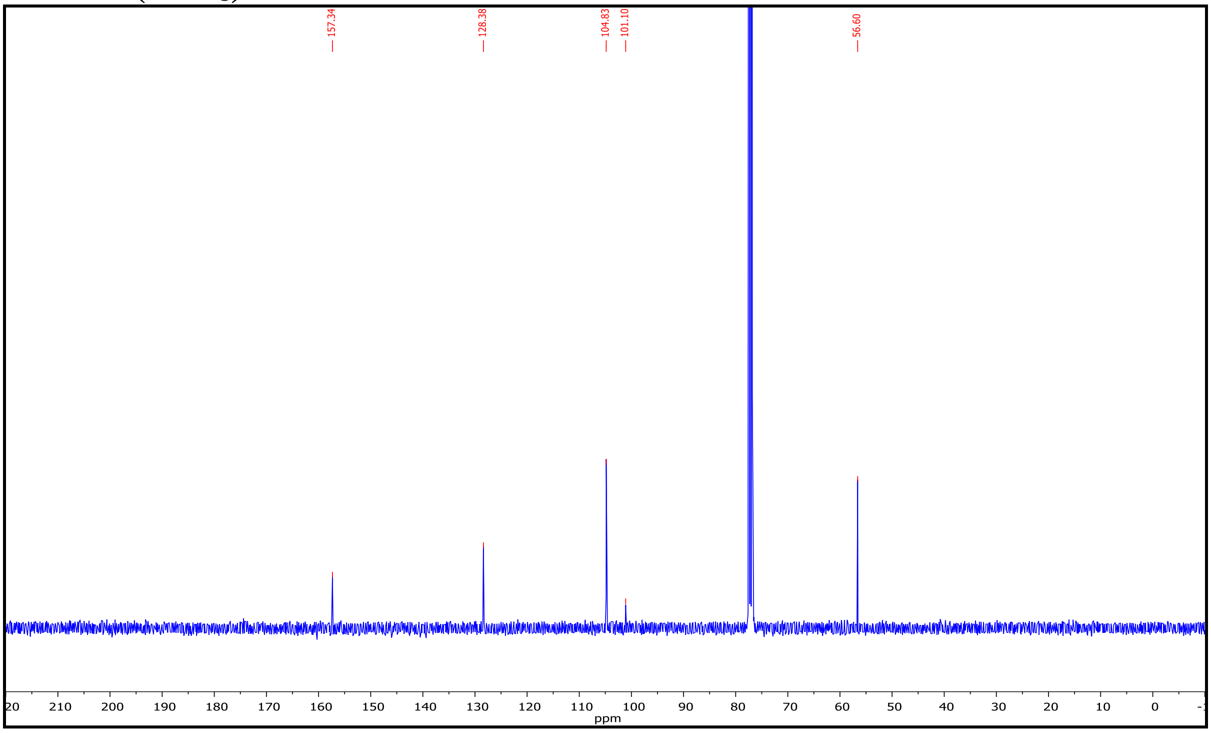

1-bromo-2-methoxy-4-methylbenzene (2f)

^{1}H NMR ($CDCl_3$)

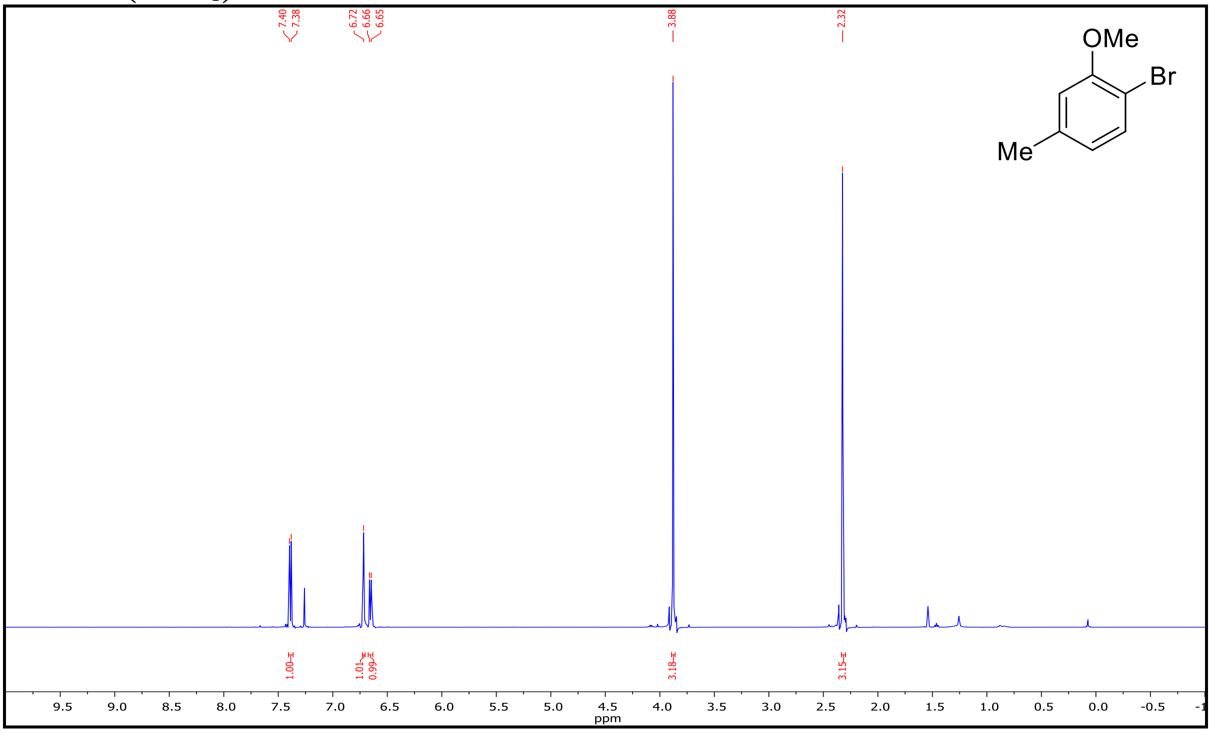

^{13}C NMR ($CDCl_3$)

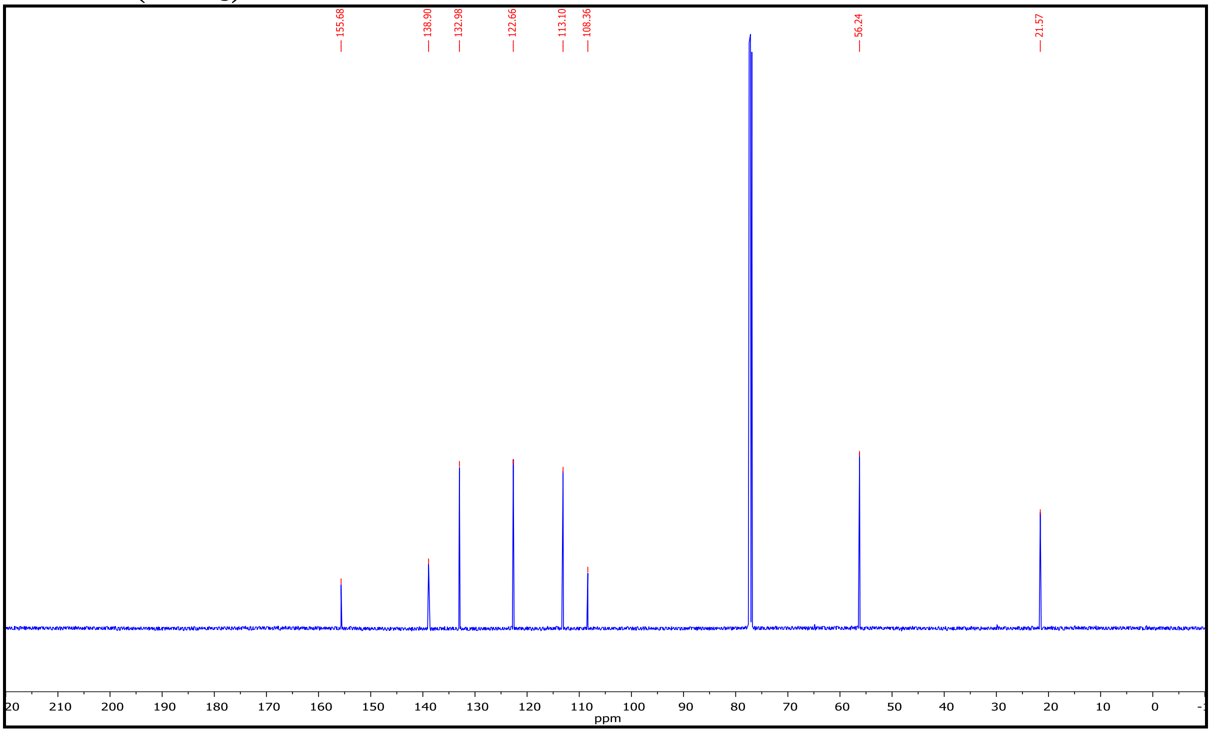

1-bromo-4-methoxy-2-methylbenzene (2g)

^{1}H NMR ($CDCl_3$)

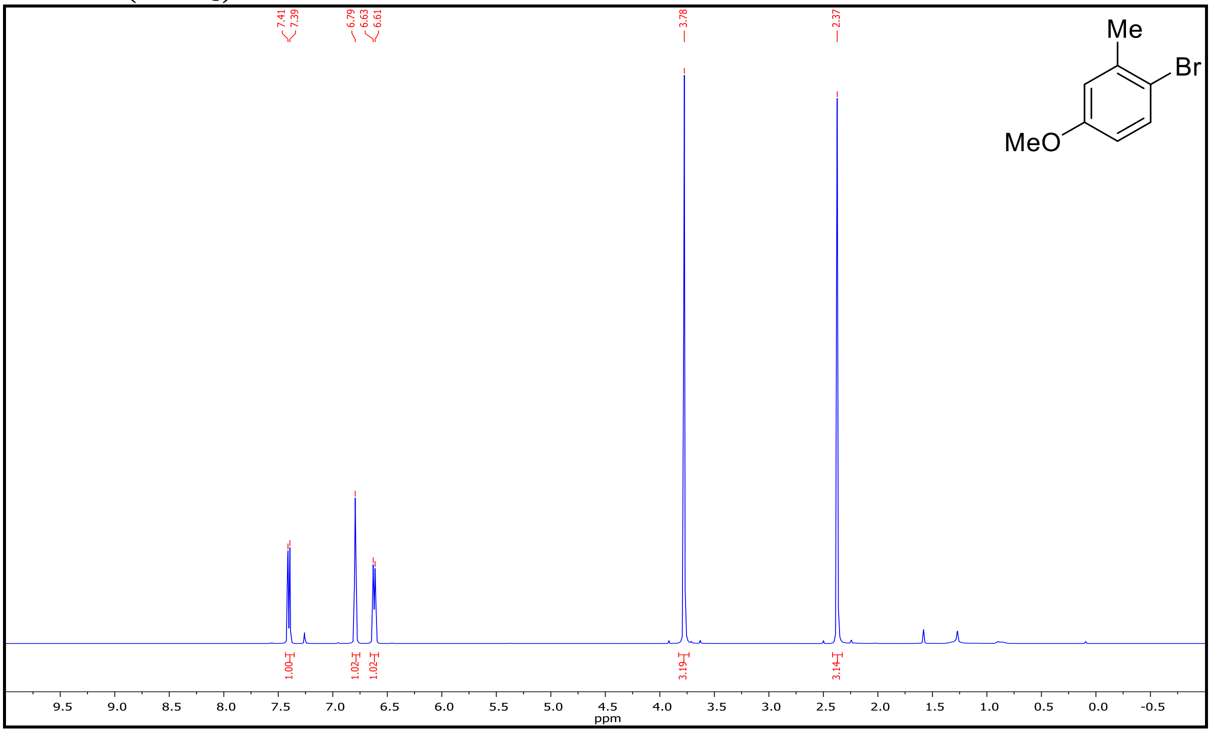

^{13}C NMR ($CDCl_3$)

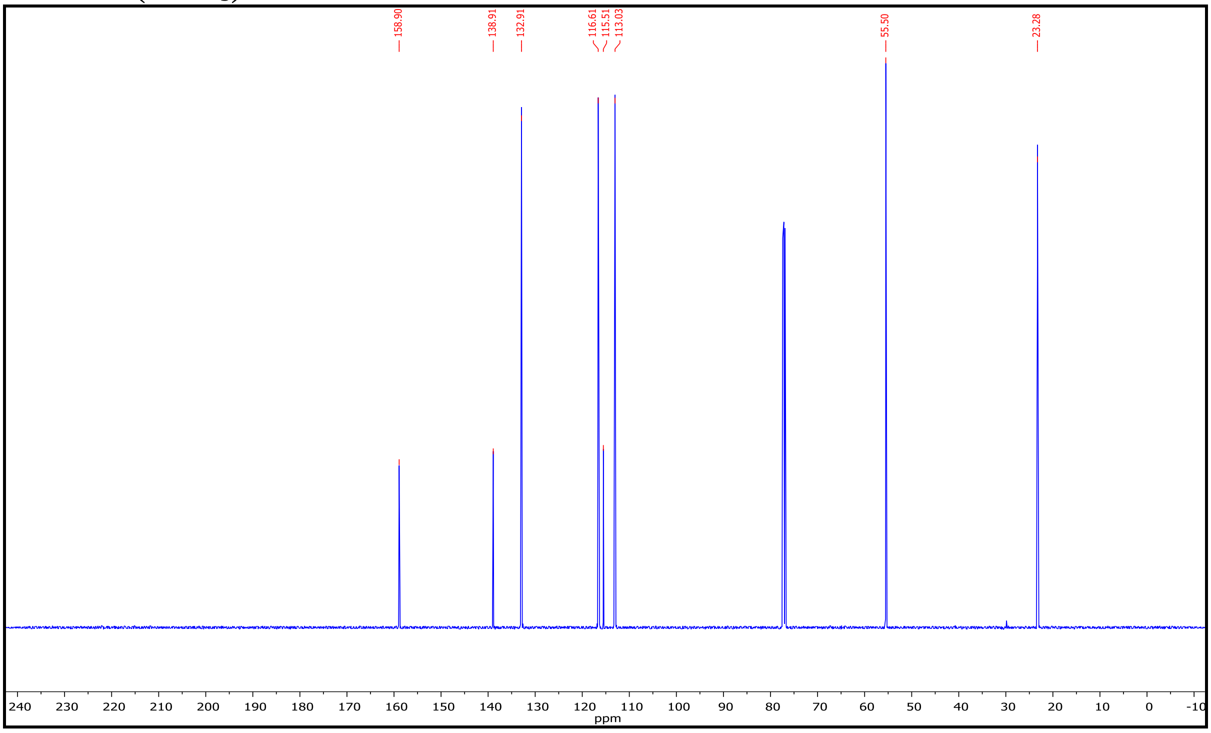

4-bromo-1-methoxy-2-methylbenzene (2h)

^{1}H NMR ($CDCl_3$)

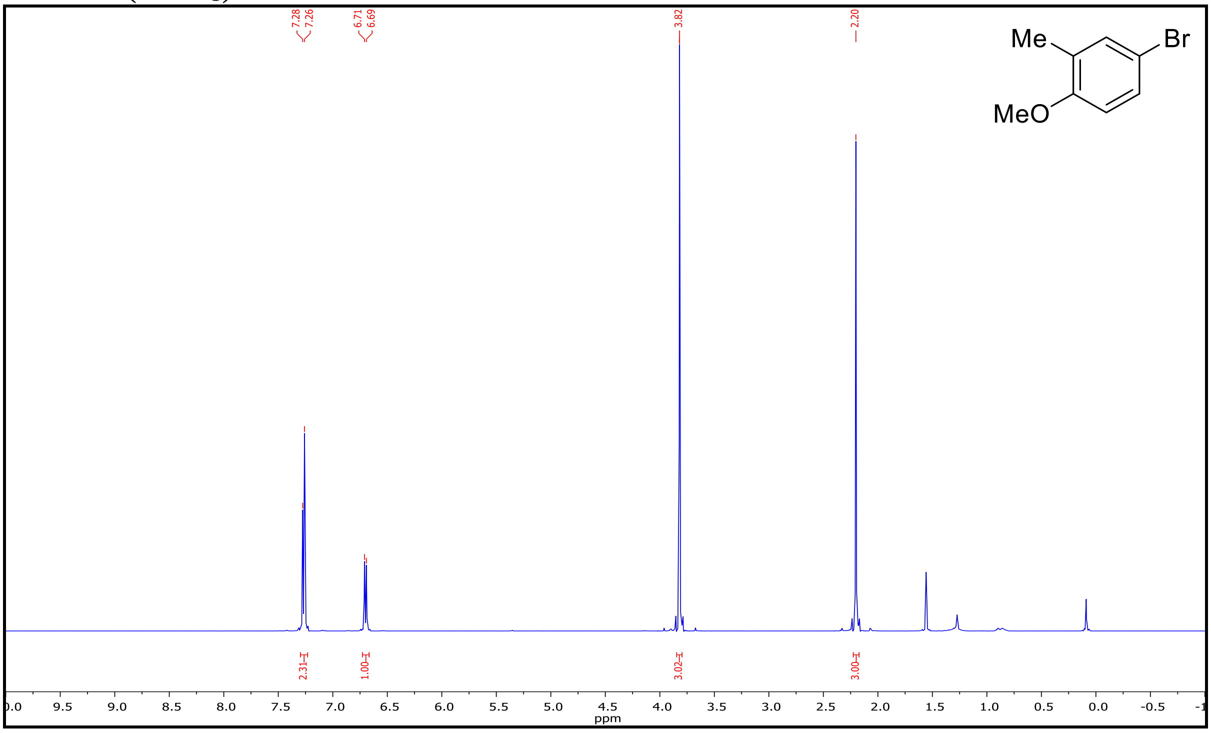

^{13}C NMR ($CDCl_3$)

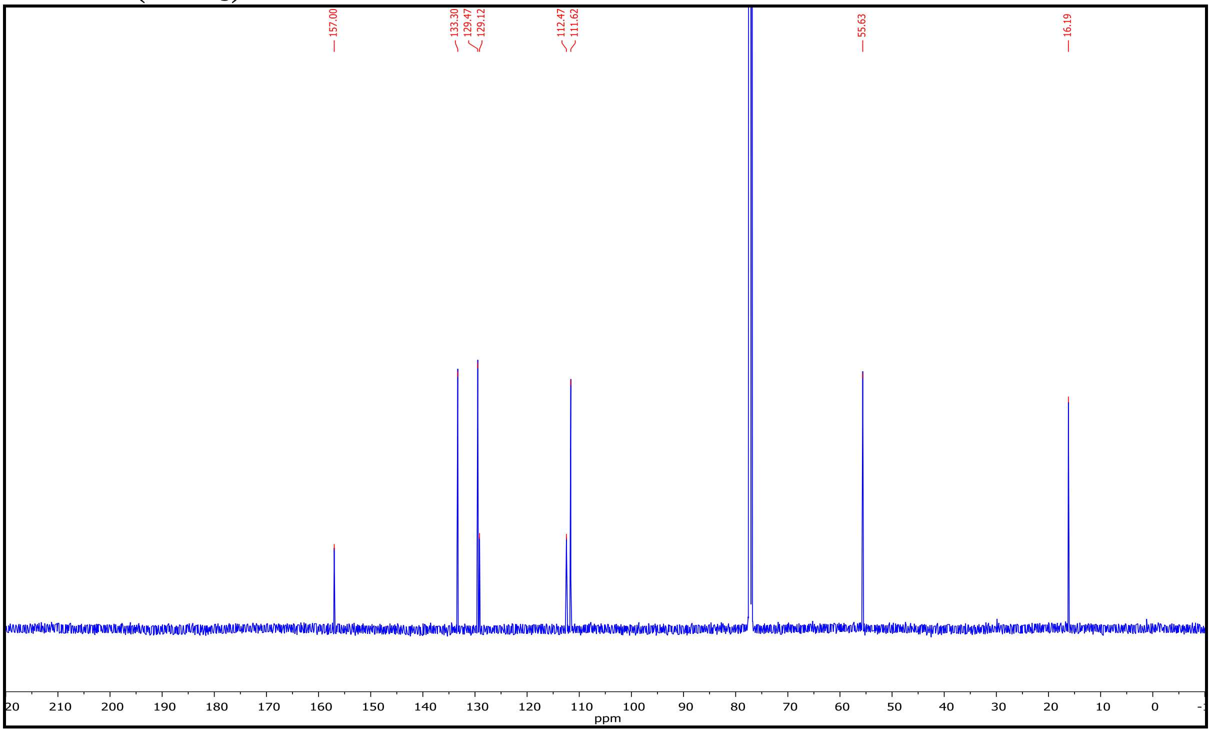

2-bromo-1,3,5-trimethylbenzene (2i)

^{1}H NMR ($CDCl_3$)

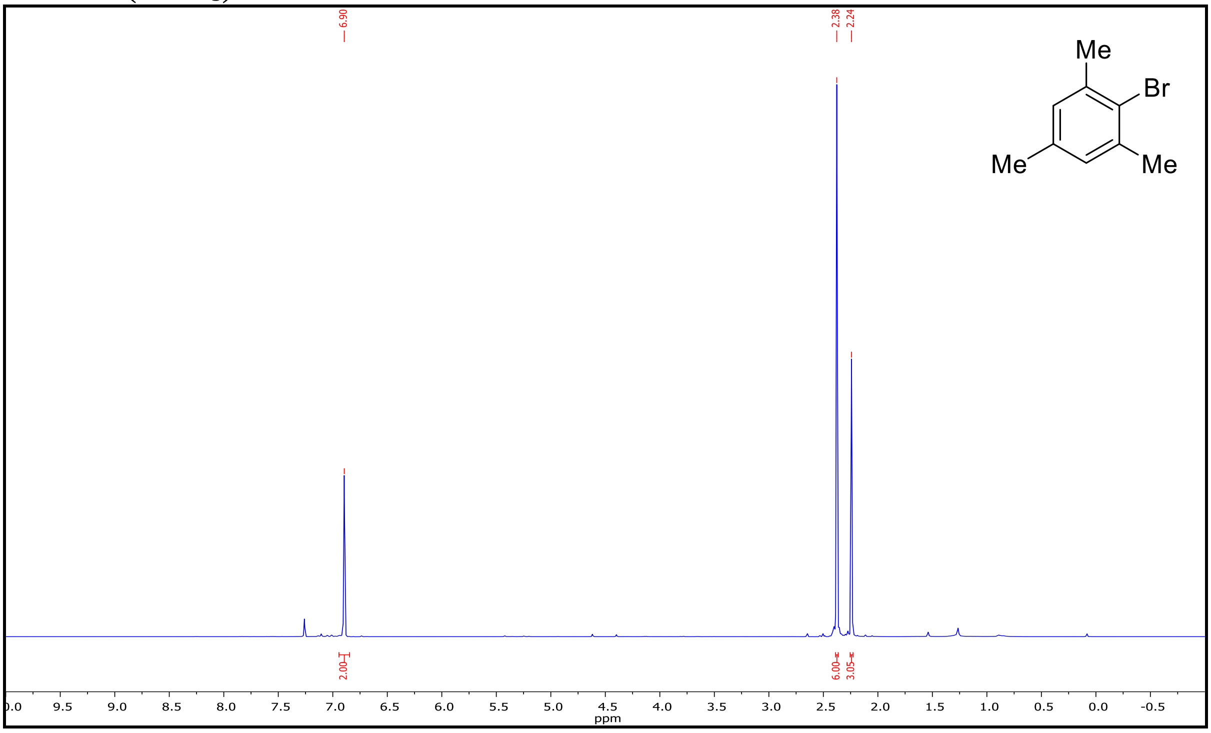

^{13}C NMR ($CDCl_3$)

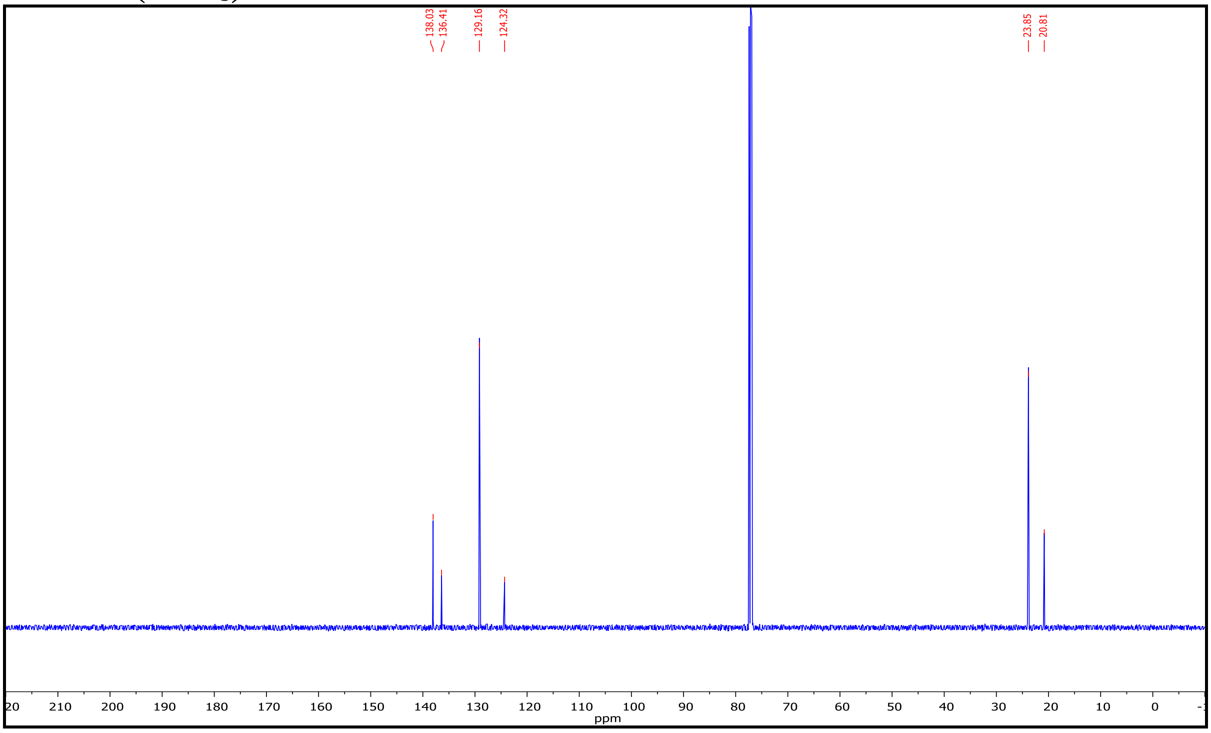

1-bromo-2,4-dimethylbenzene (2j)

^{1}H NMR ($CDCl_3$)

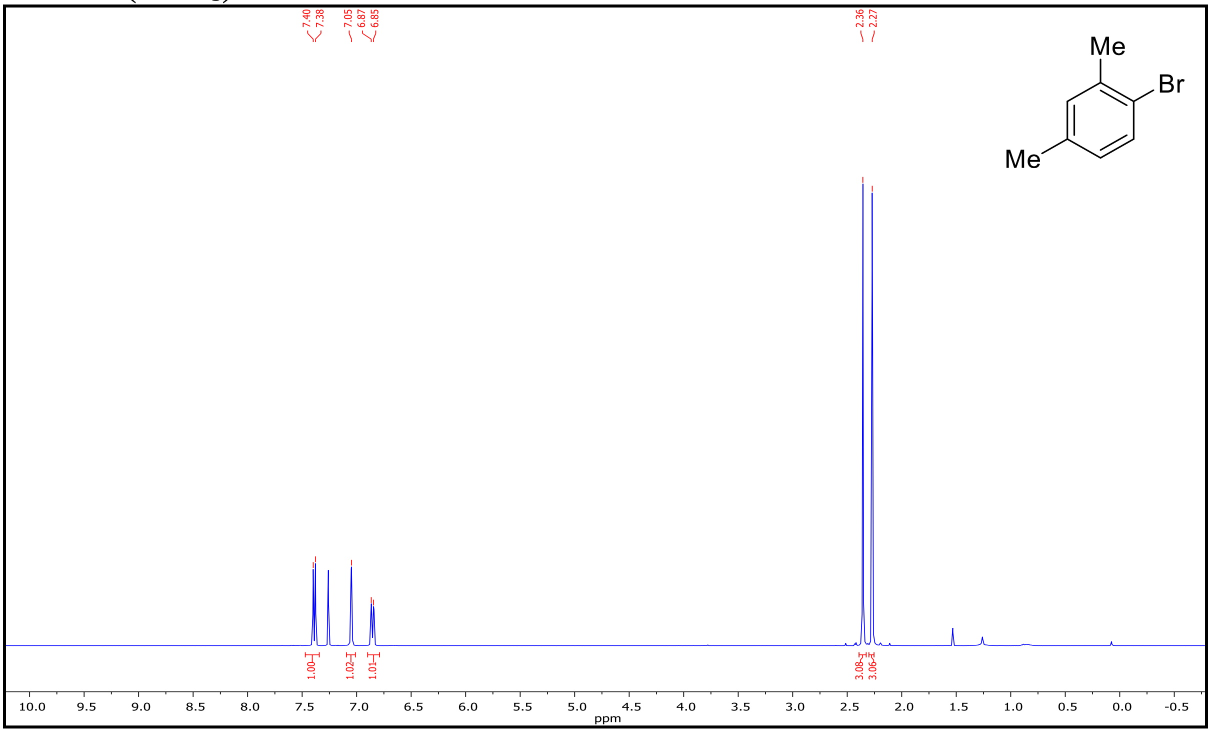

^{13}C NMR ($CDCl_3$)

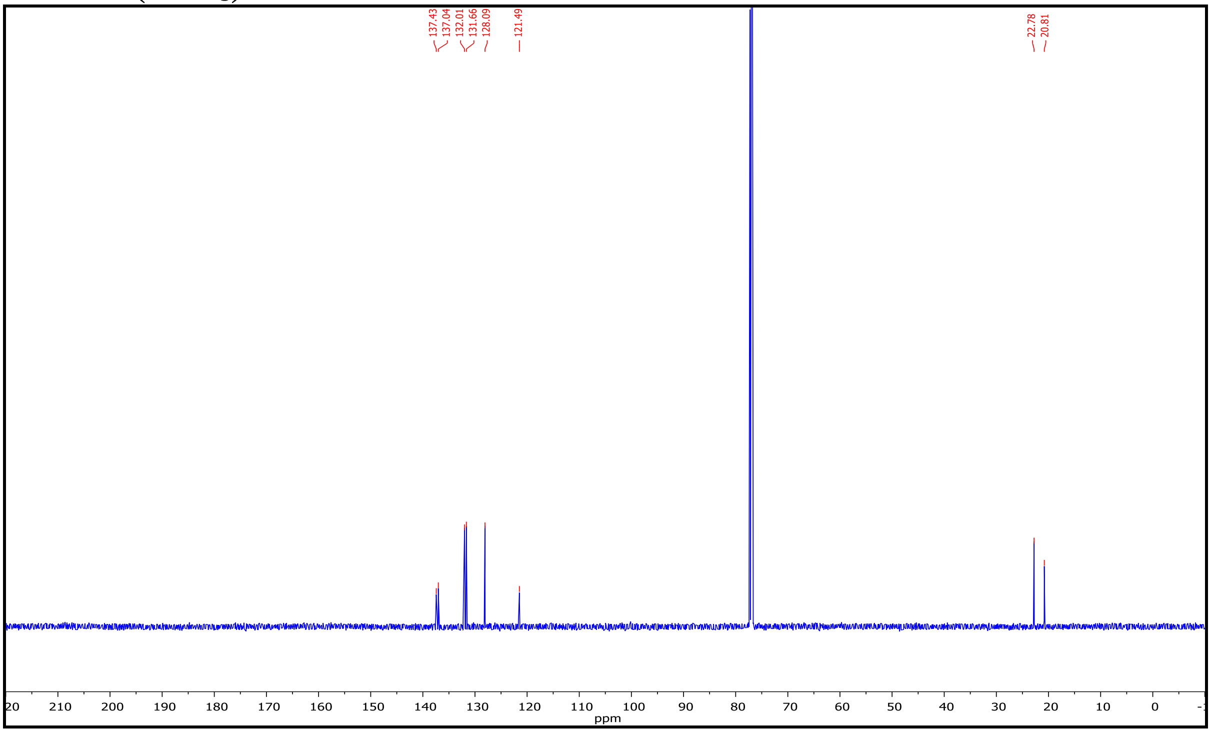

2-bromo-1,3-dimethylbenzene (2k)

^{1}H NMR ($CDCl_3$)

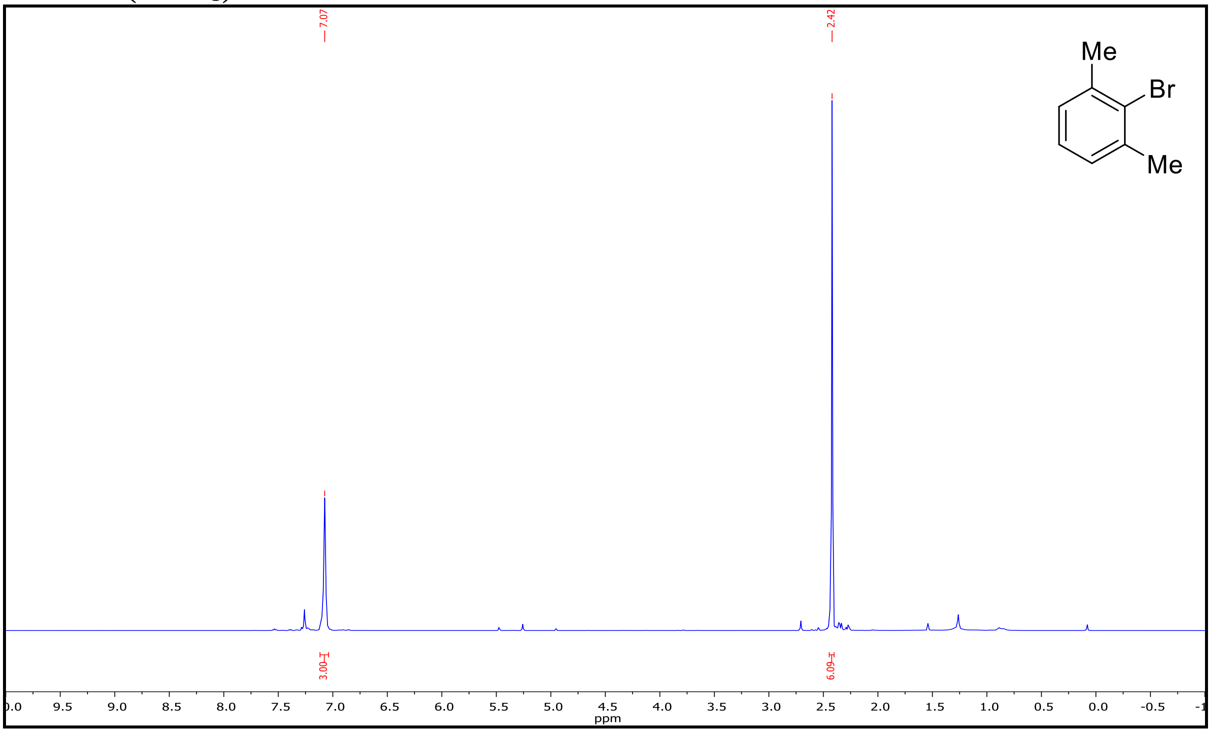

^{13}C NMR ($CDCl_3$)

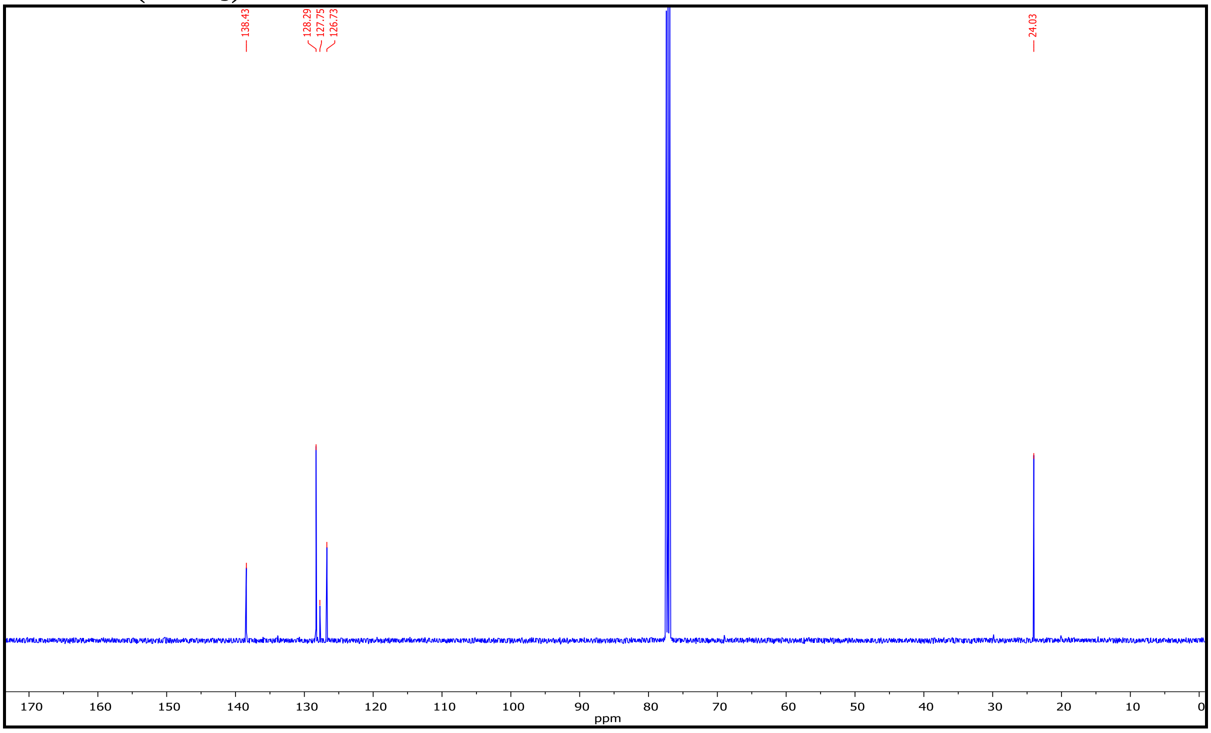

1-bromo-2-methylbenzene (2l)

^{1}H NMR ($CDCl_3$)

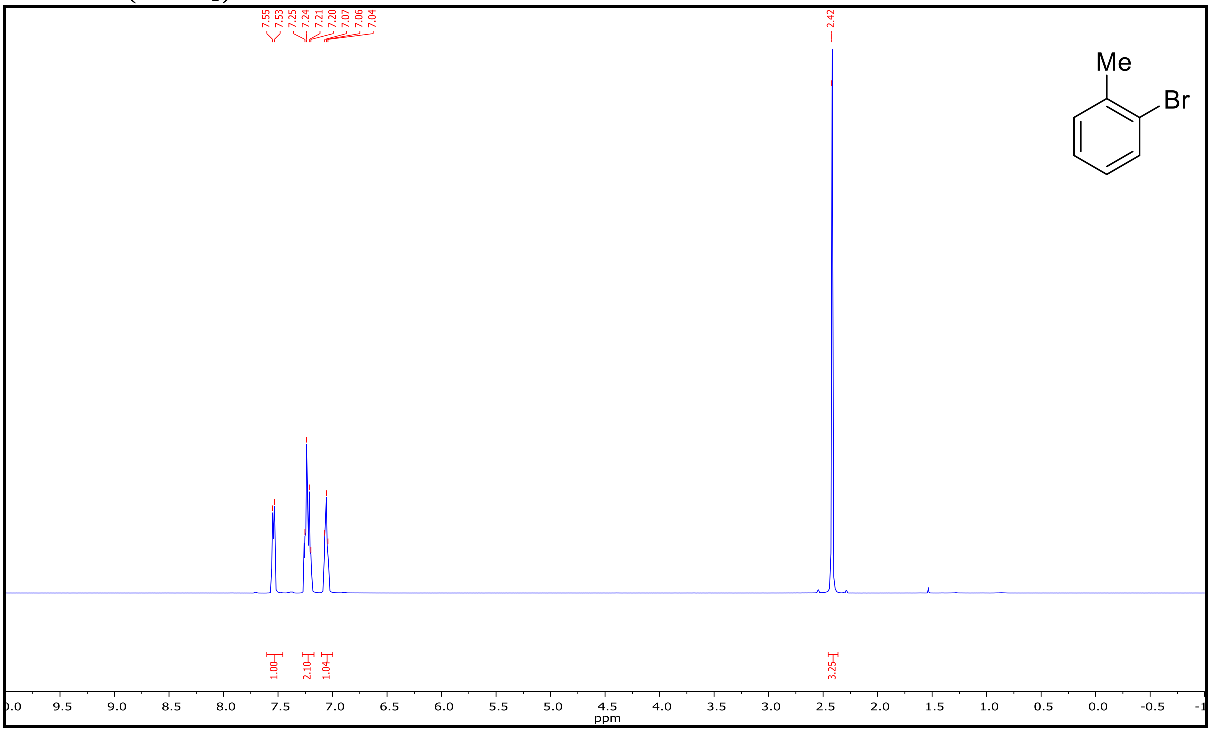

^{13}C NMR ($CDCl_3$)

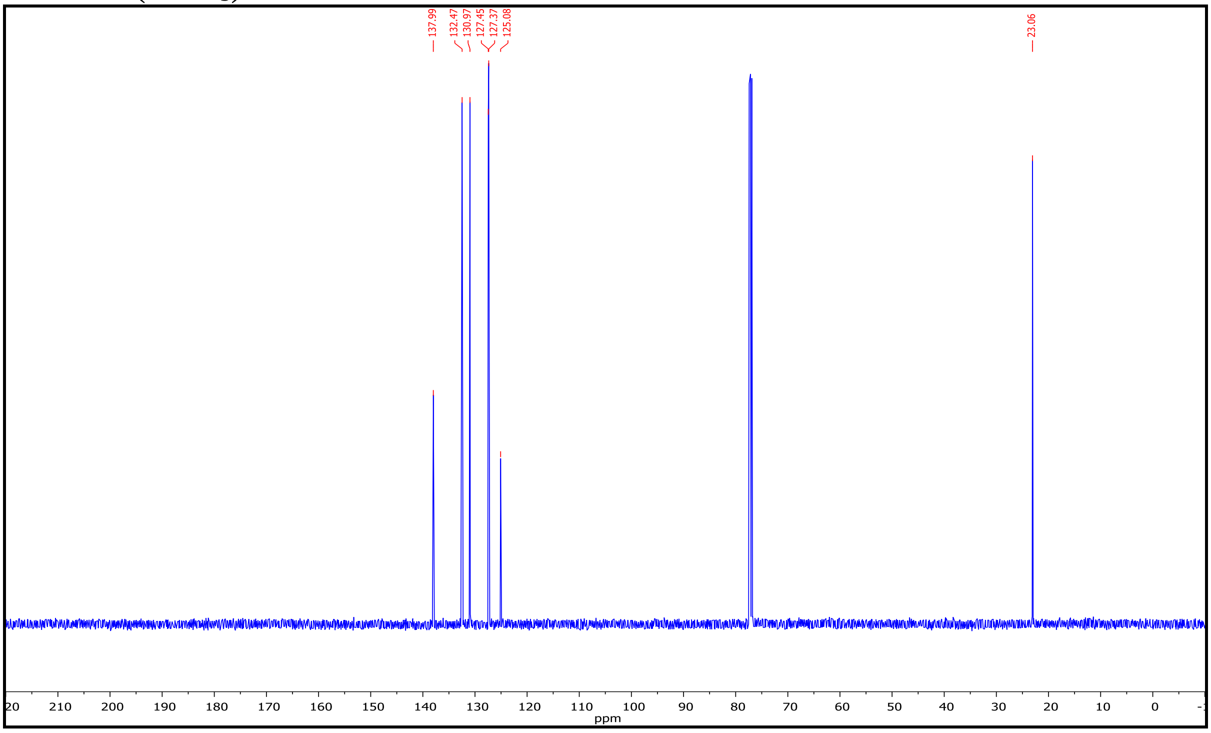

1-bromonaphthalene (2m)

^{1}H NMR ($CDCl_3$)

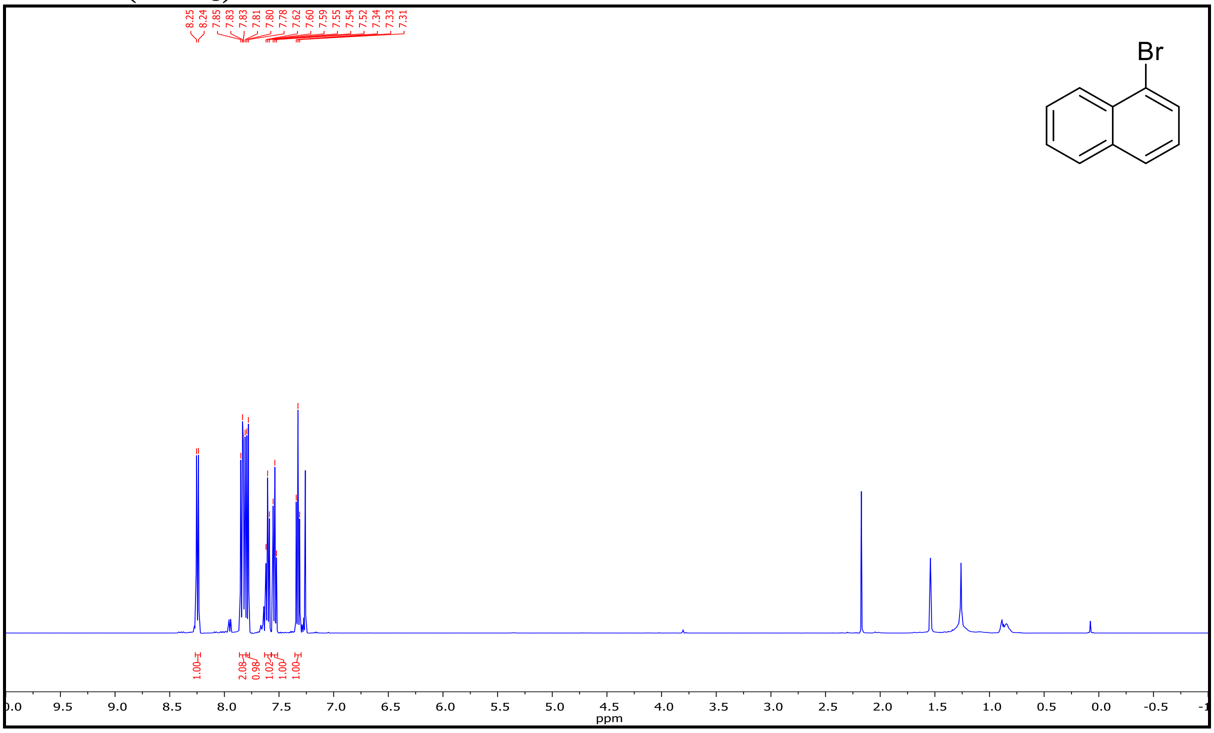

^{13}C NMR ($CDCl_3$)

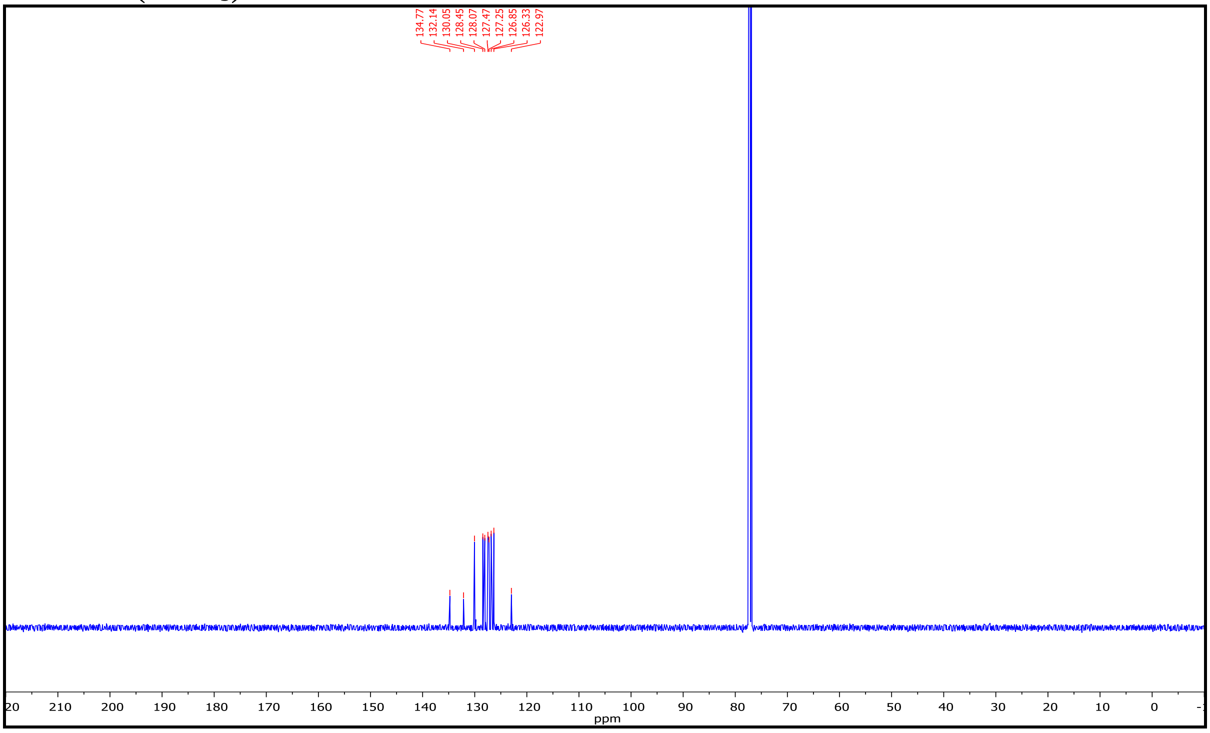

1,4-dibromonaphthalene (2m')

^{1}H NMR ($CDCl_3$)

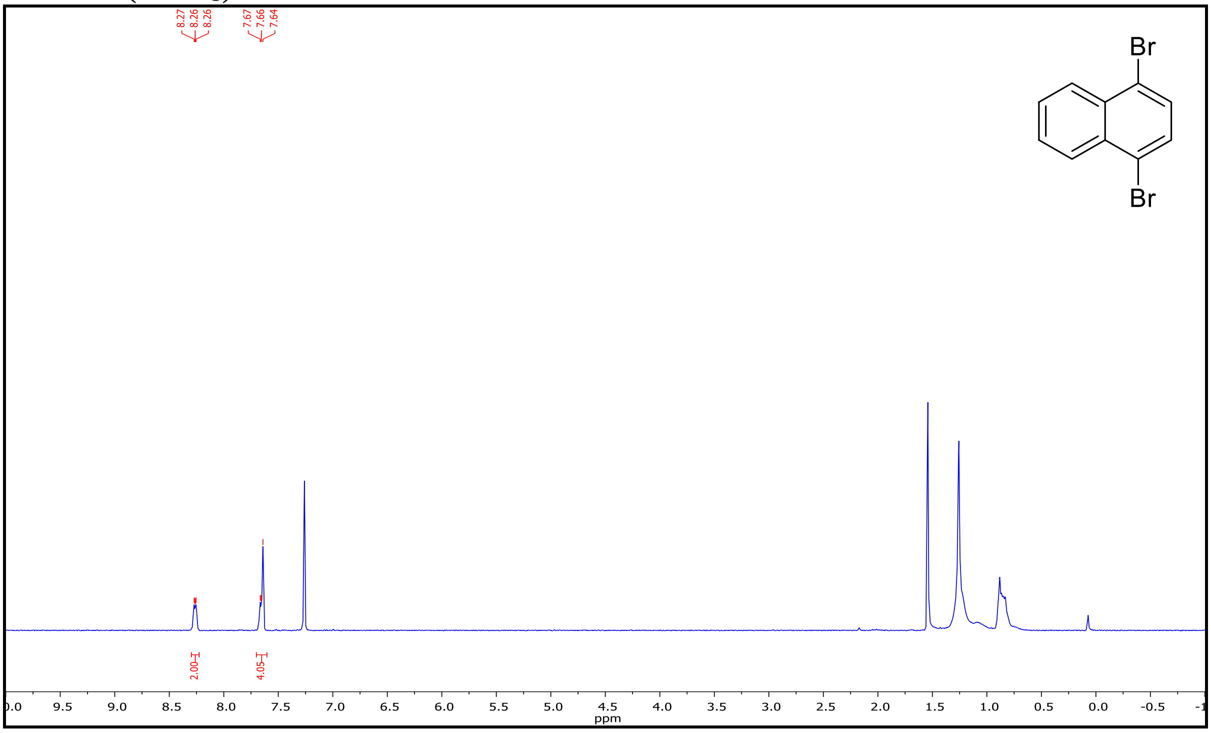

^{13}C NMR ($CDCl_3$)

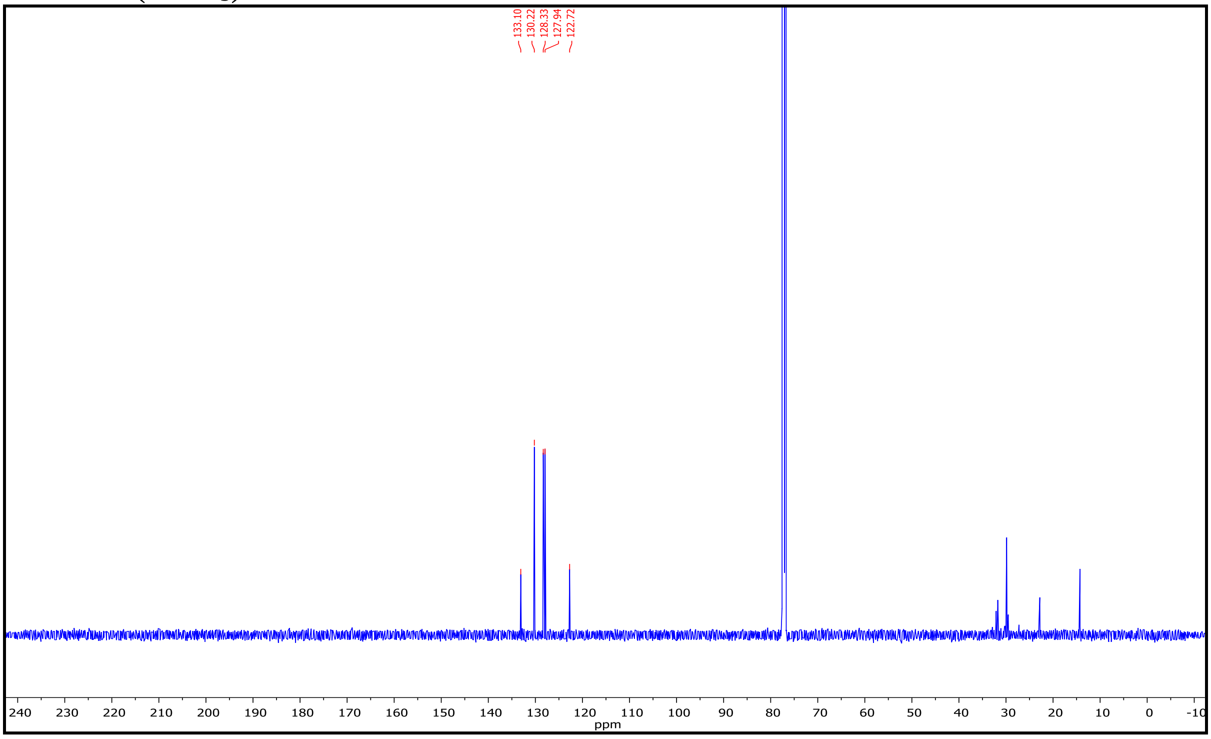

1-bromo-2-methylnaphthalene (2n)

^{1}H NMR ($CDCl_3$)

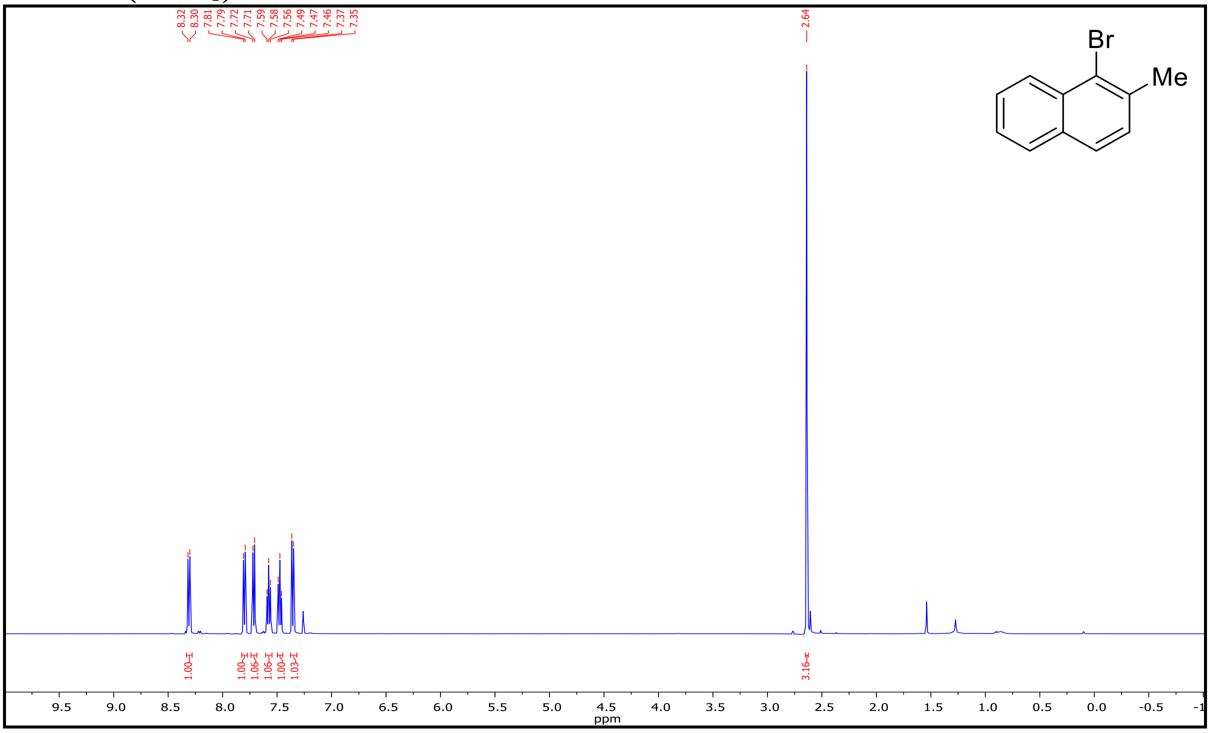

^{13}C NMR ($CDCl_3$)

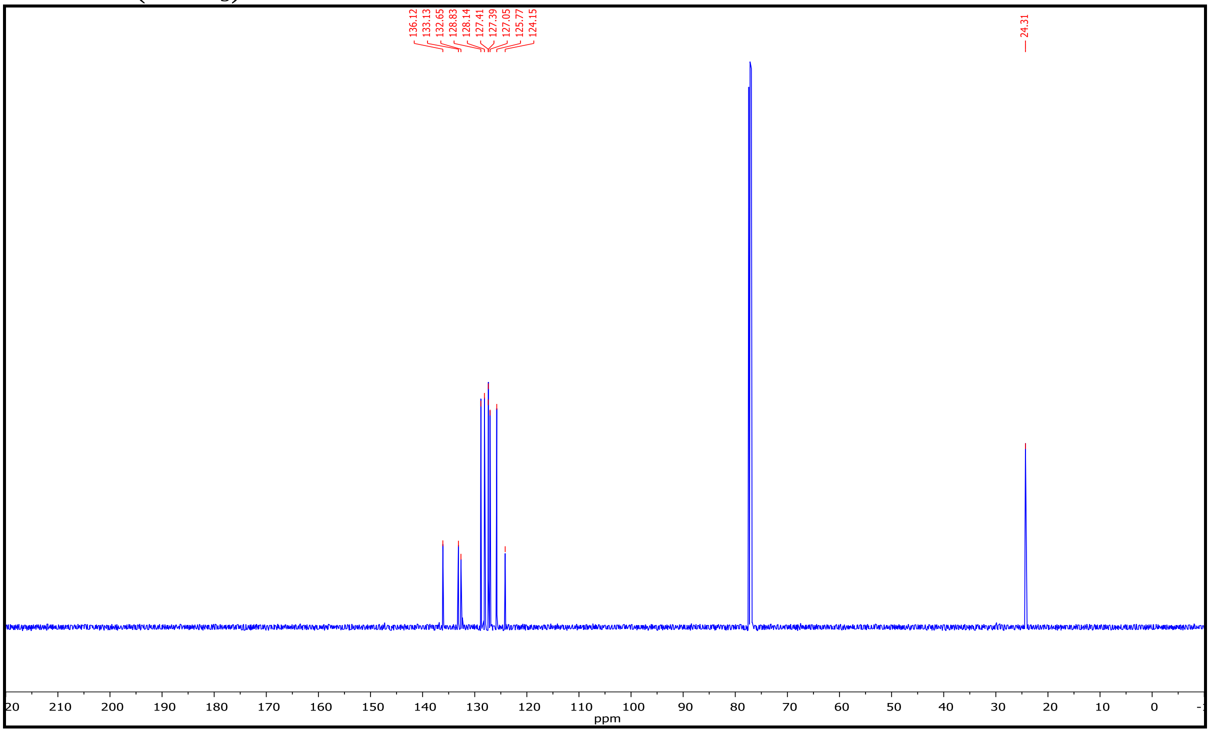

1,3-dibromo-2-methylnaphthalene (2n')

^{1}H NMR ($CDCl_3$)

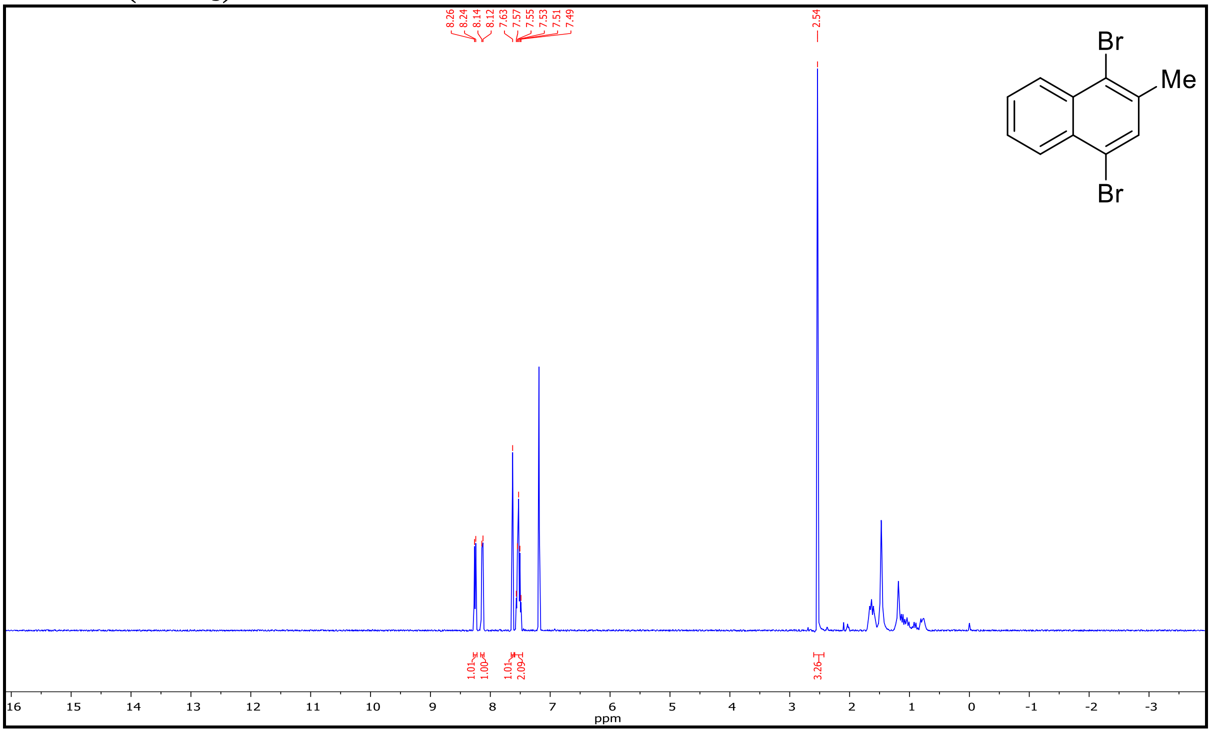

^{13}C NMR ($CDCl_3$)

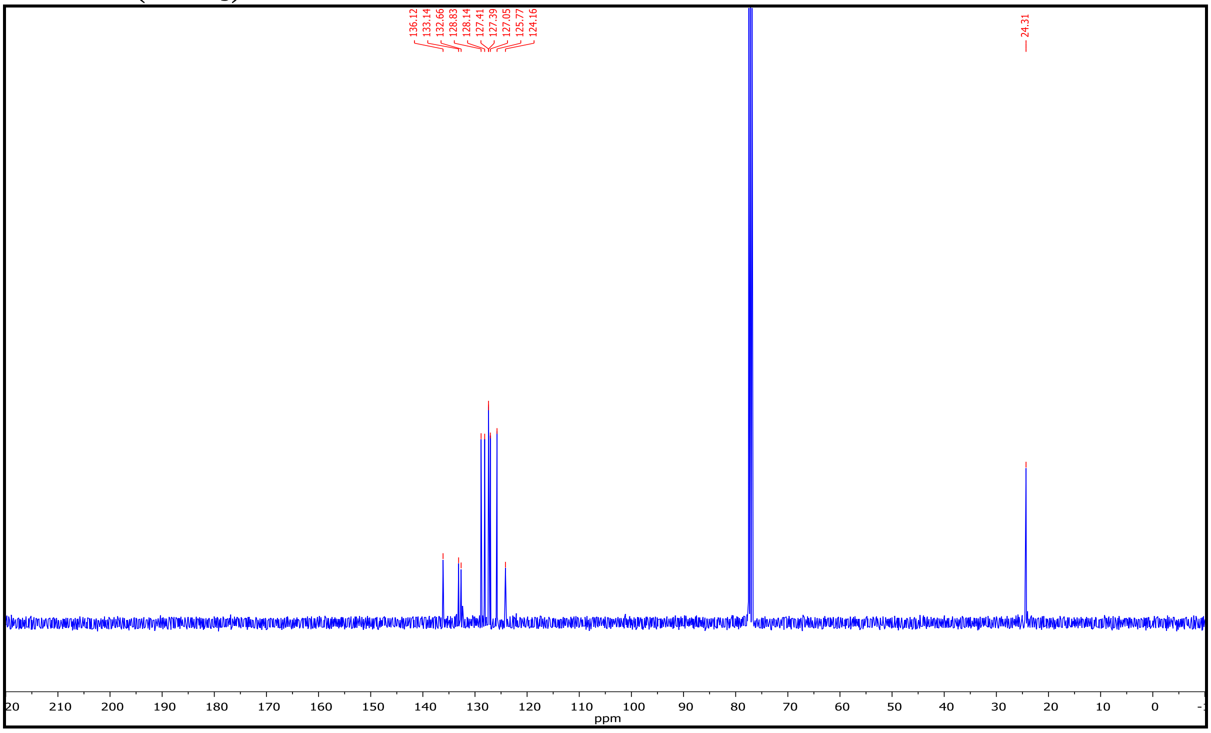

1-bromo-4-fluoro-2-methoxybenzene (2o)

^{1}H NMR ($CDCl_3$)

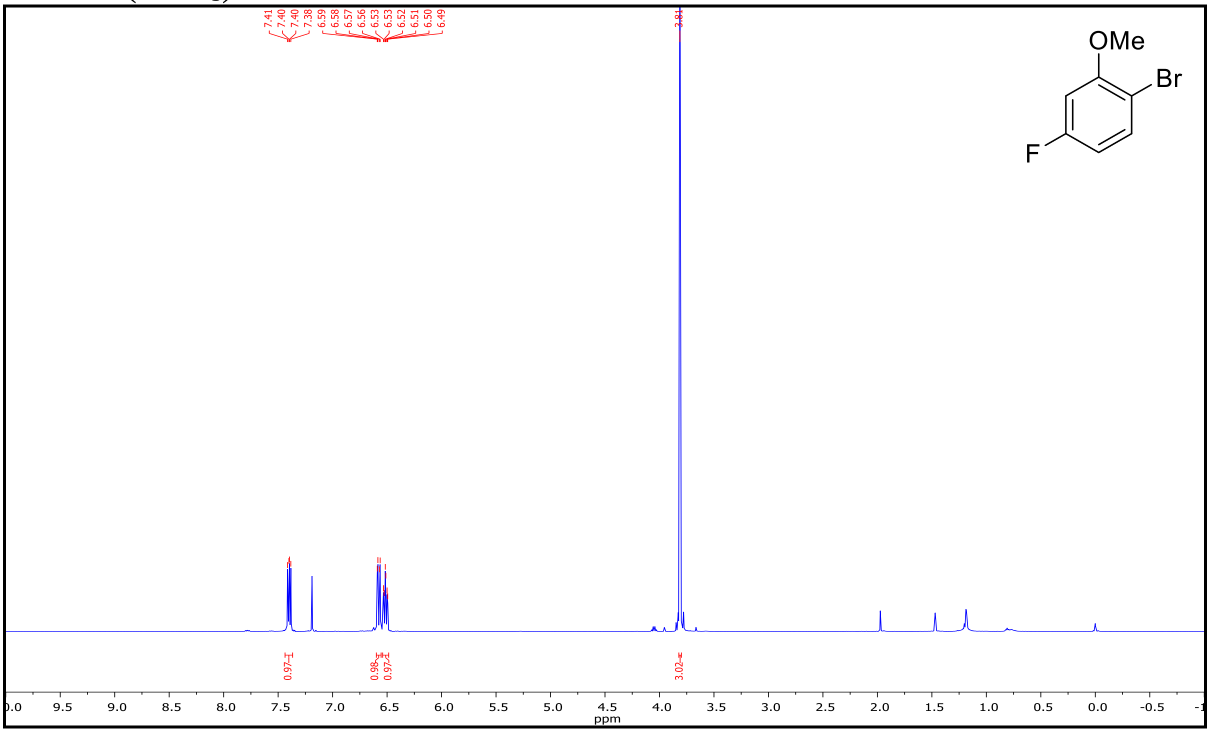

^{13}C NMR ($CDCl_3$)

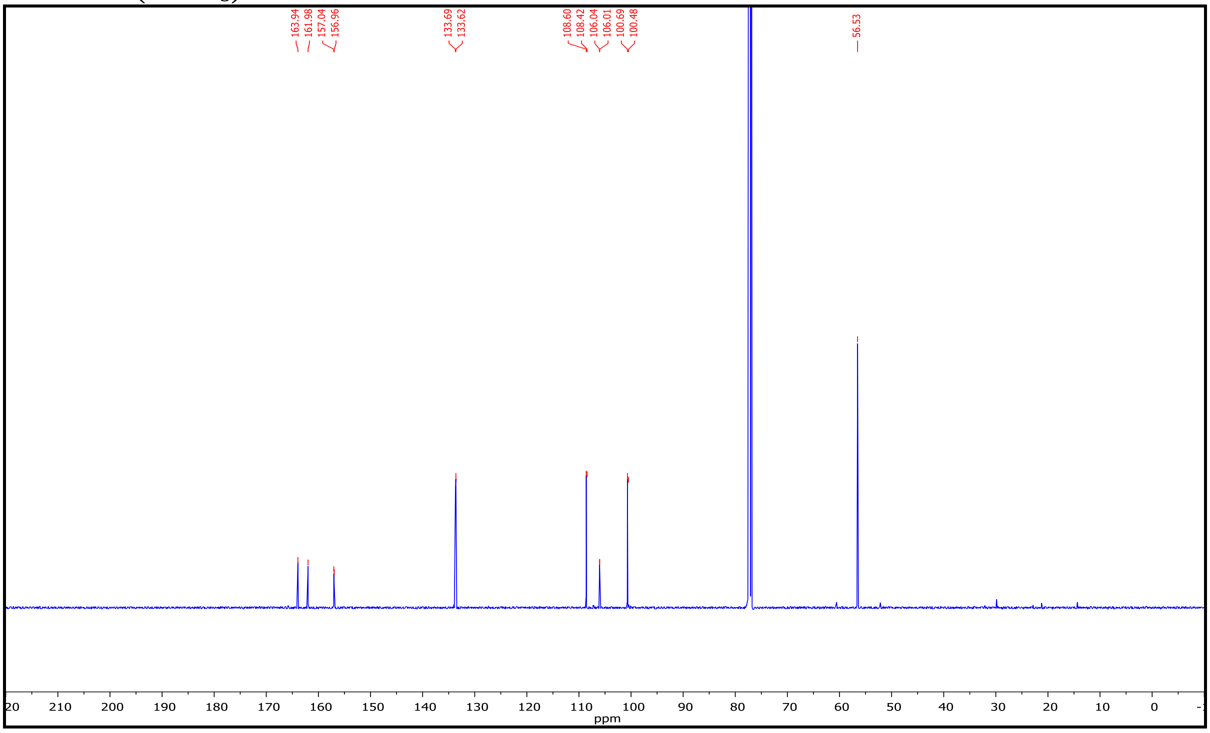

^{19}F NMR ($CDCl_3$)

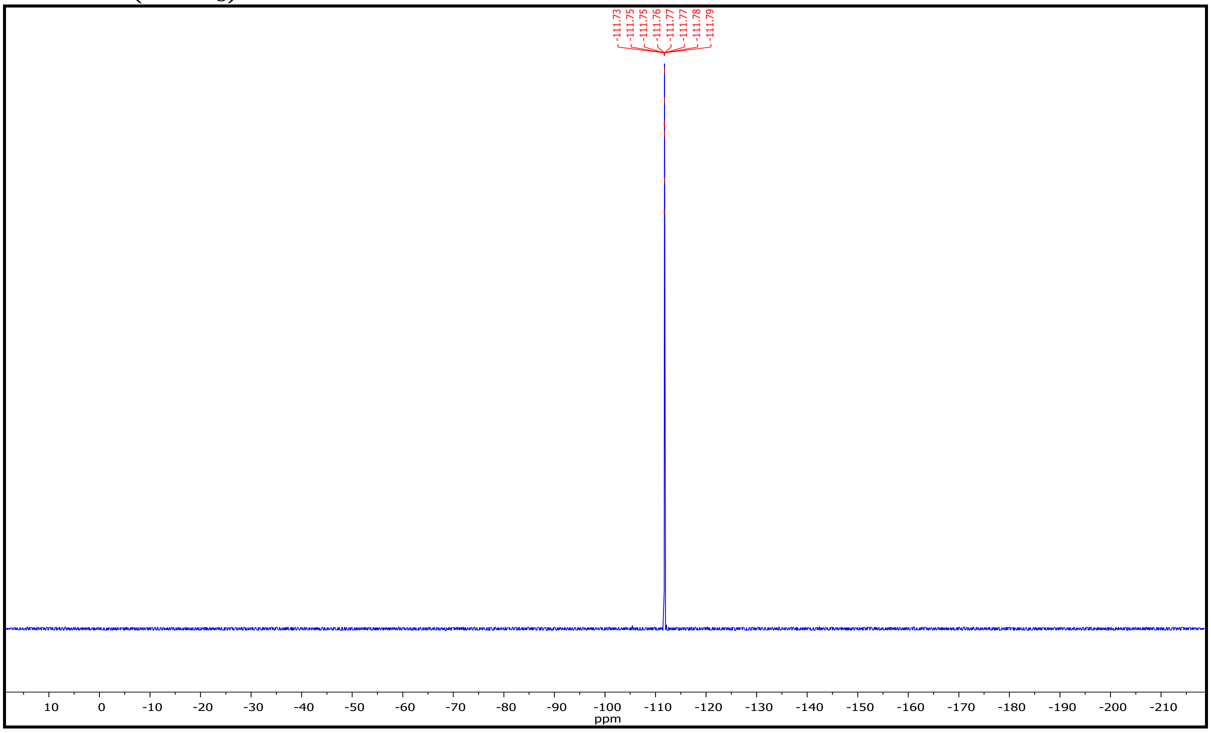

1-bromo-4-chloro-2-methoxybenzene (2p)

^{1}H NMR ($CDCl_3$)

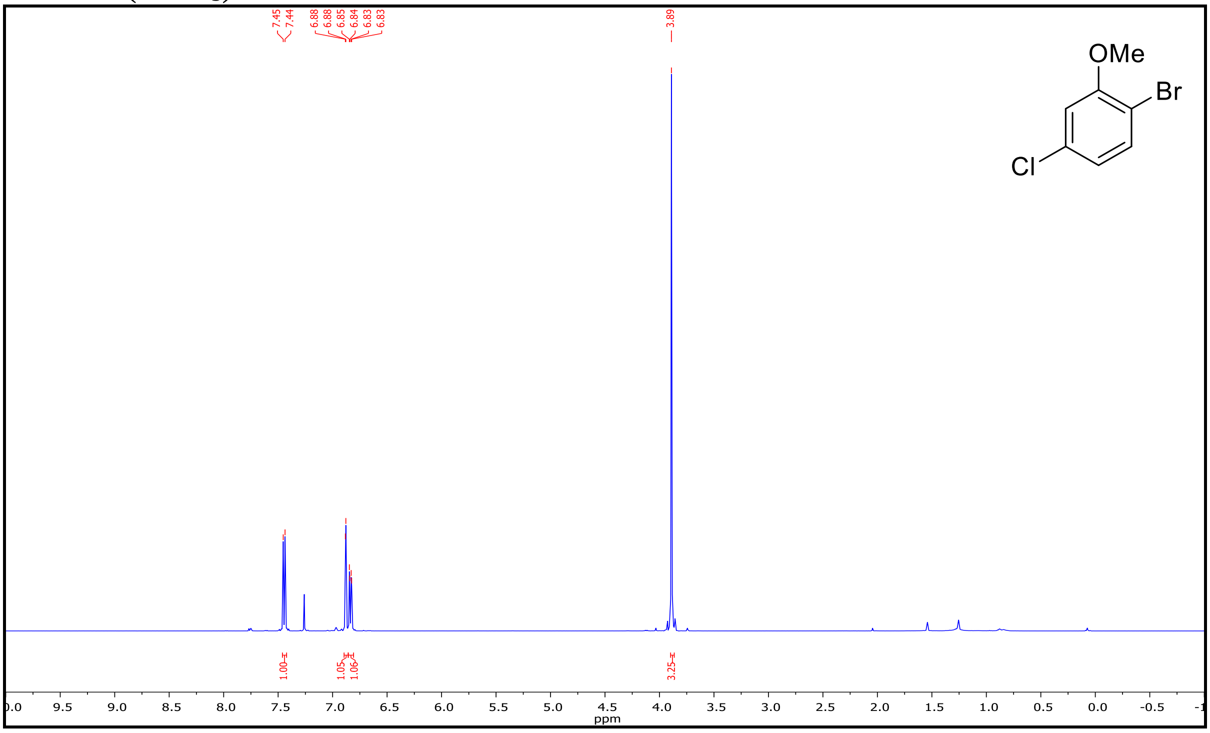

^{13}C NMR ($CDCl_3$)

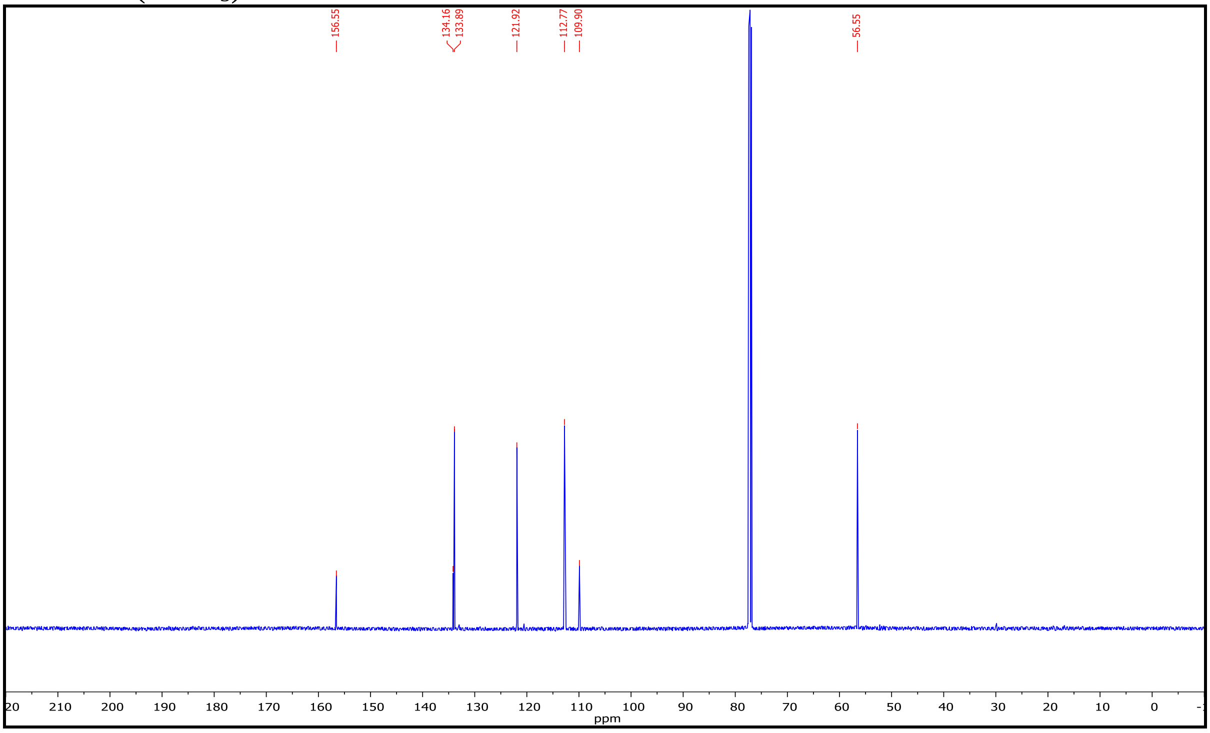

1,4-dibromo-2-methoxybenzene (2q)

^{1}H NMR ($CDCl_3$)

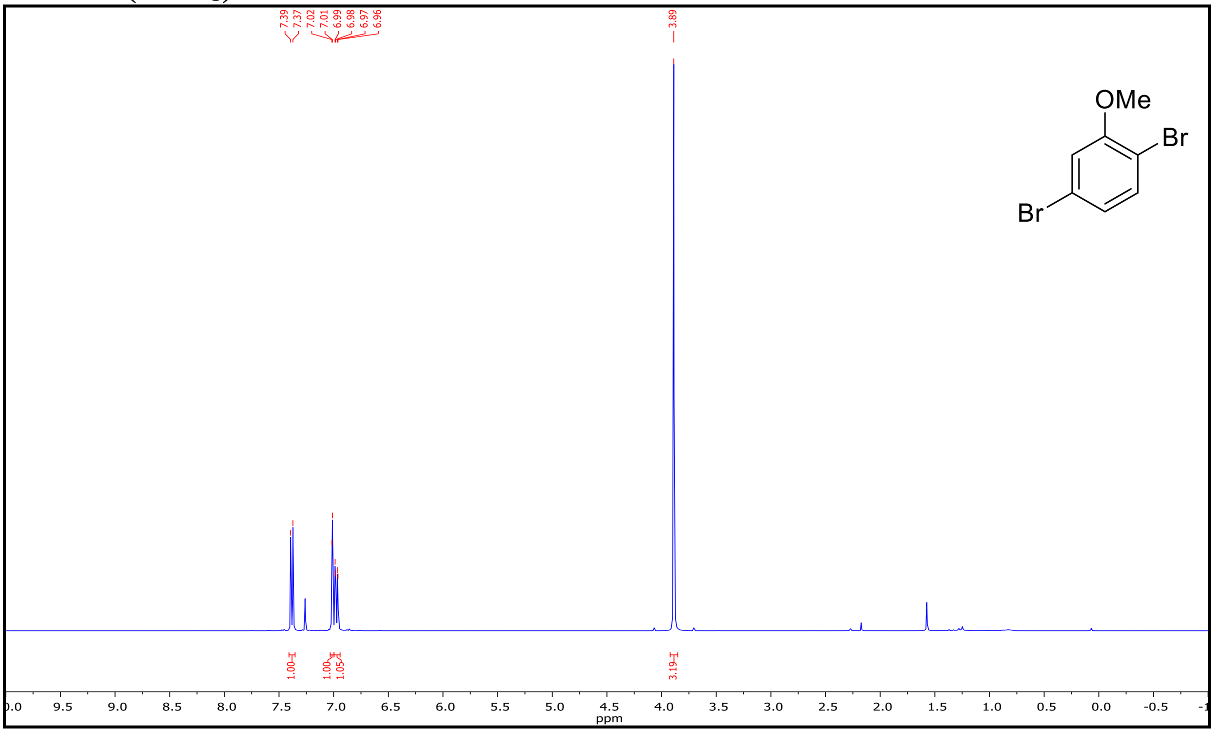

^{13}C NMR ($CDCl_3$)

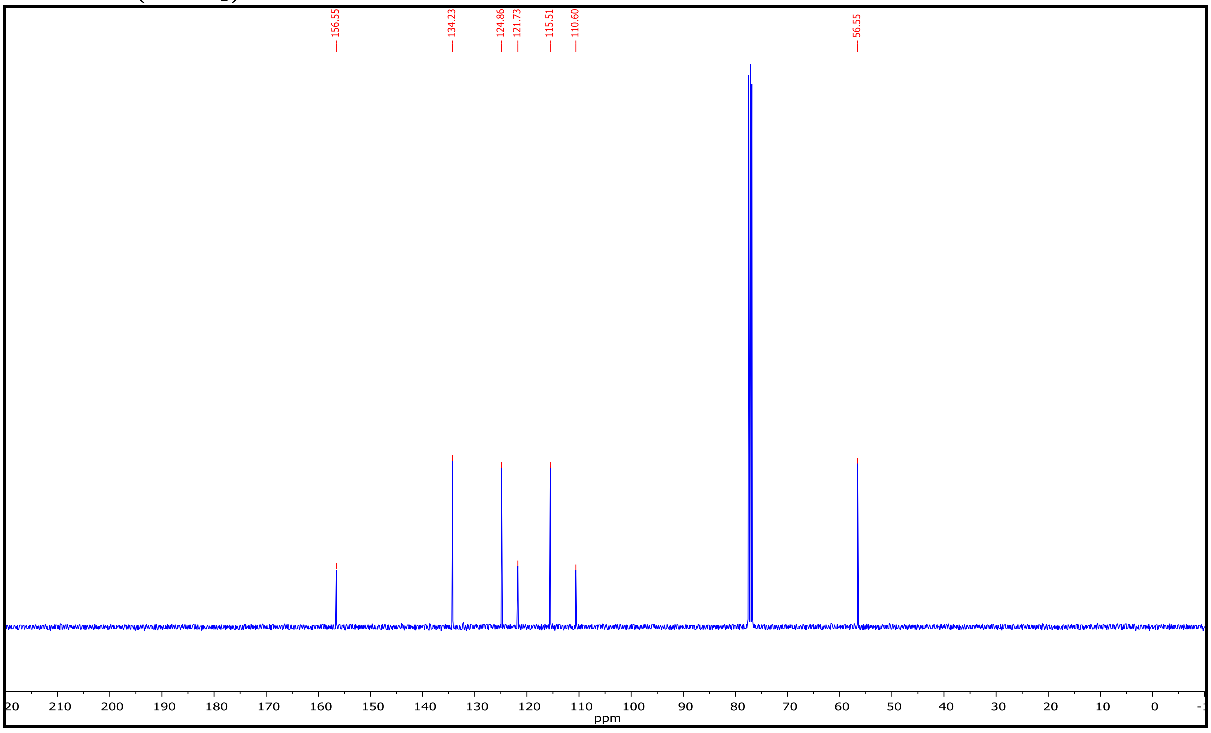

1-bromo-4-iodo-2-methoxybenzene (2r)

^{1}H NMR ($CDCl_3$)

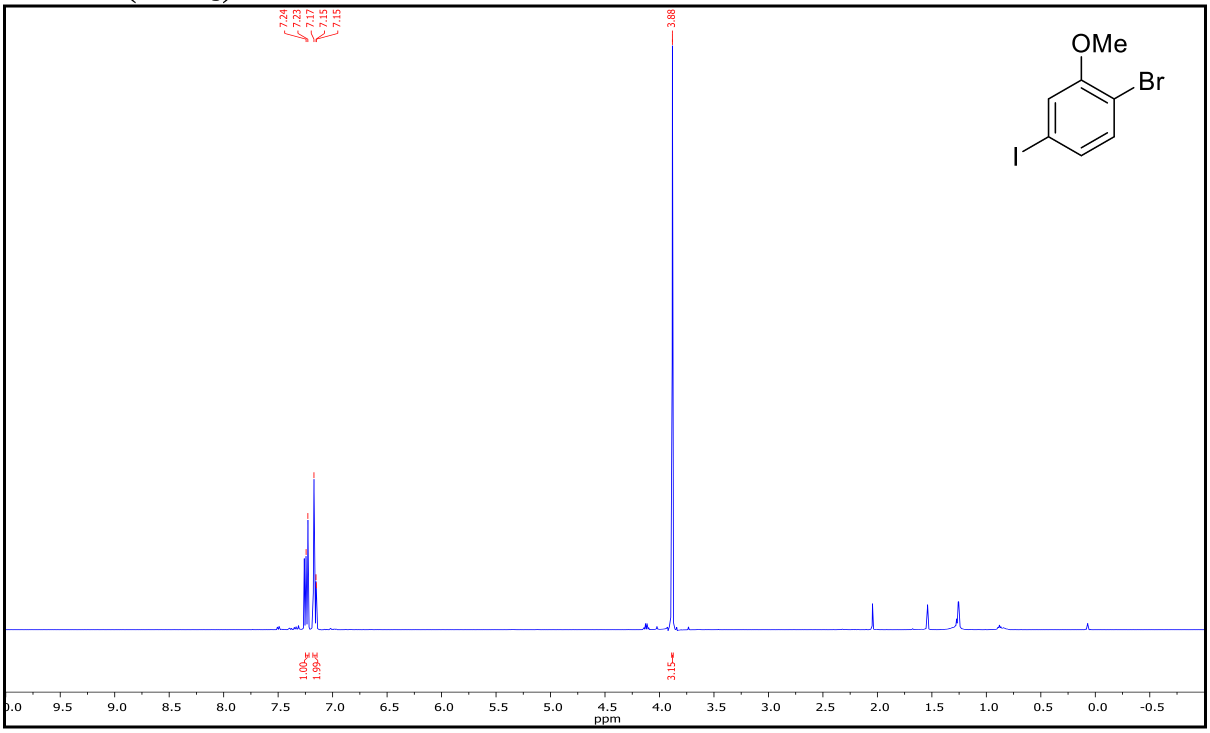

^{13}C NMR ($CDCl_3$)

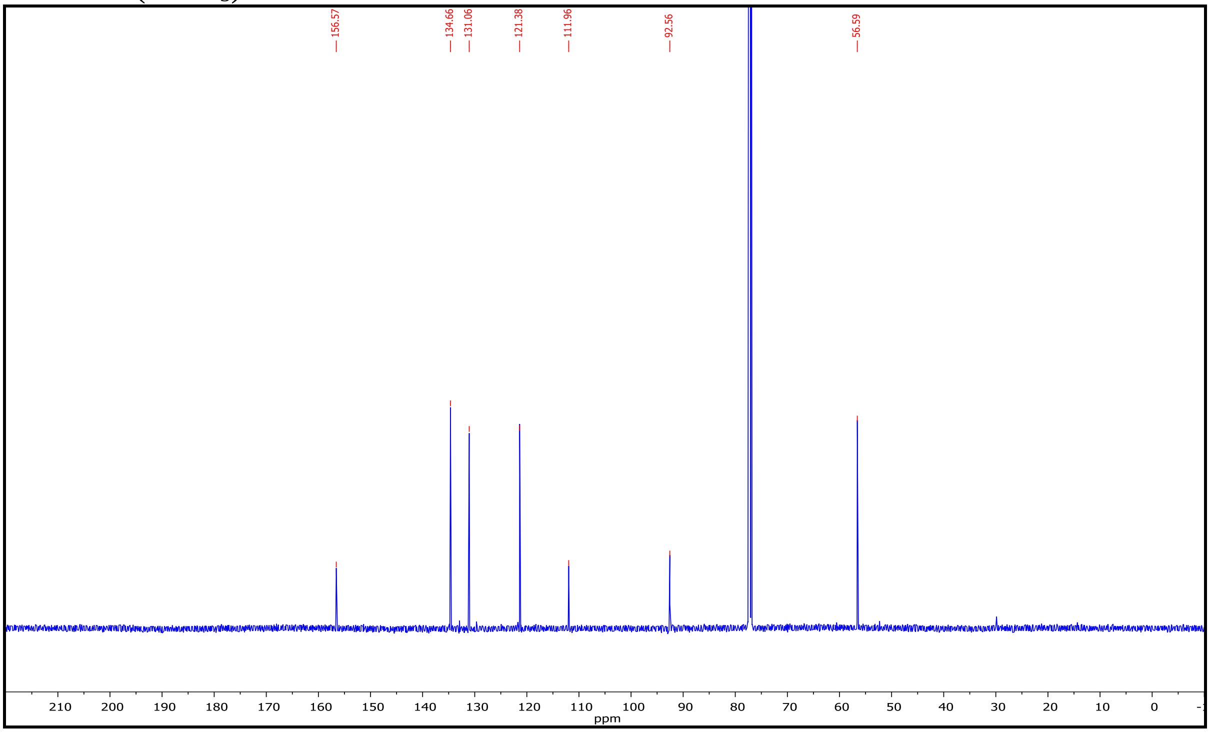

2-bromo-4-fluoro-1-methoxybenzene (2s)

^{1}H NMR ($CDCl_3$)

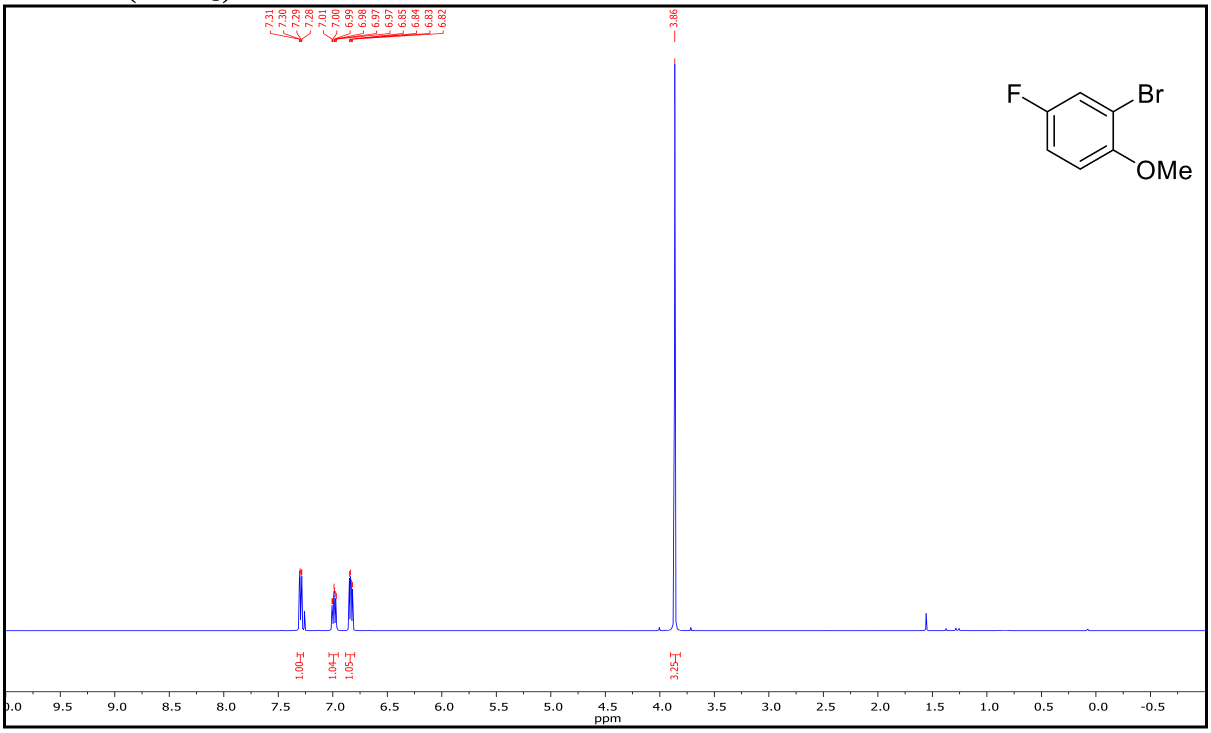

^{13}C NMR ($CDCl_3$)

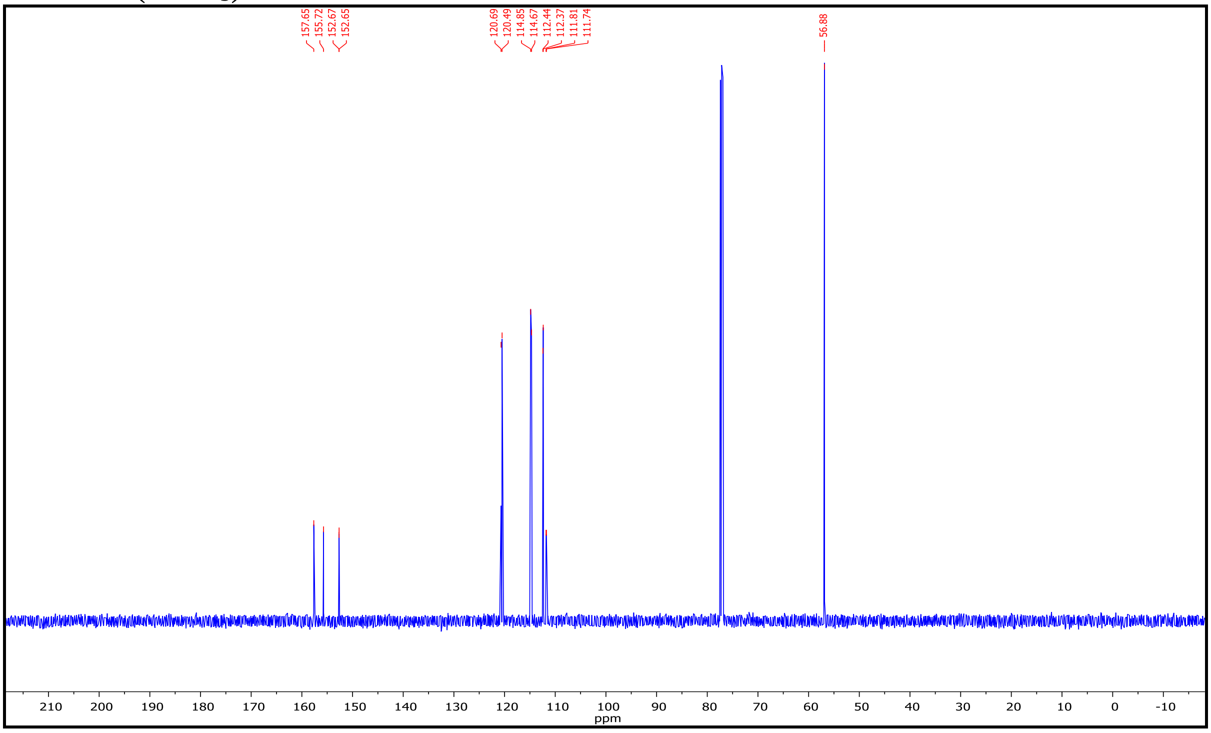

^{19}F NMR ($CDCl_3$)

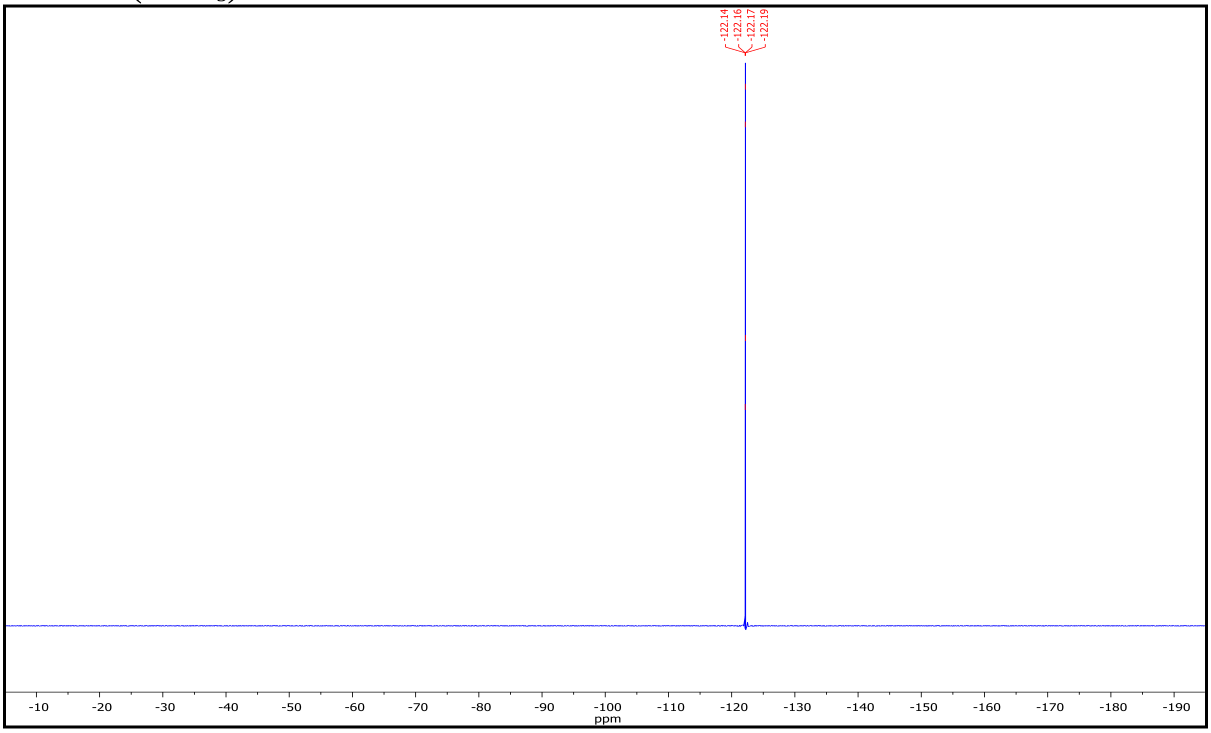

2-bromo-4-chloro-1-methoxybenzene (2t)

^{1}H NMR ($CDCl_3$)

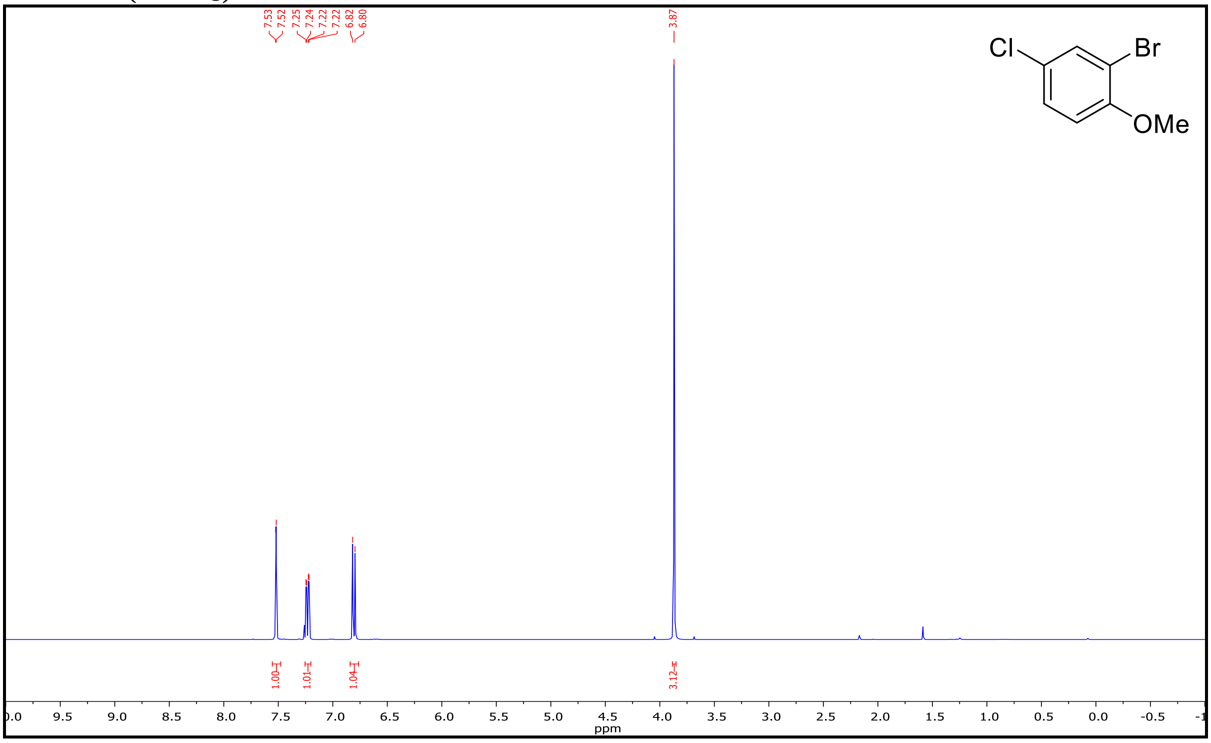

^{13}C NMR ($CDCl_3$)

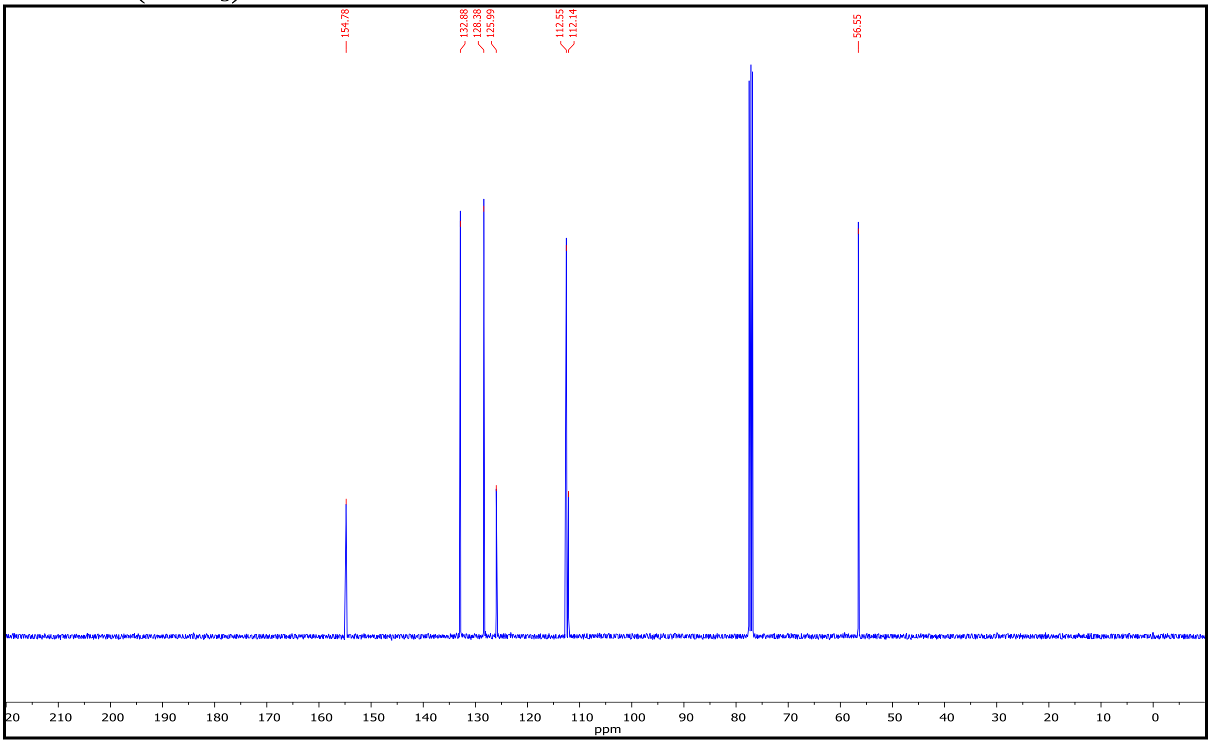

2,4-dibromo-1-methoxybenzene (2u)

^{1}H NMR (CDCl$_3$)

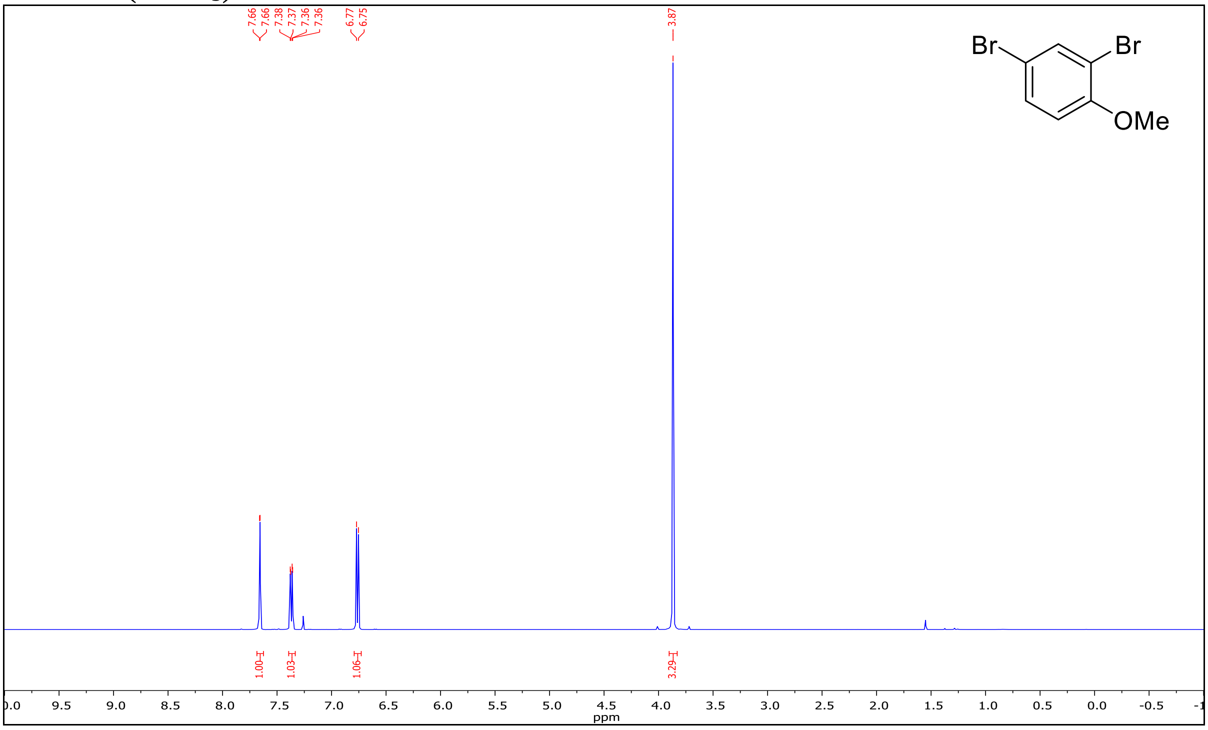

^{13}C NMR (CDCl$_3$)

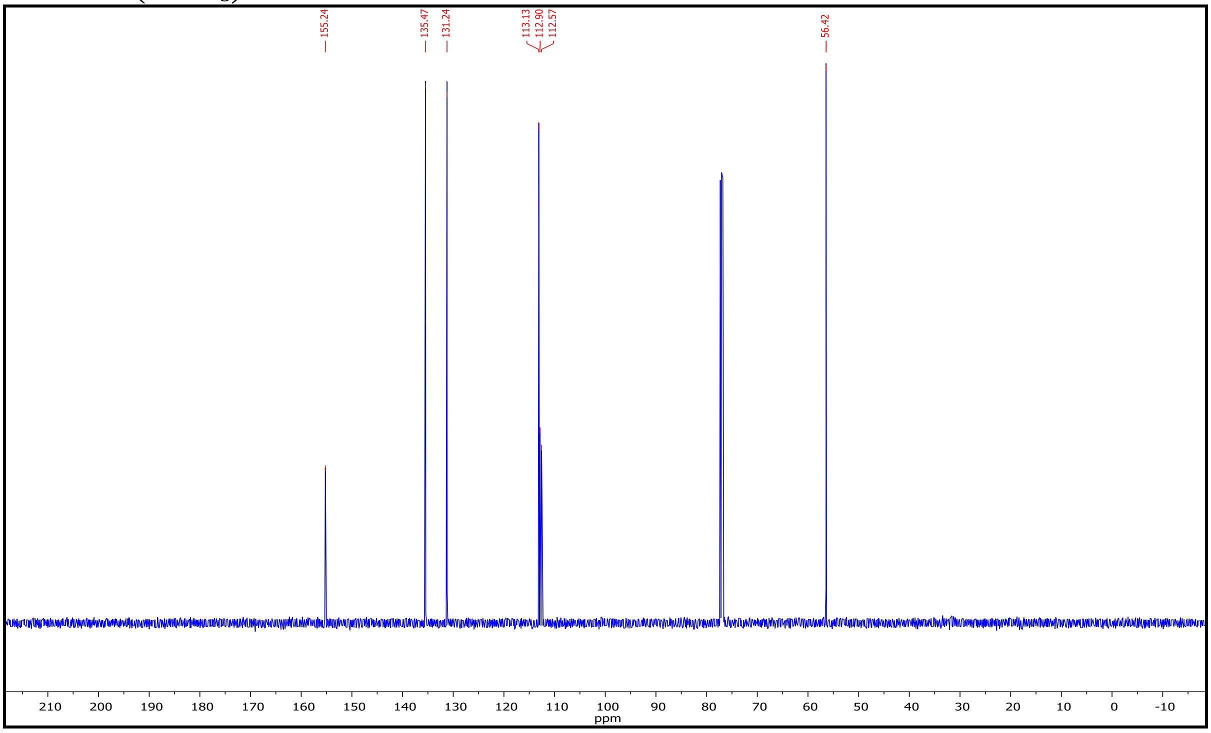

1-bromo-2-fluoro-4-methoxybenzene (2v)

^{1}H NMR ($CDCl_3$)

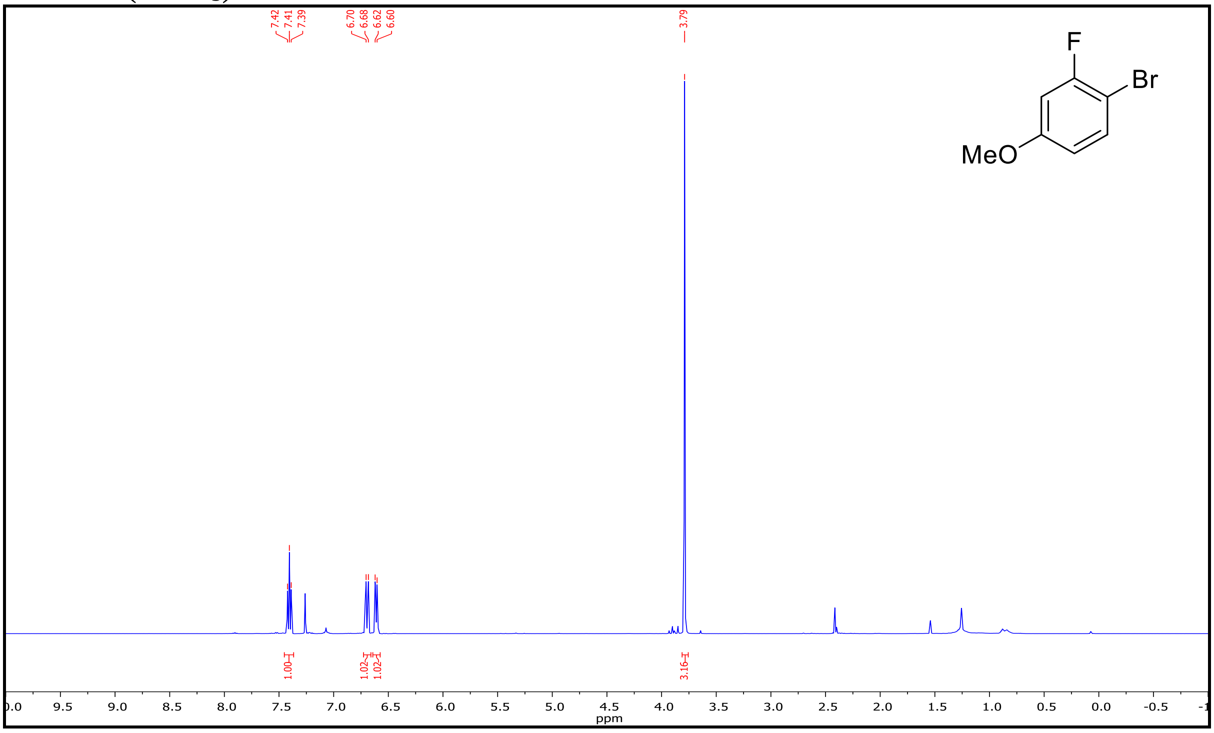

^{13}C NMR ($CDCl_3$)

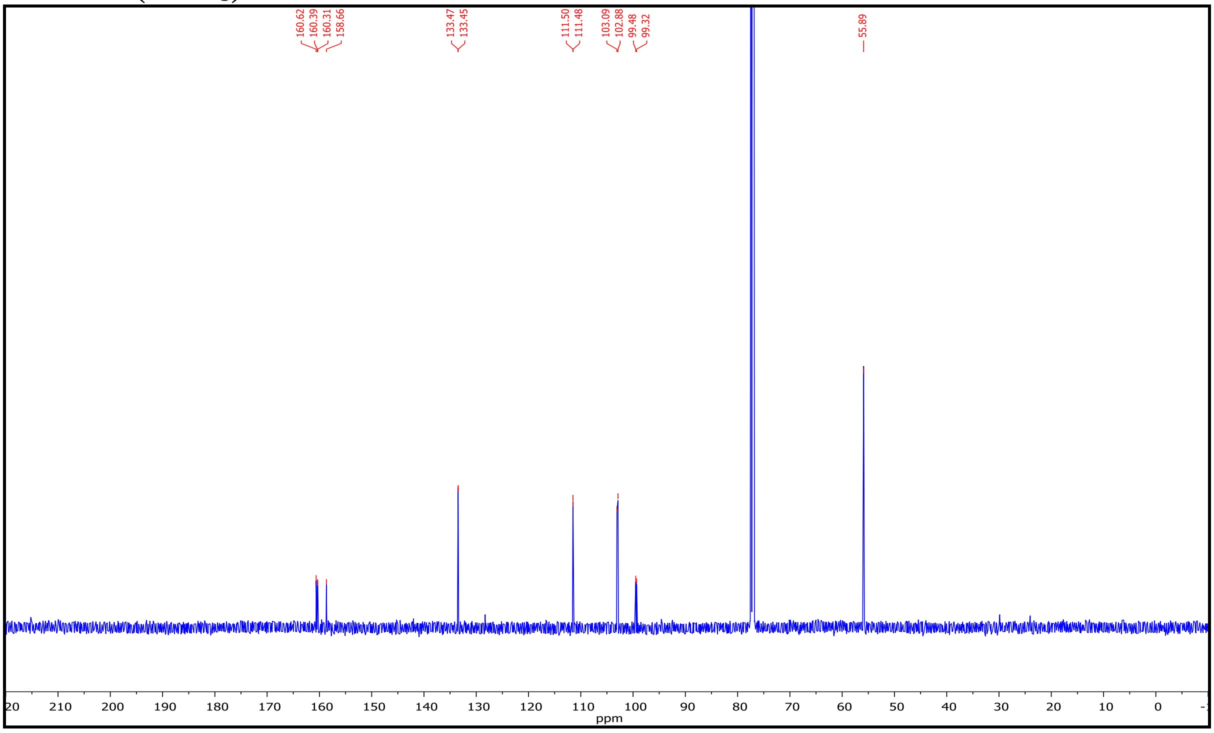

^{19}F NMR ($CDCl_3$)

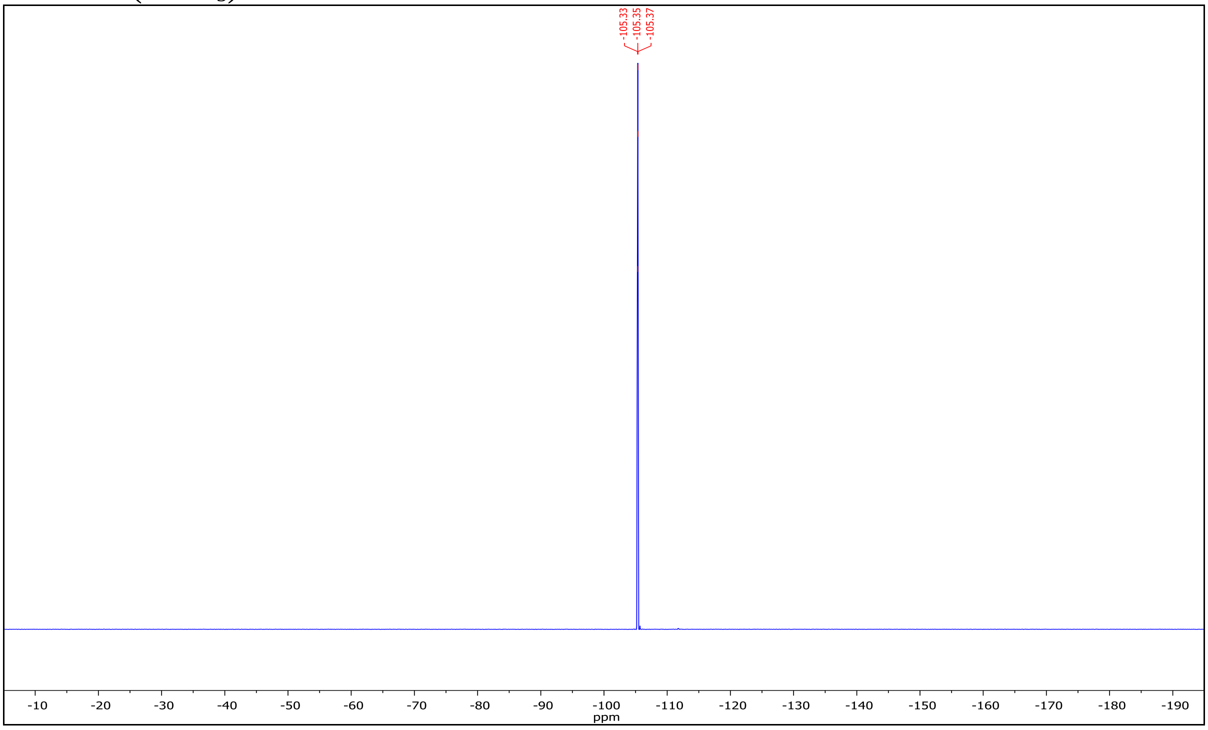

1-bromo-2-chloro-4-methoxybenzene (2w)

^{1}H NMR ($CDCl_3$)

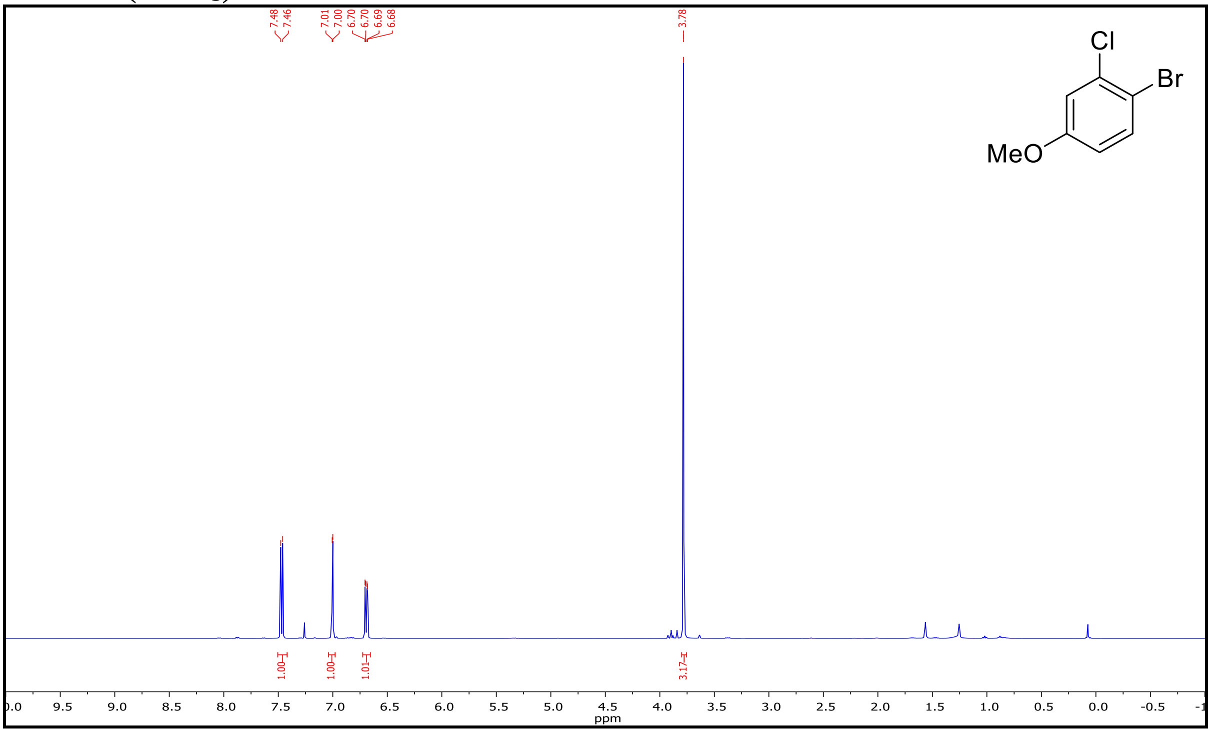

^{13}C NMR ($CDCl_3$)

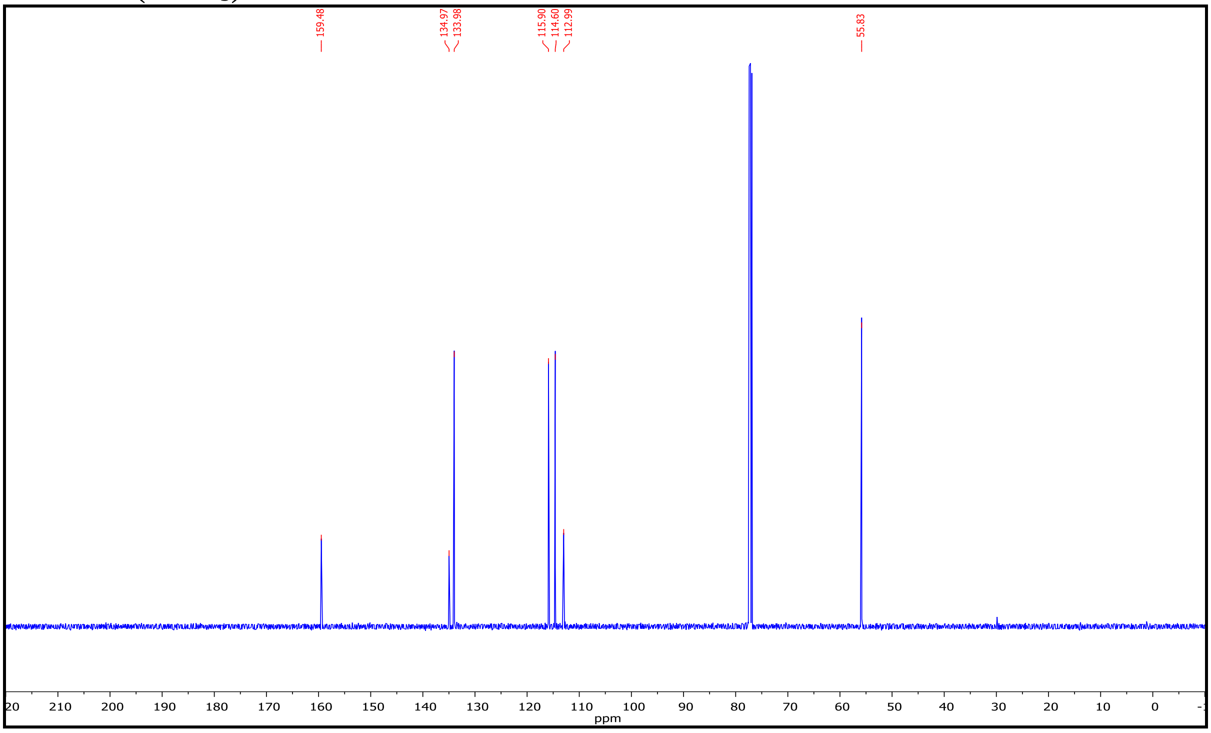

1,2-dibromo-4-methoxybenzene (2x)

^{1}H NMR ($CDCl_3$)

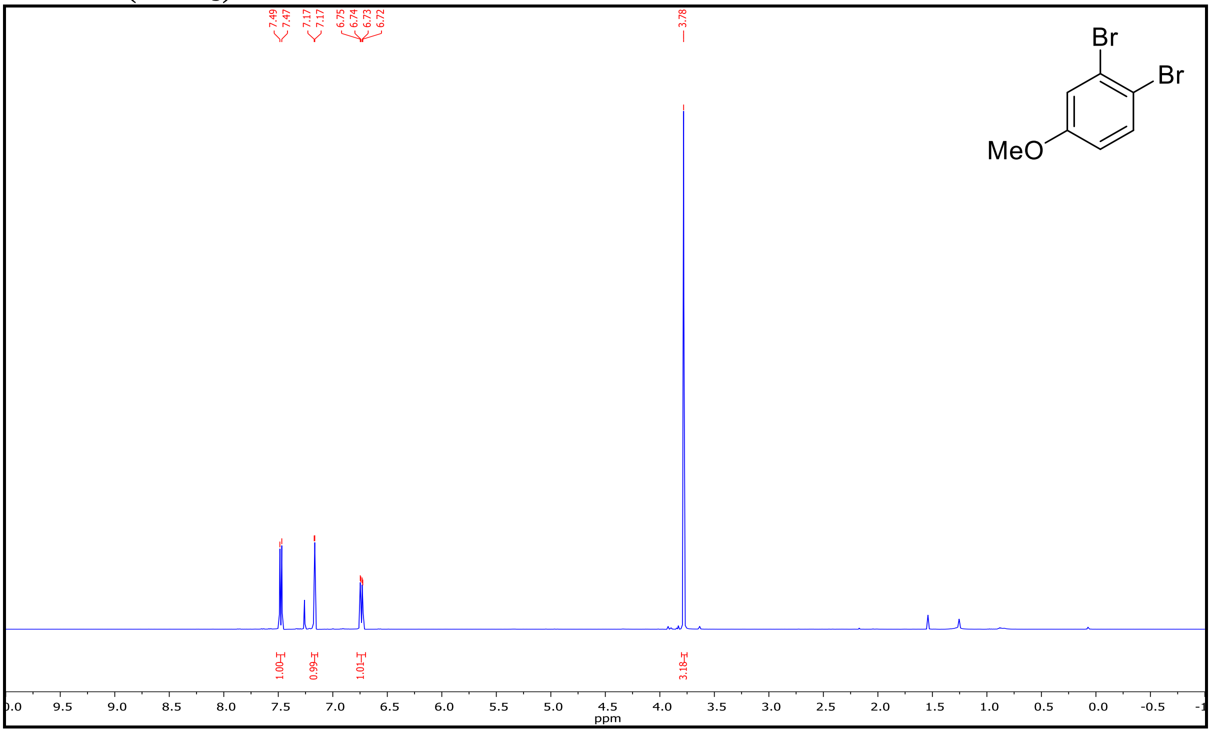

^{13}C NMR ($CDCl_3$)

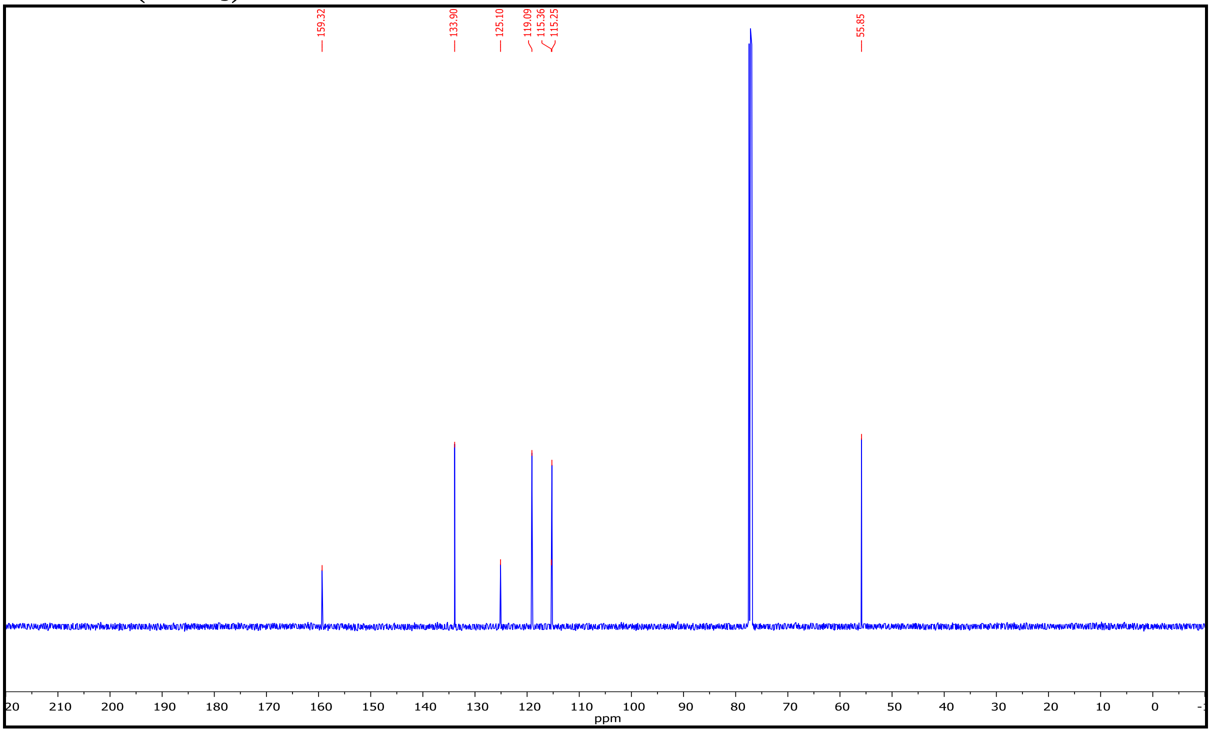

1-bromo-4-methoxy-2-(trifluoromethyl)benzene (2y)

^{1}H NMR (CDCl$_3$)

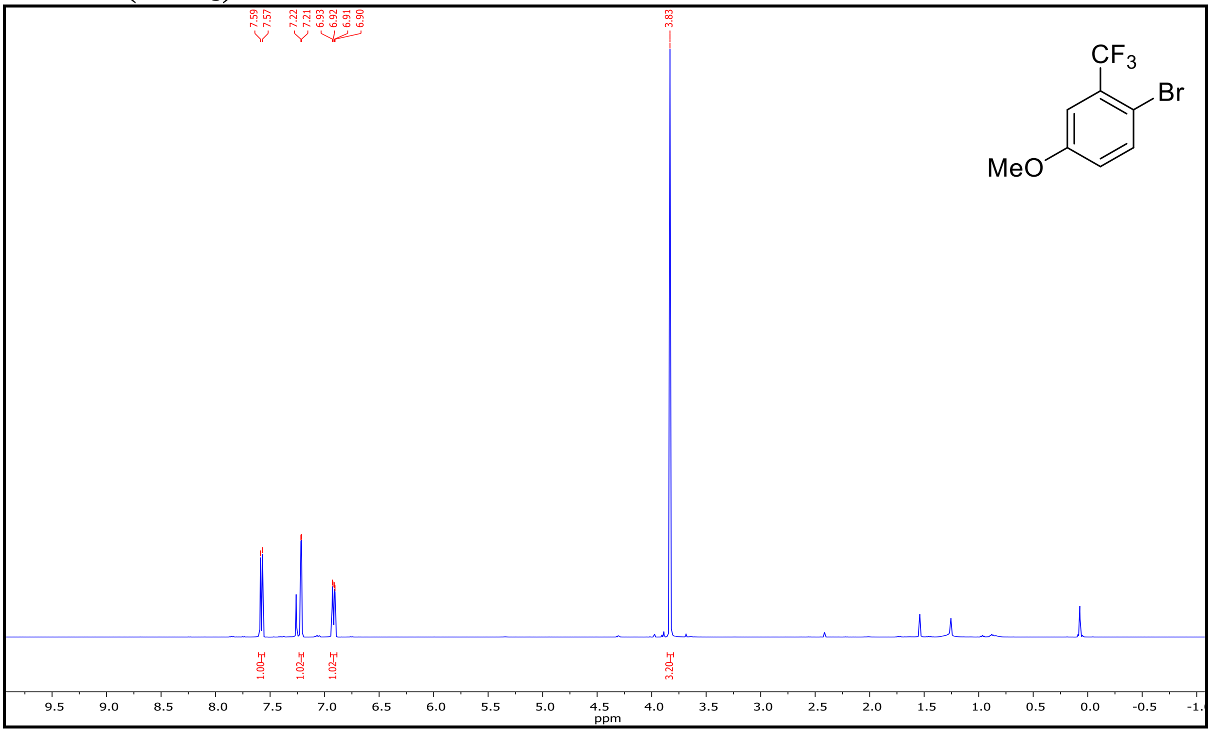

^{13}C NMR (CDCl$_3$)

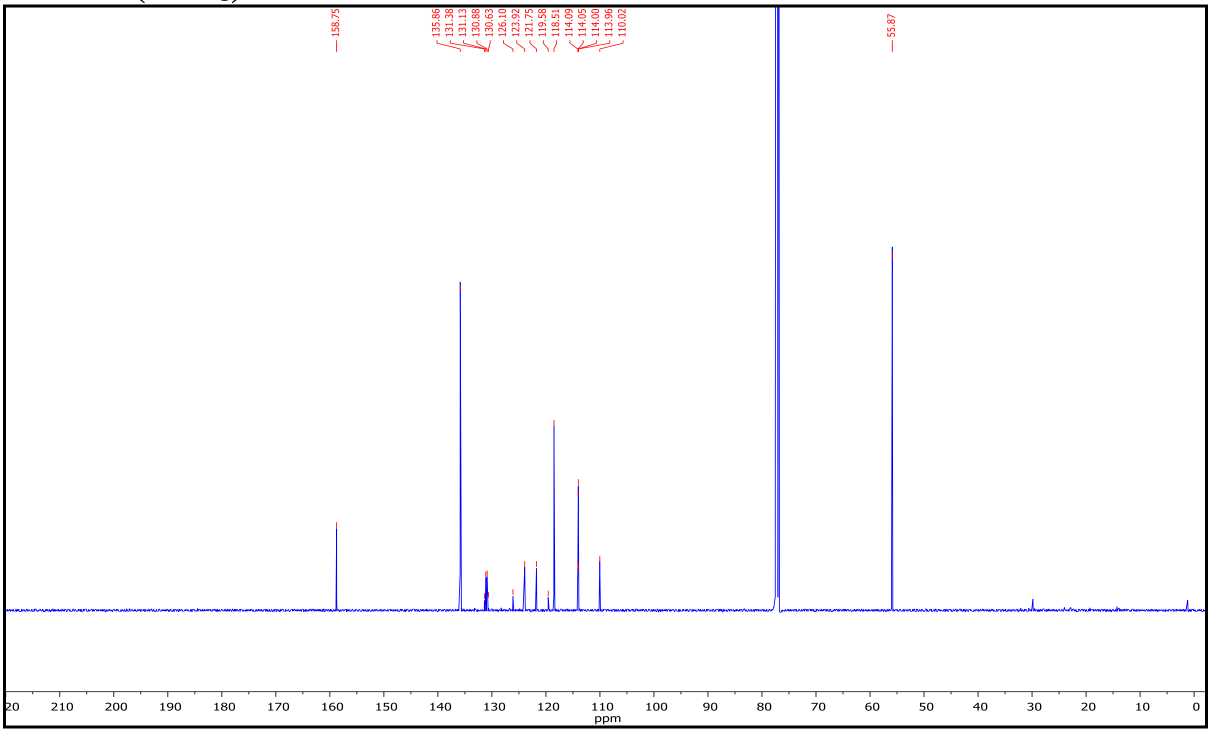

^{19}F NMR (CDCl$_3$)

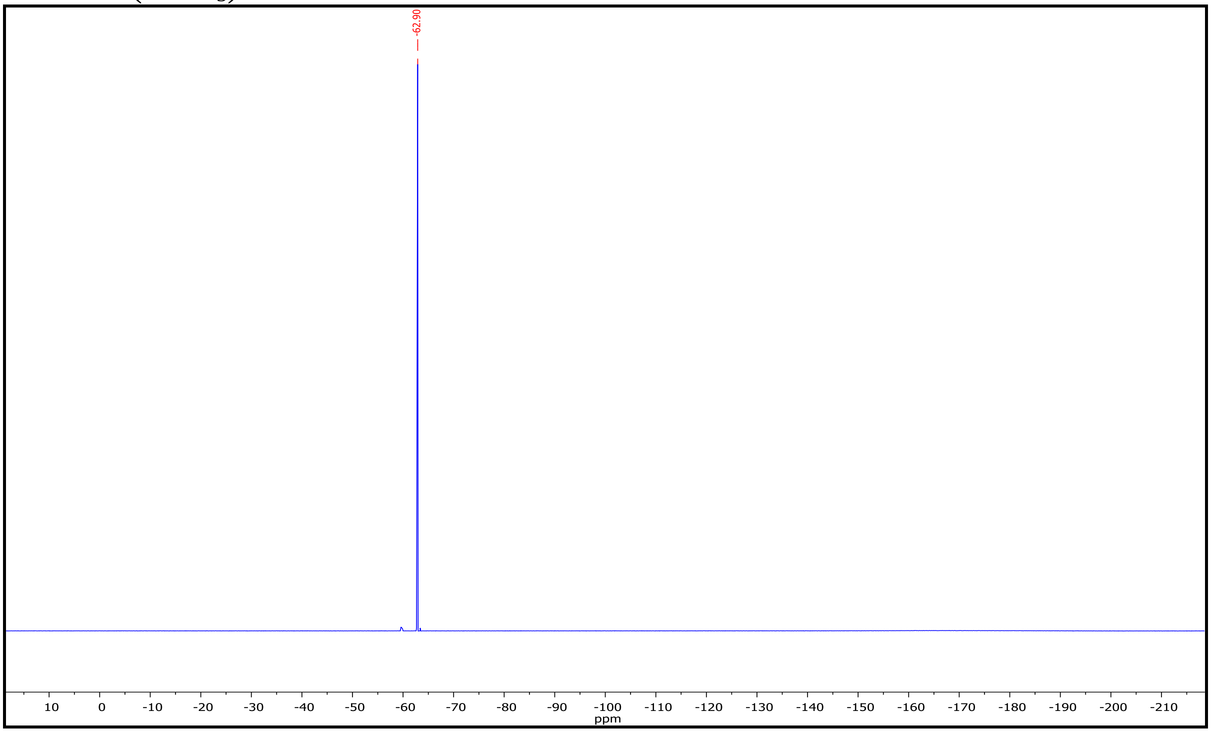

3-bromo-1-tosyl-1H-indole (4a)

^{1}H NMR ($CDCl_3$)

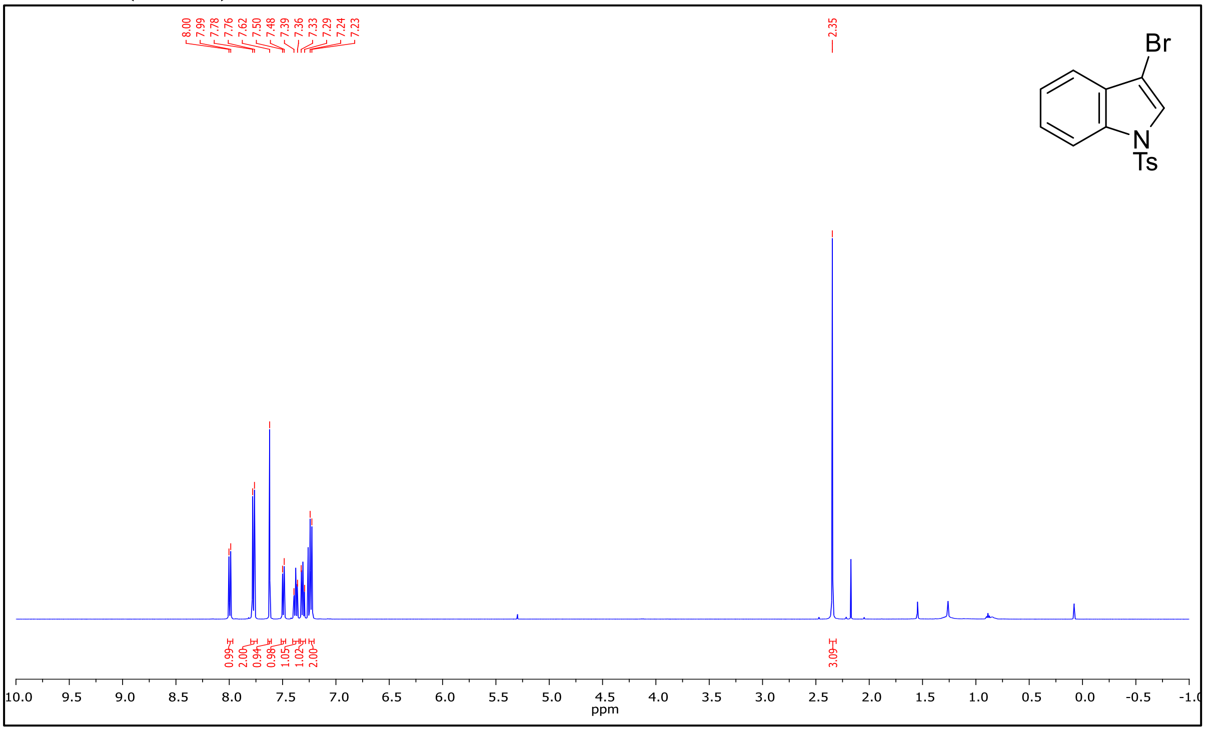

^{13}C NMR ($CDCl_3$)

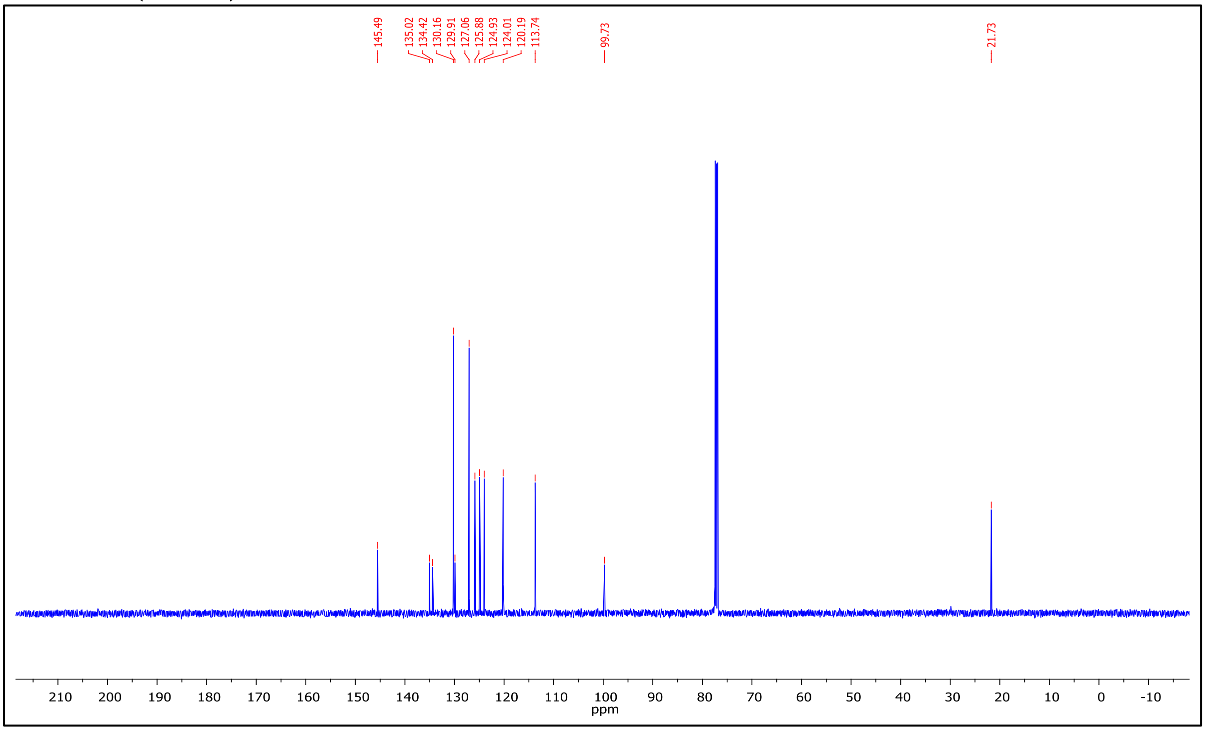

2-bromo-3-methylbenzo[b]thiophene (4b)

^{1}H NMR ($CDCl_3$)

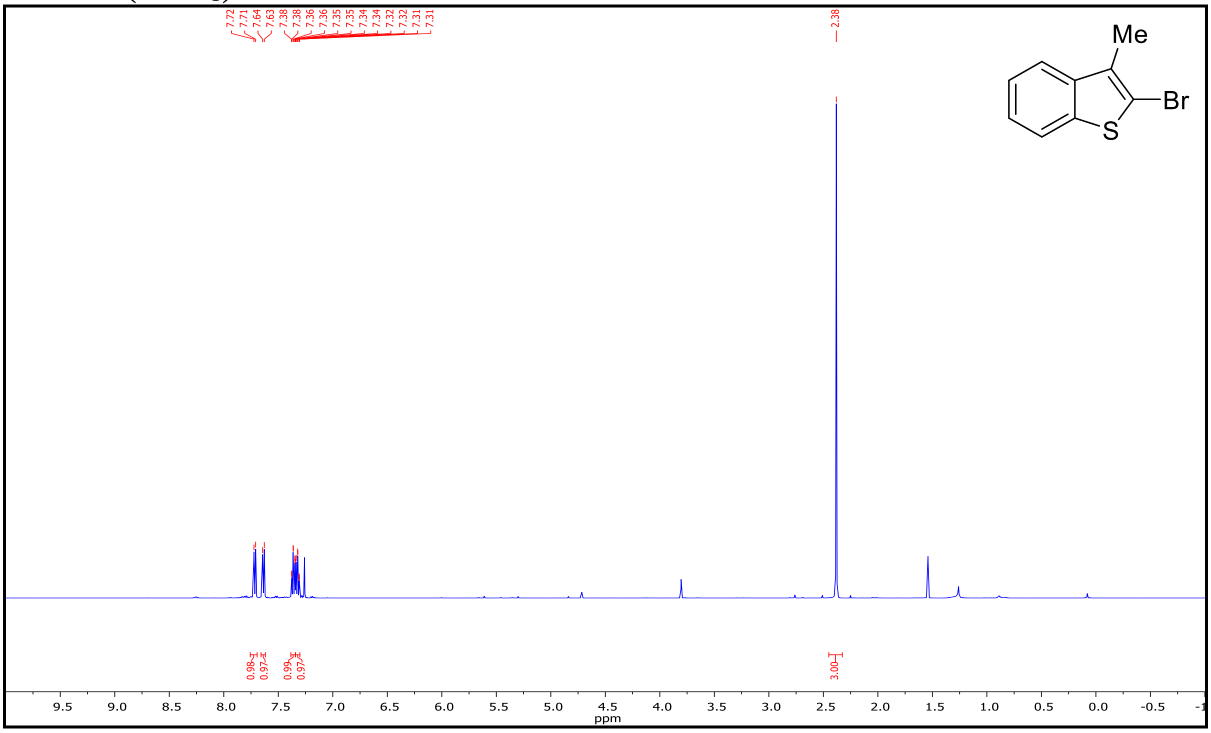

^{13}C NMR ($CDCl_3$)

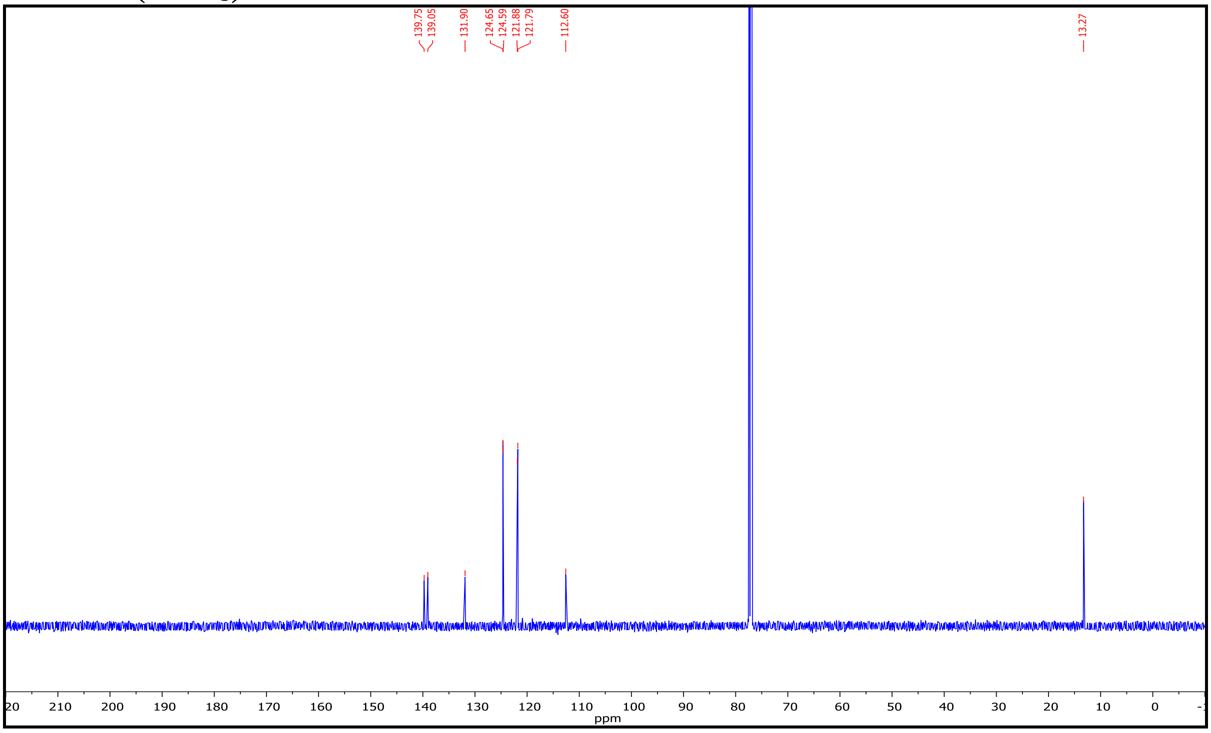

3-bromobenzo[b]thiophene (4c)

^{1}H NMR ($CDCl_3$)

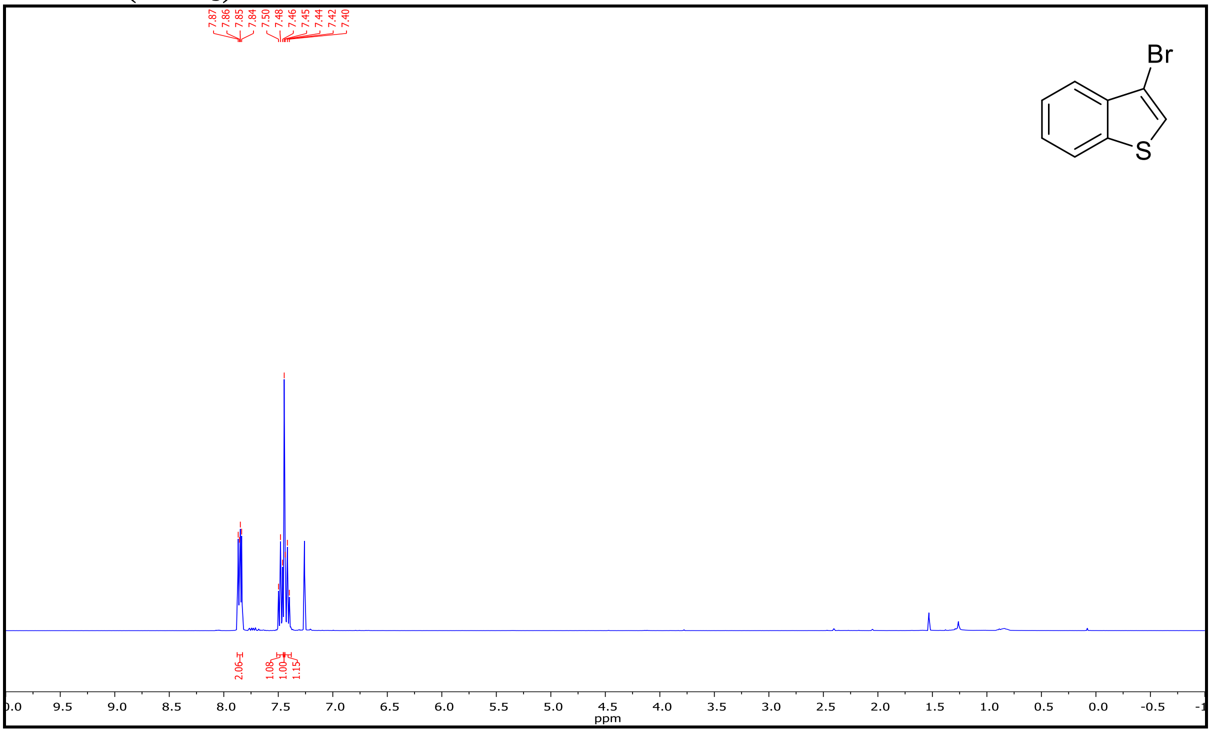

^{13}C NMR ($CDCl_3$)

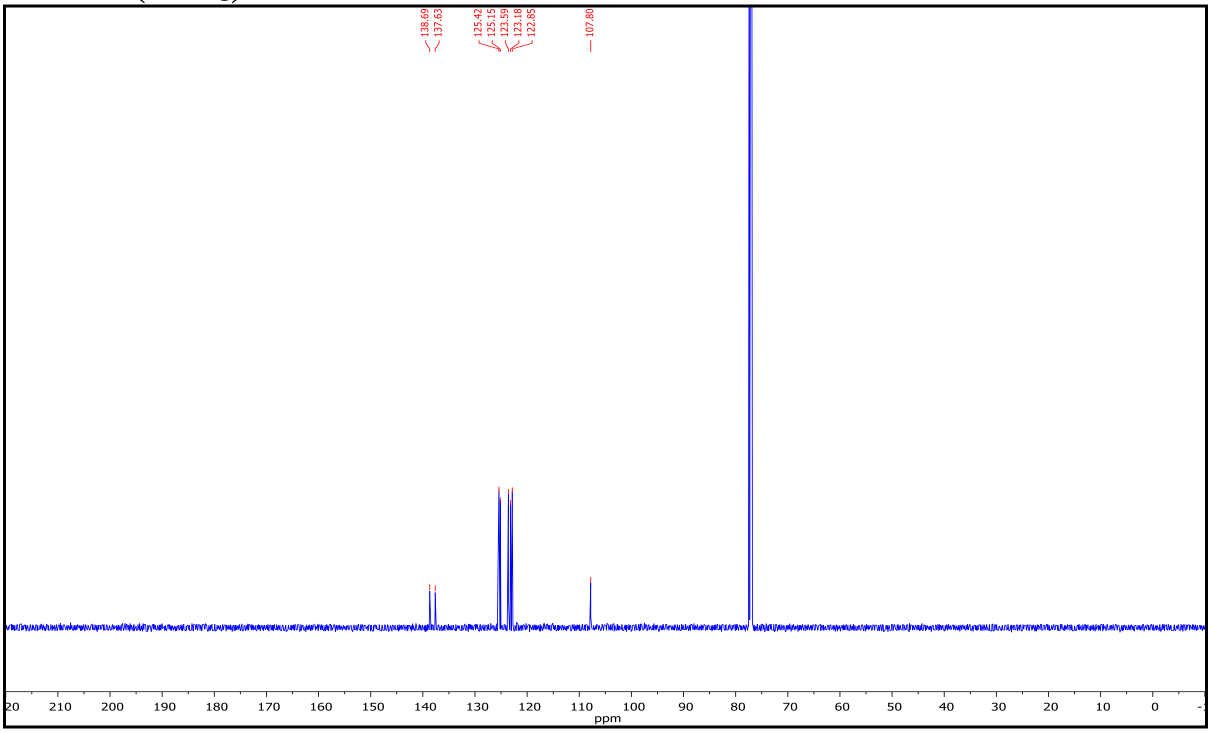

2-bromo-3-methylbenzofuran (4d)

^{1}H NMR ($CDCl_3$)

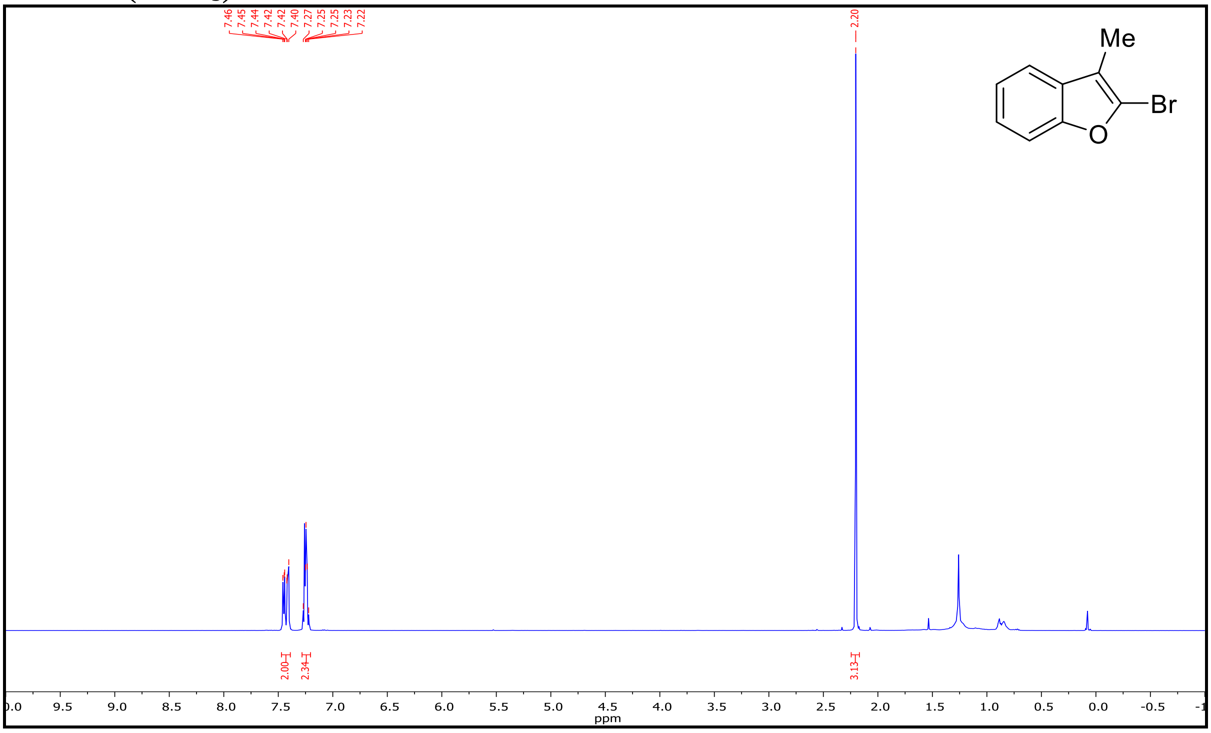

^{13}C NMR ($CDCl_3$)

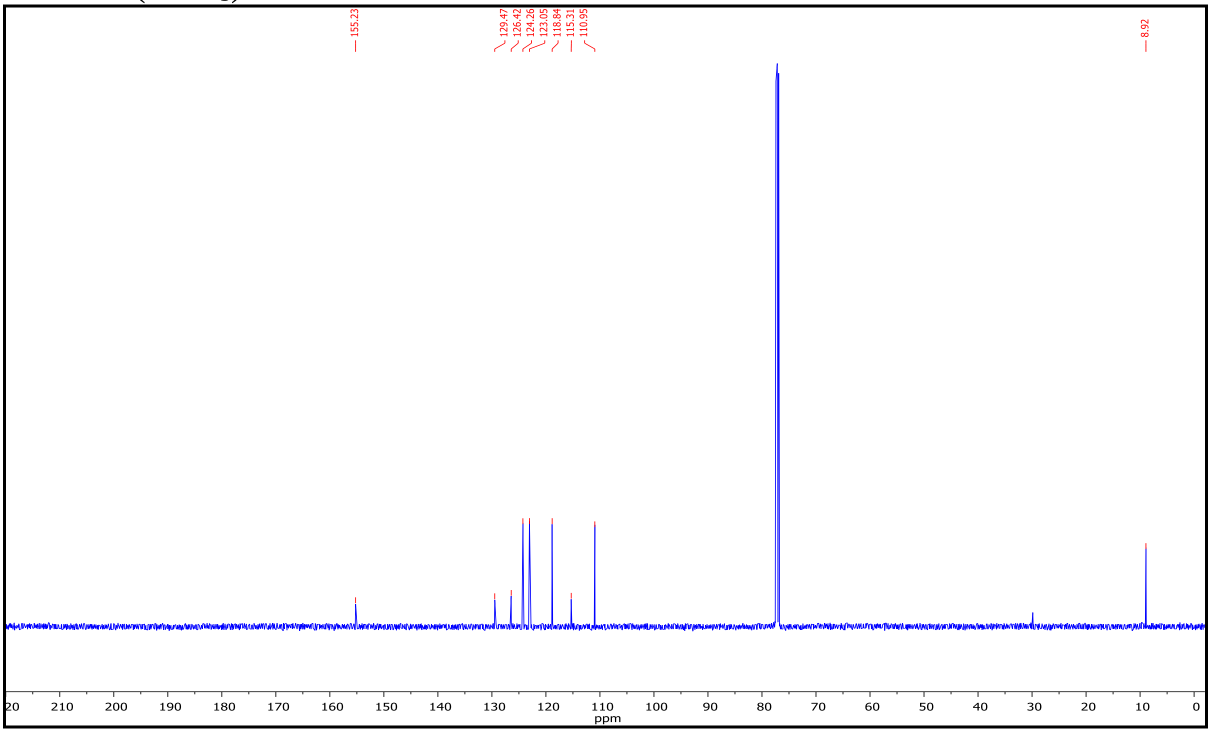

2-bromobenzo[b]thiophene (4e)

^{1}H NMR ($CDCl_3$)

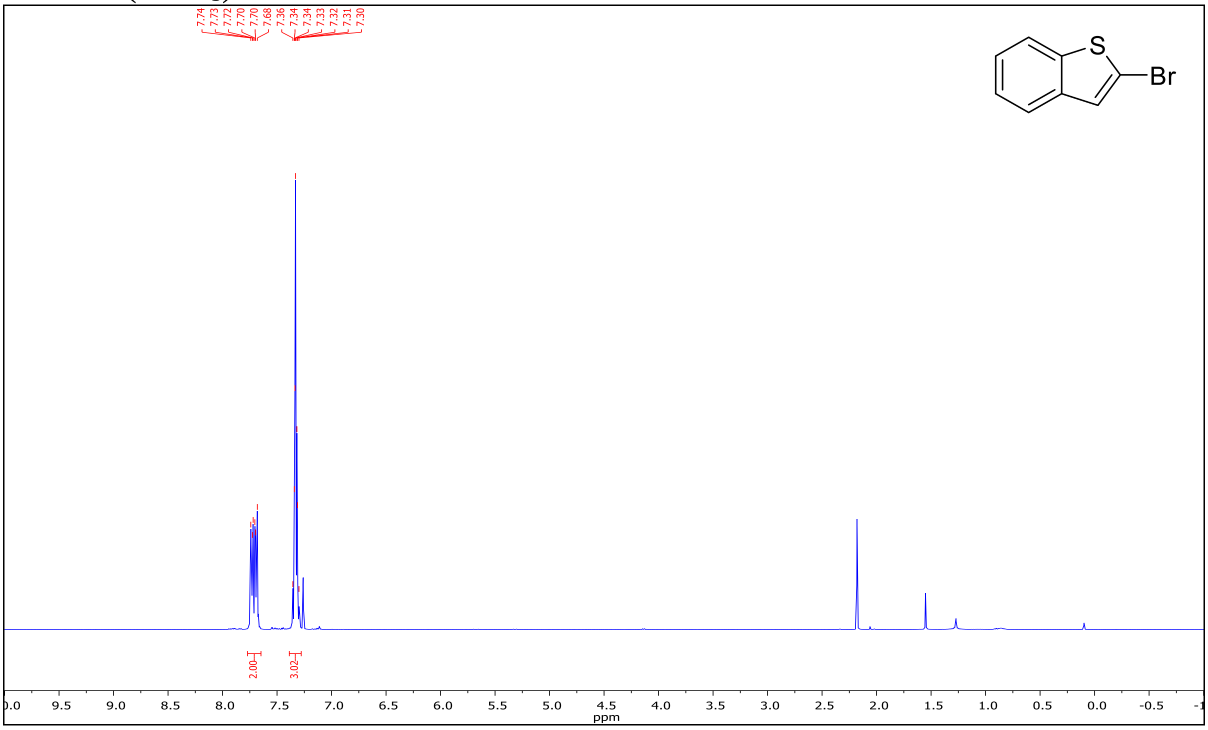

^{13}C NMR ($CDCl_3$)

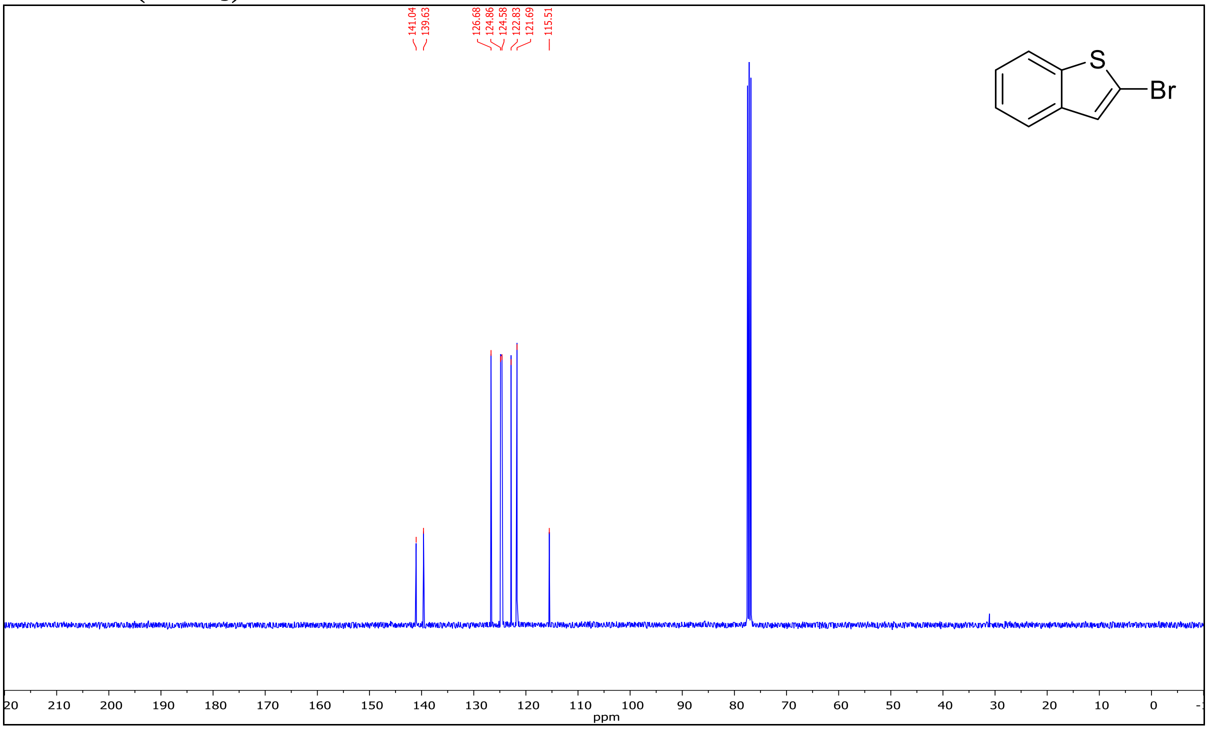

2,3-dibromobenzo[b]thiophene (4e')

^{1}H NMR ($CDCl_3$)

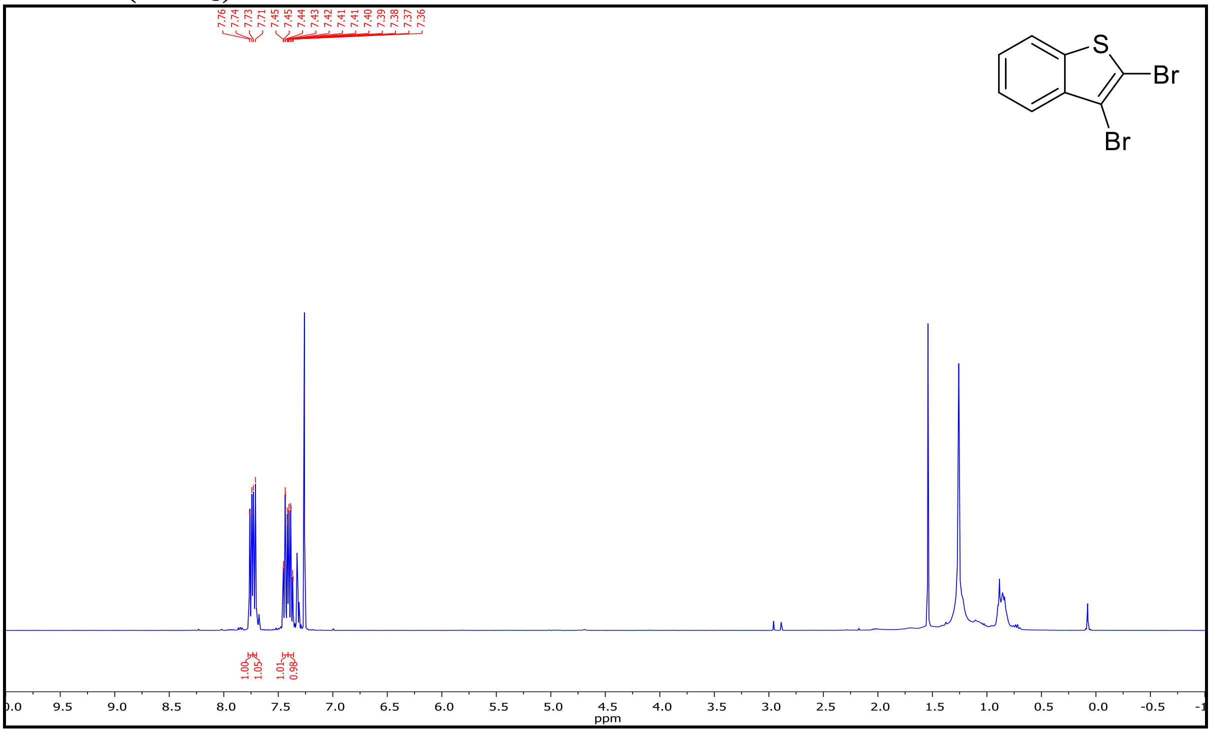

^{13}C NMR ($CDCl_3$)

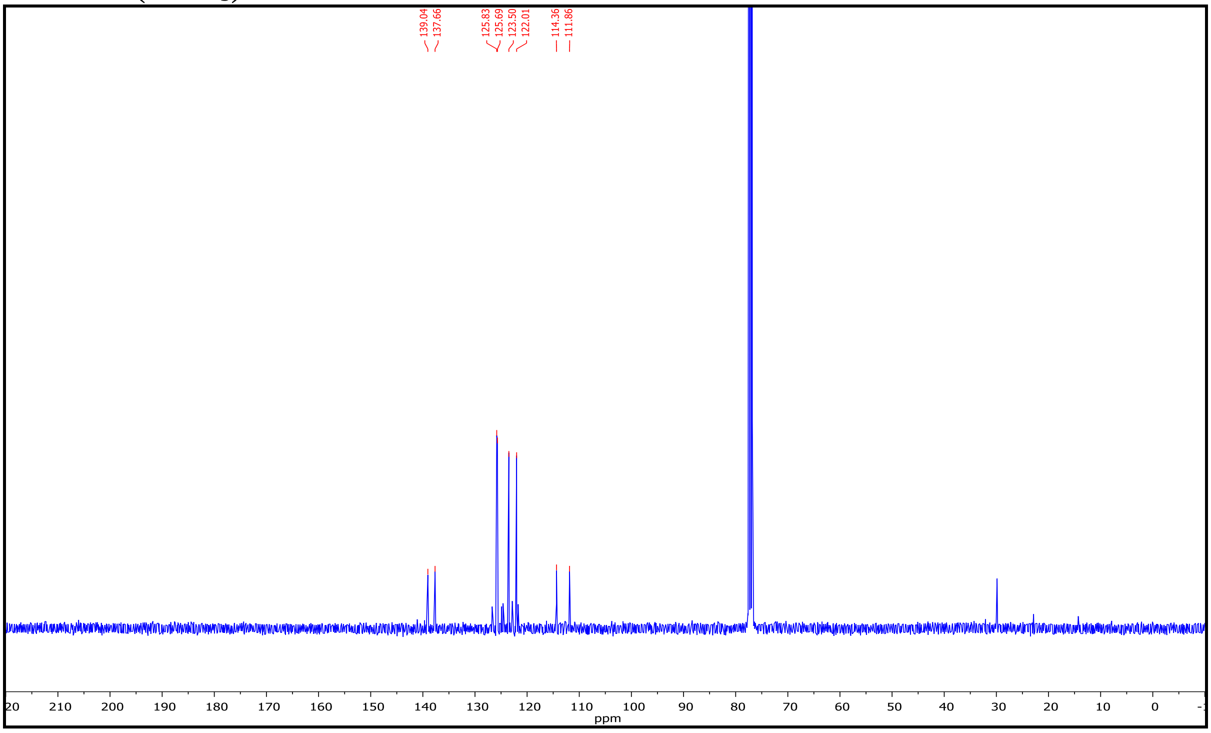

2-bromobenzofuran (4f)

^{1}H NMR ($CDCl_3$)

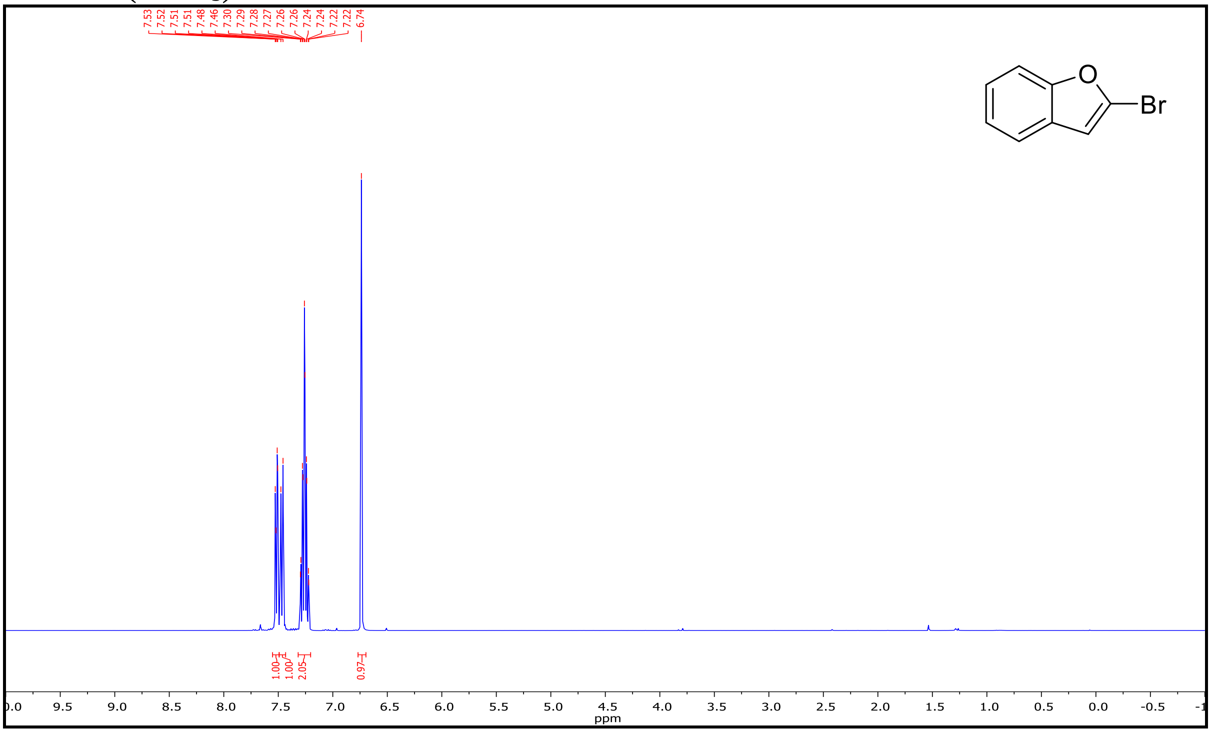

^{13}C NMR ($CDCl_3$)

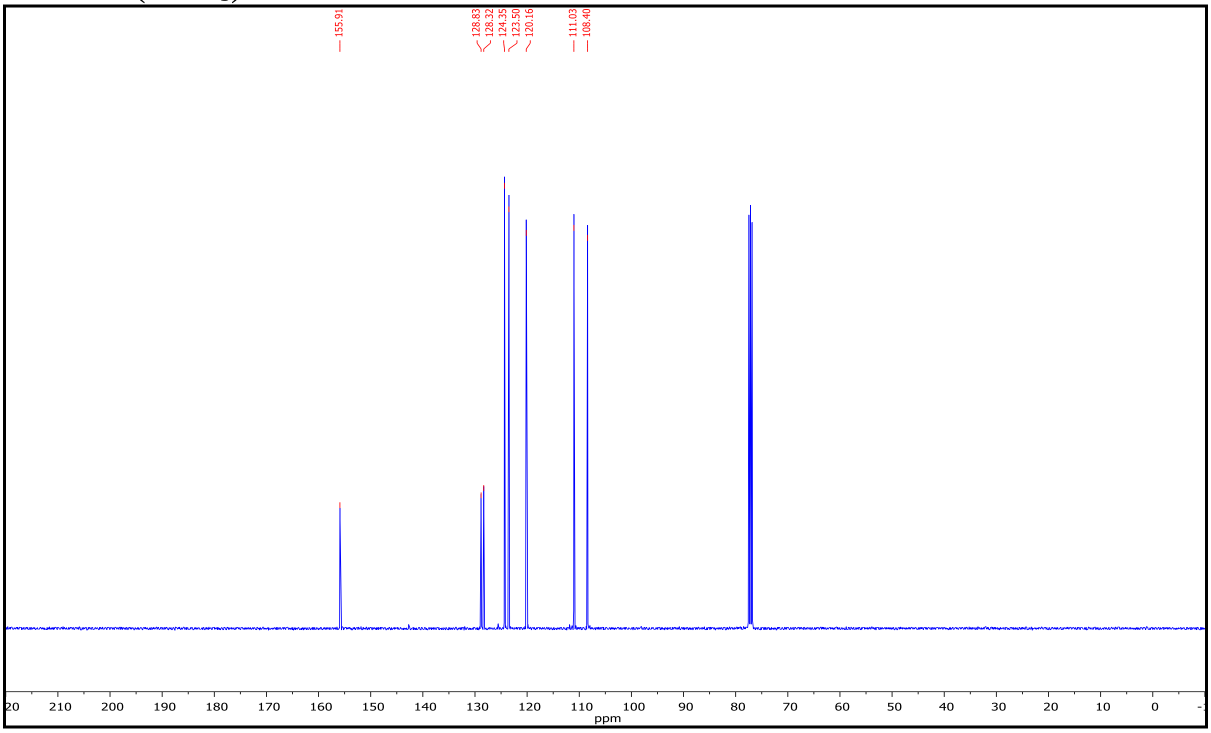

2,3-dibromobenzofuran (4f')

^{1}H NMR ($CDCl_3$)

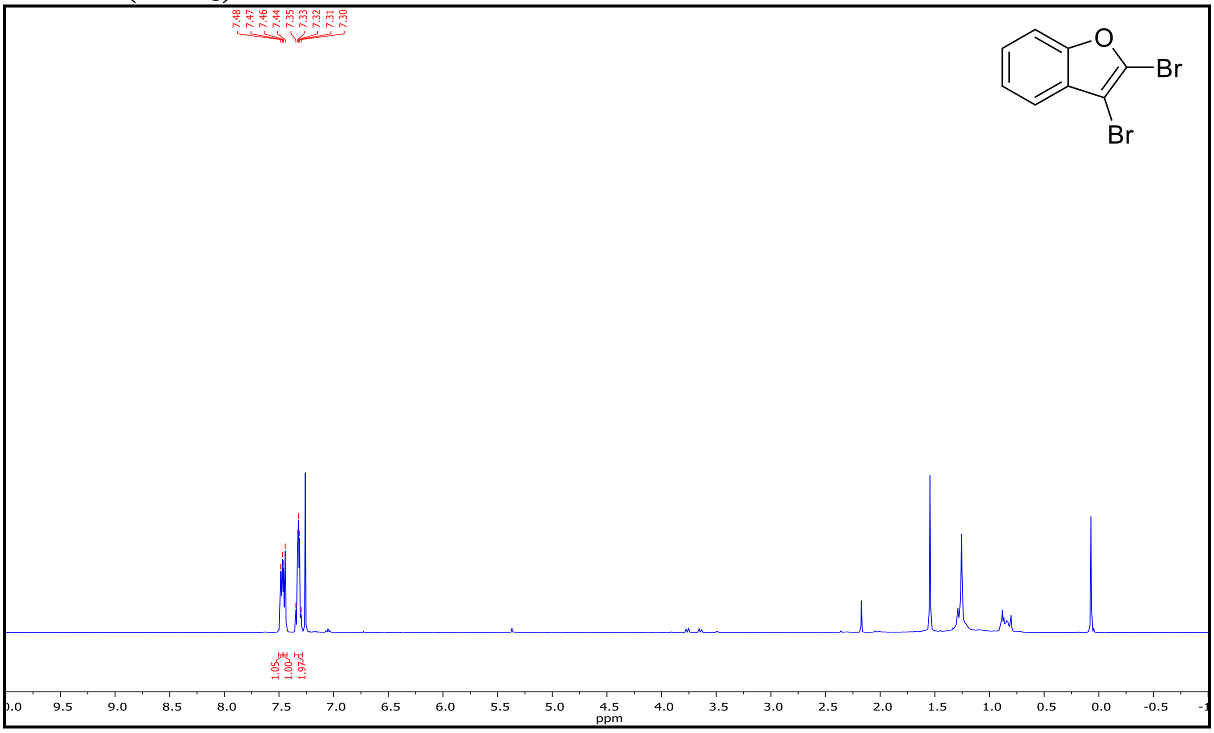

^{13}C NMR ($CDCl_3$)

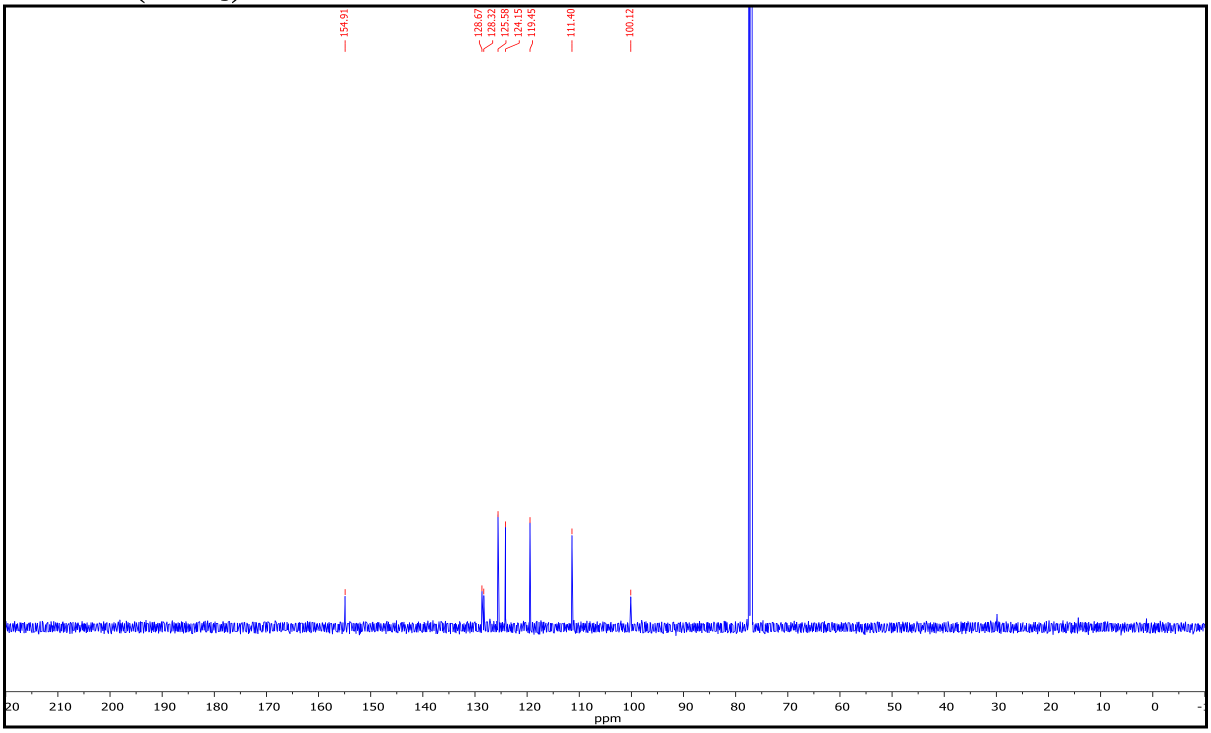

2,5-dibromothiophene (4g)

^{1}H NMR ($CDCl_3$)

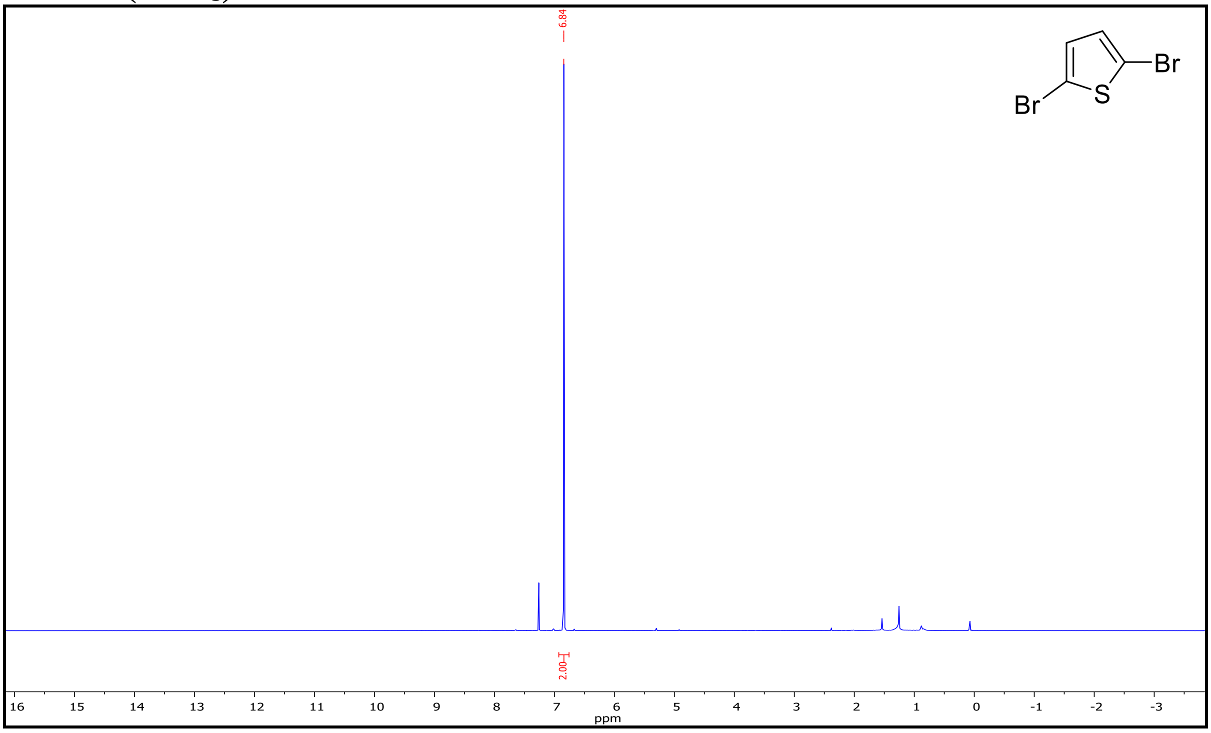

^{13}C NMR ($CDCl_3$)

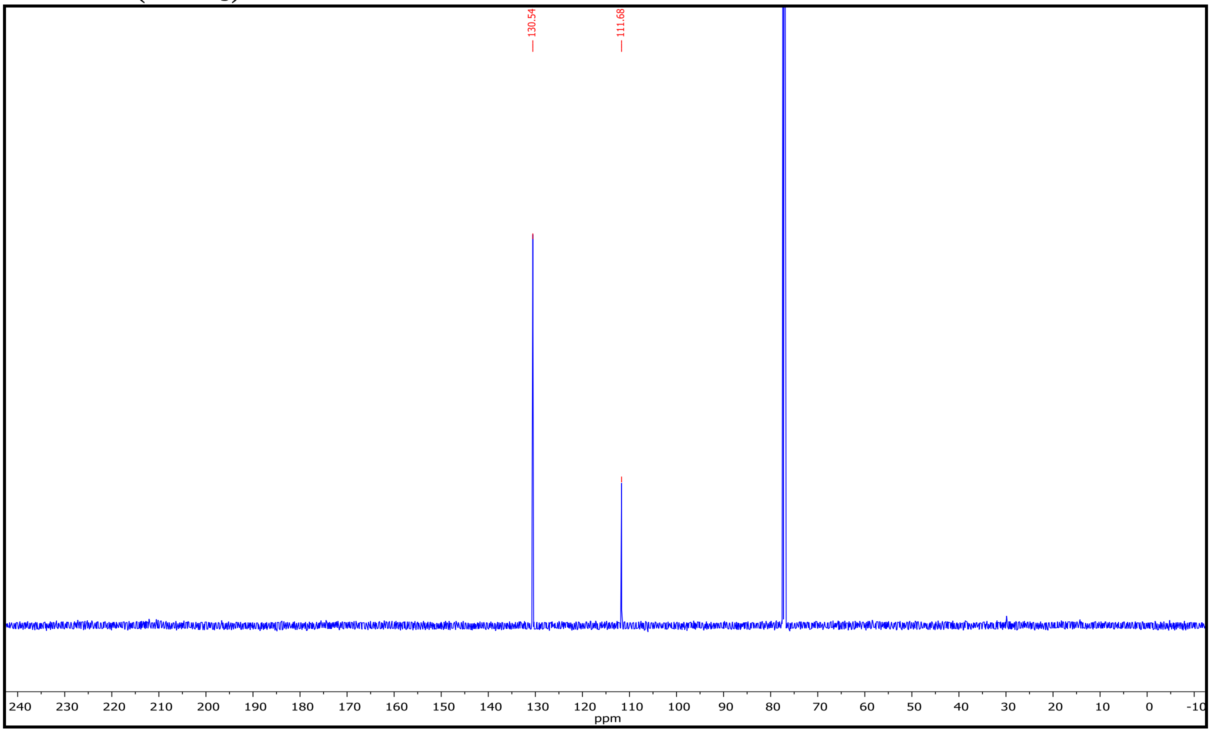

2-bromo-5-(p-tolyl)furan (4h)

^{1}H NMR ($CDCl_3$)

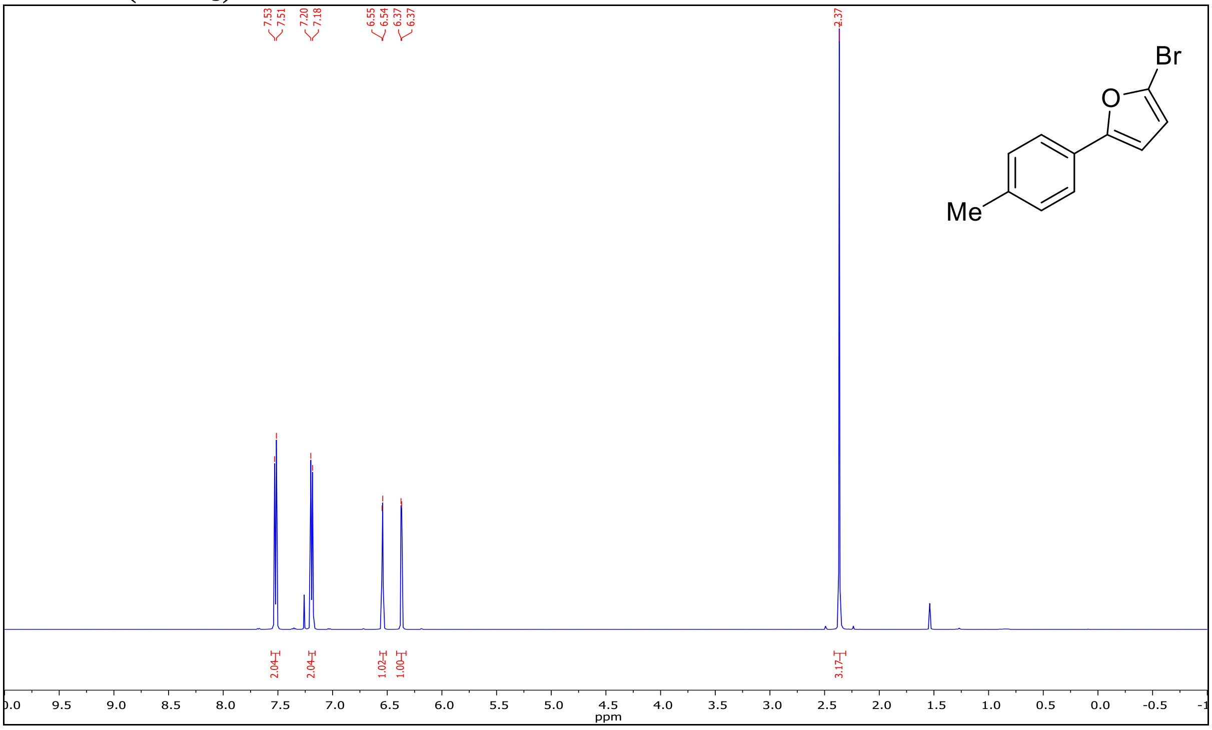

^{13}C NMR ($CDCl_3$)

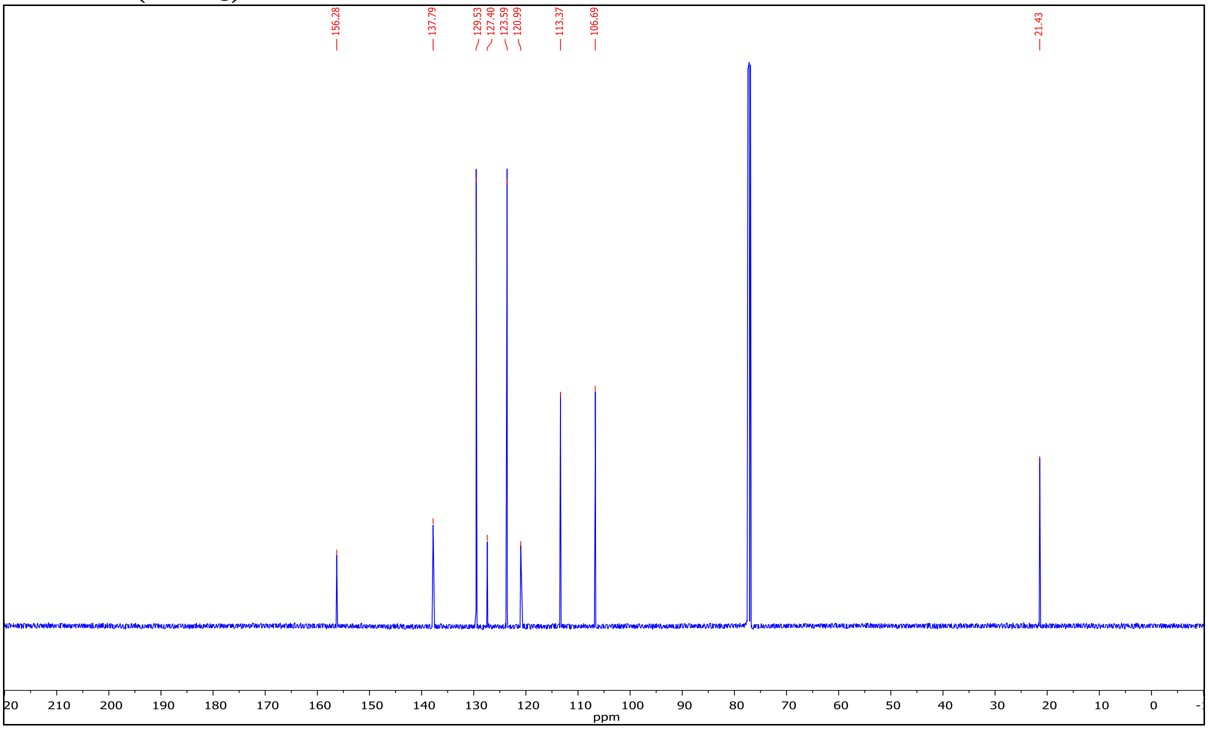

4-bromo-1-methyl-1H-pyrazole (4i)

^{1}H NMR ($CDCl_3$)

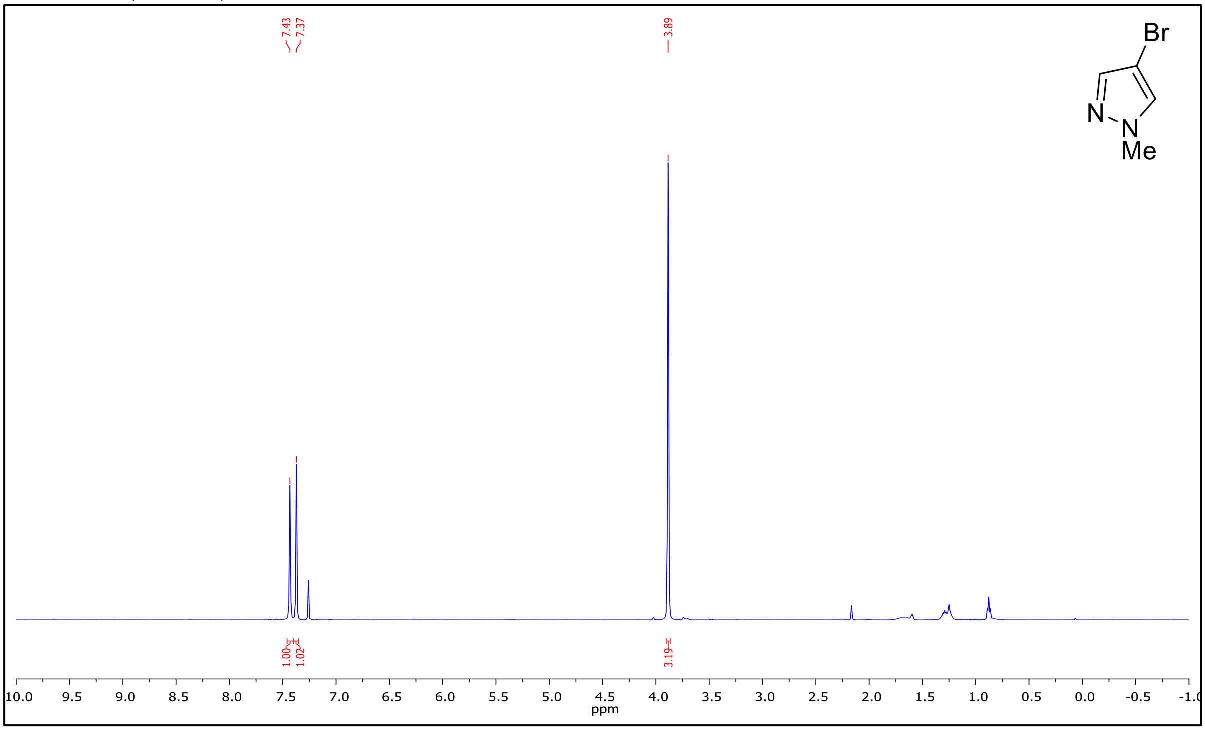

^{13}C NMR ($CDCl_3$)

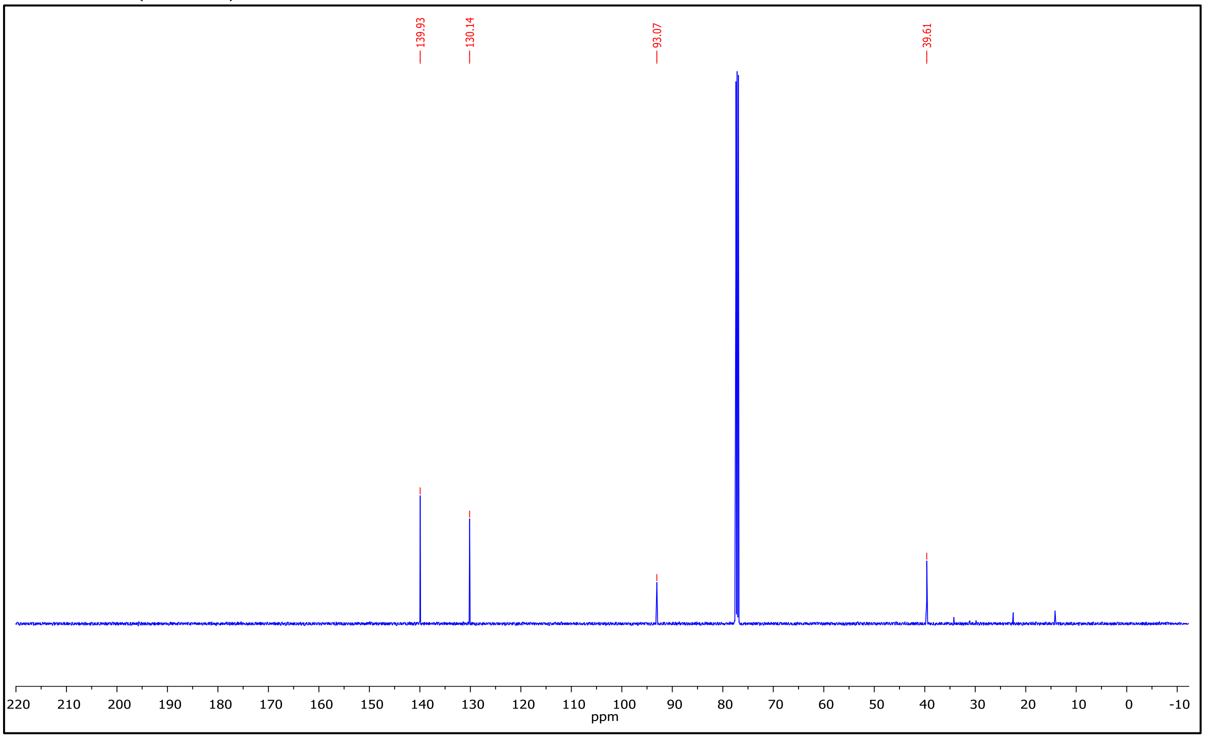

5-bromo-4-methylthiazole (4j)

^{1}H NMR ($CDCl_3$)

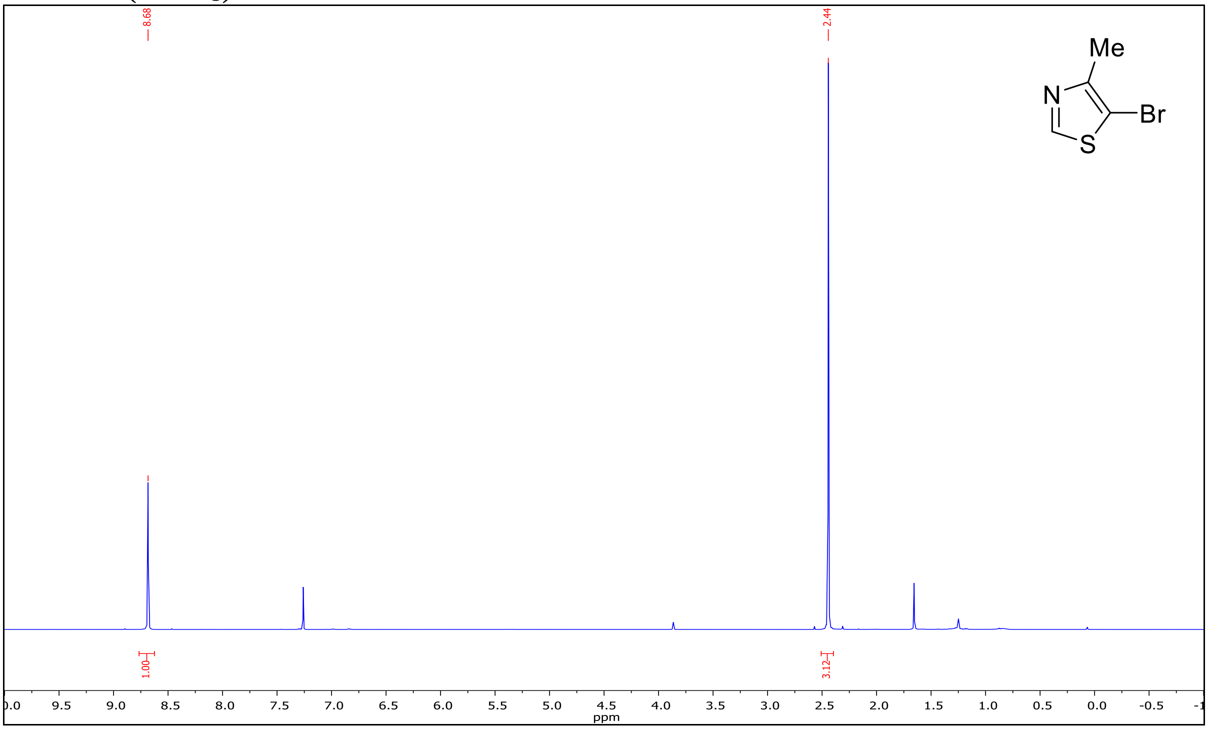

^{13}C NMR ($CDCl_3$)

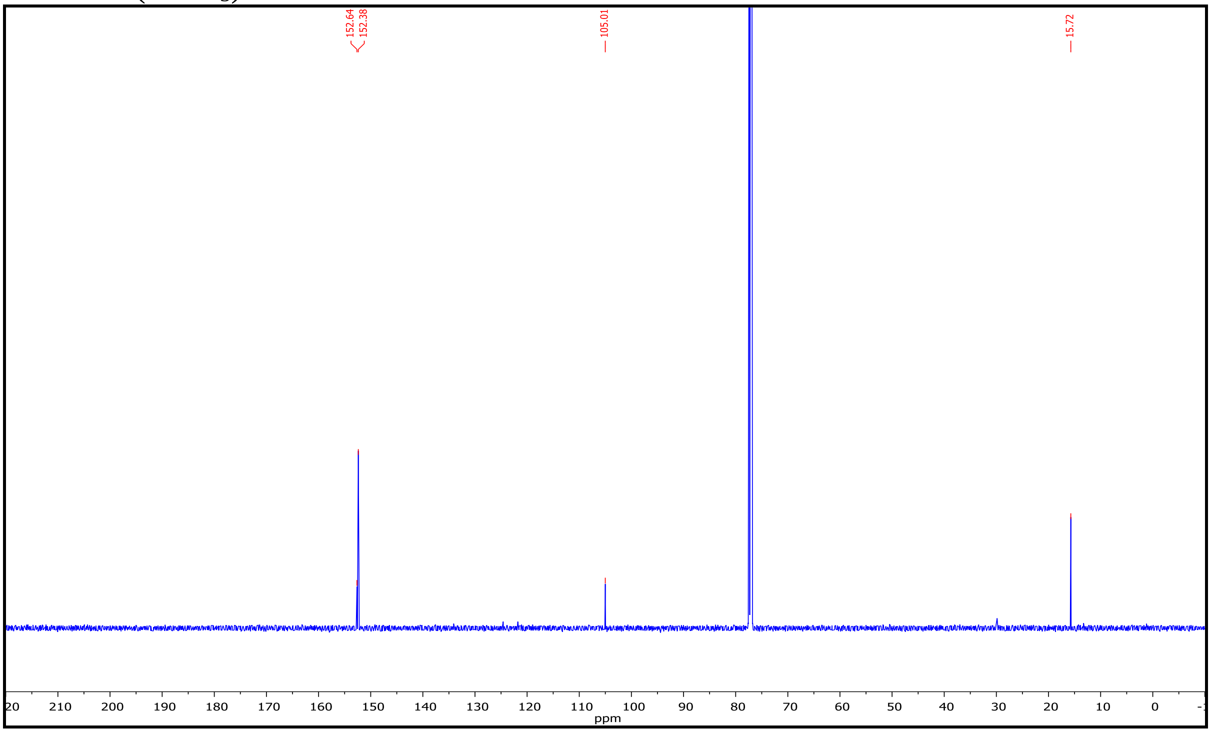

3-bromo-2-methoxypyridine (4k)

^{1}H NMR ($CDCl_3$)

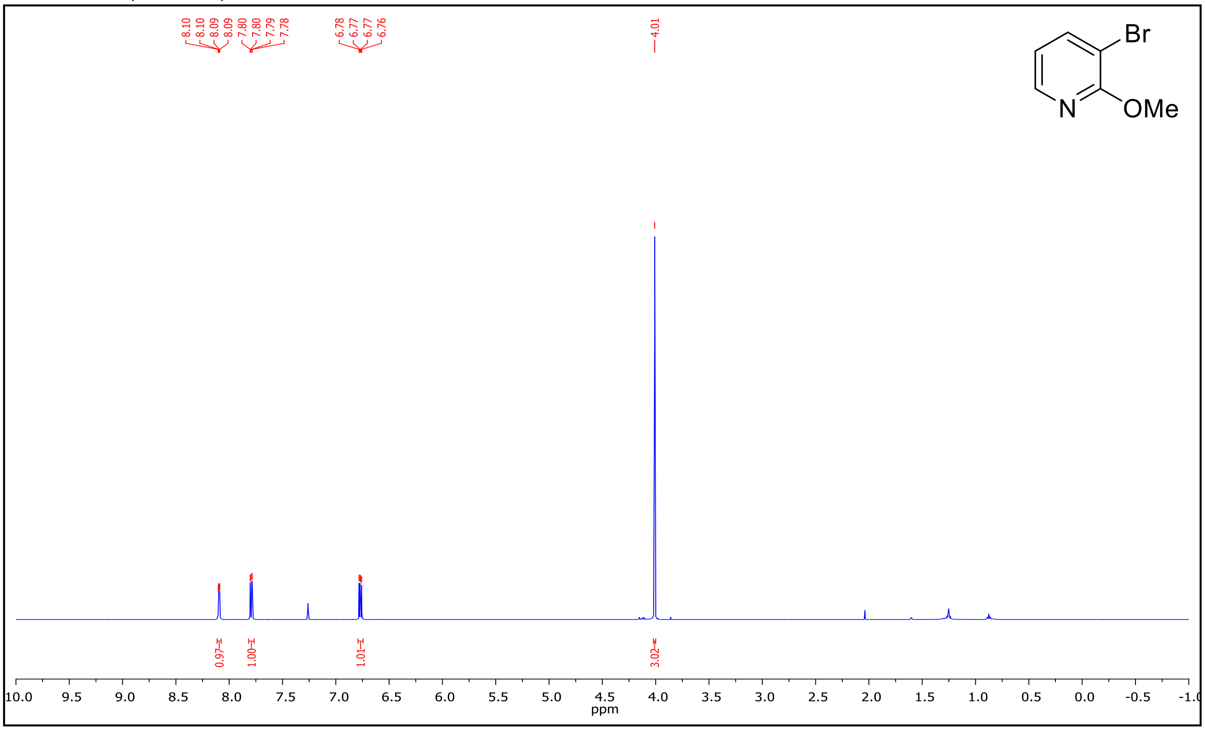

^{13}C NMR ($CDCl_3$)

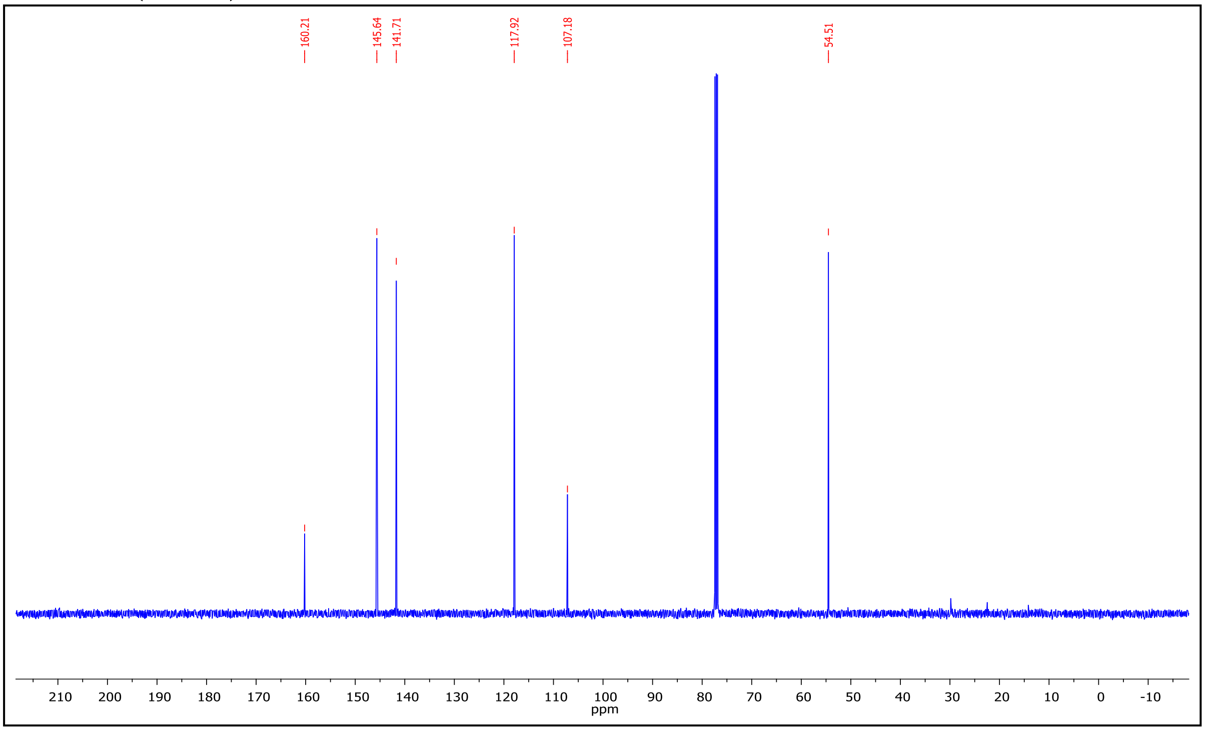

3-bromo-4H-chromen-4-one (4l)

^{1}H NMR ($CDCl_3$)

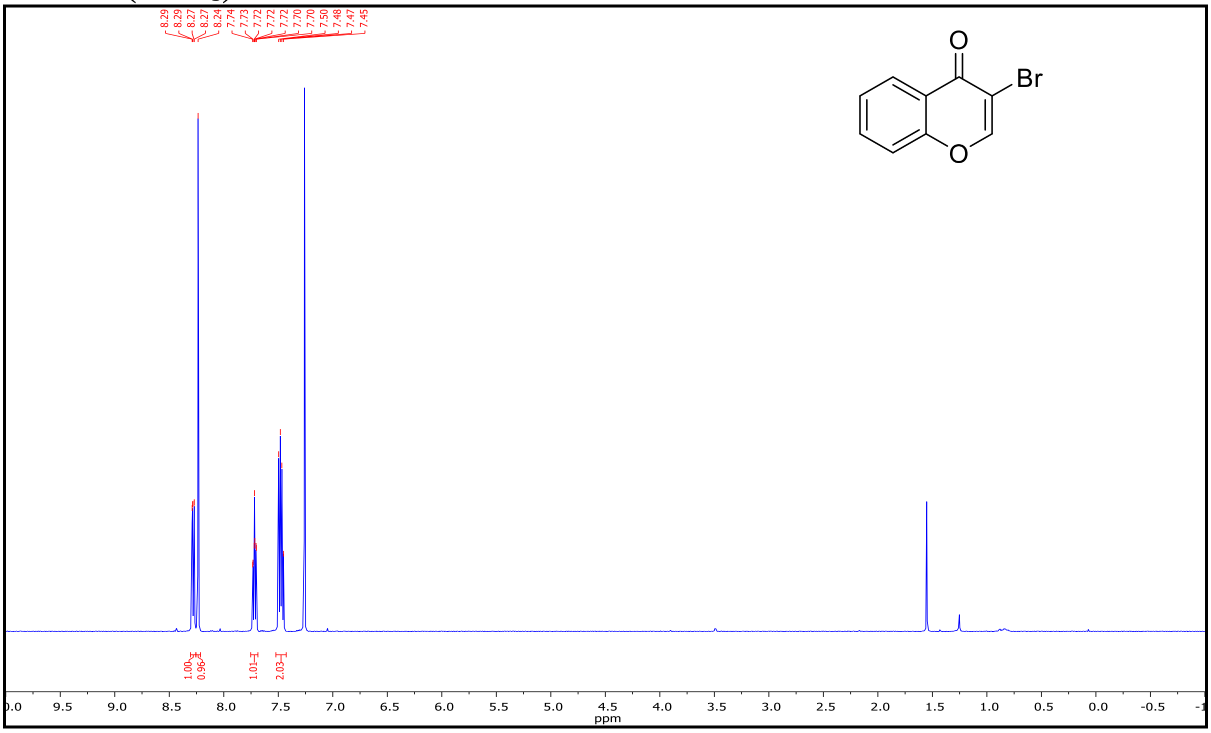

^{13}C NMR ($CDCl_3$)

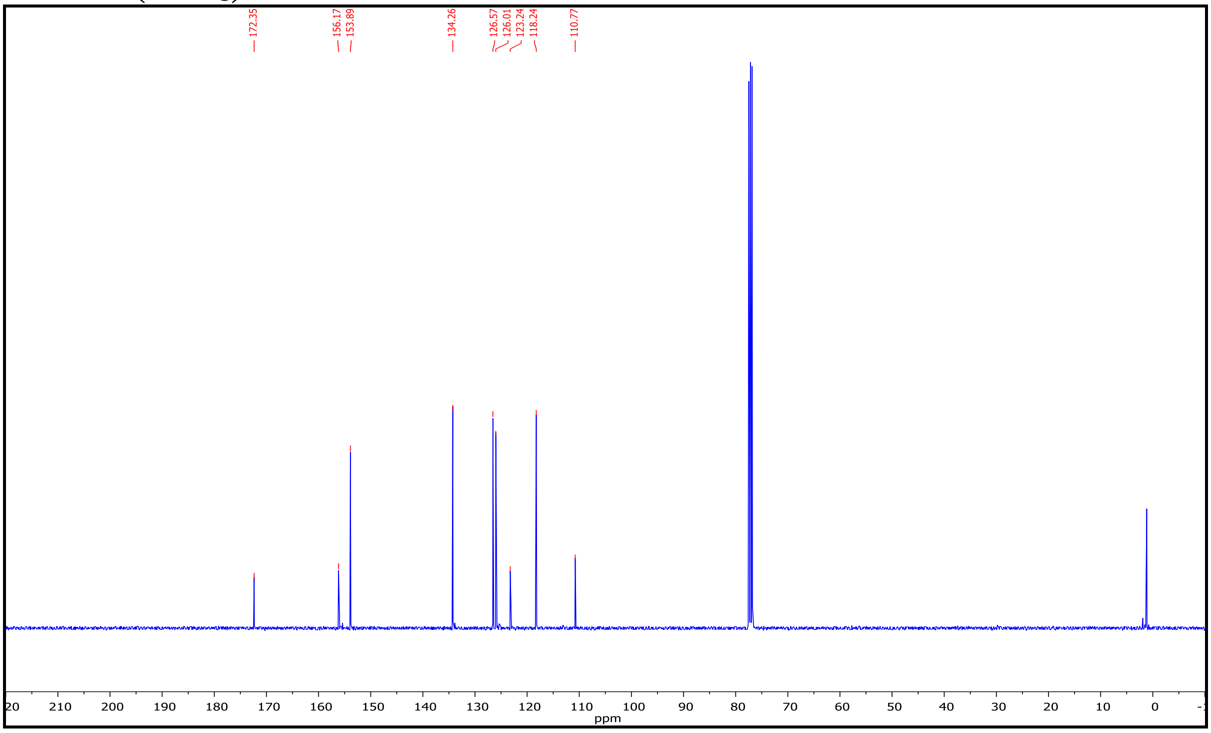

(E)-1-(2-bromovinyl)-4-methylbenzene (4m)

^{1}H NMR ($CDCl_3$)

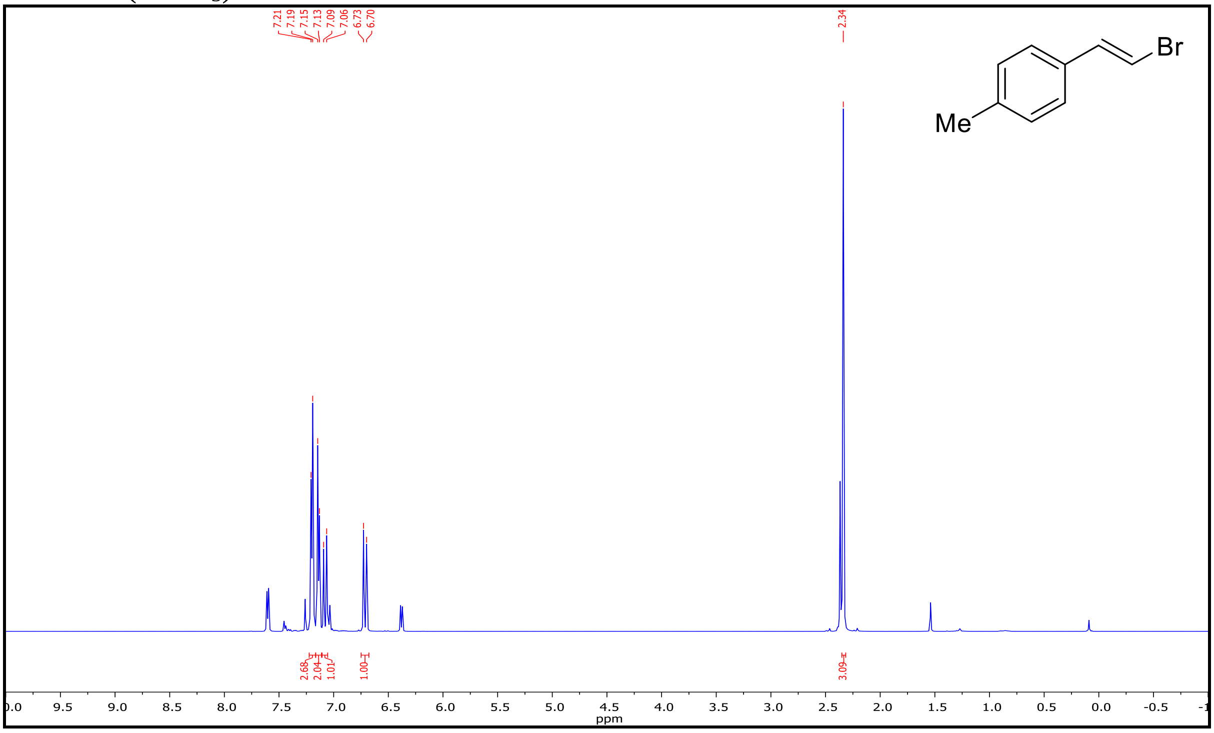

^{13}C NMR ($CDCl_3$)

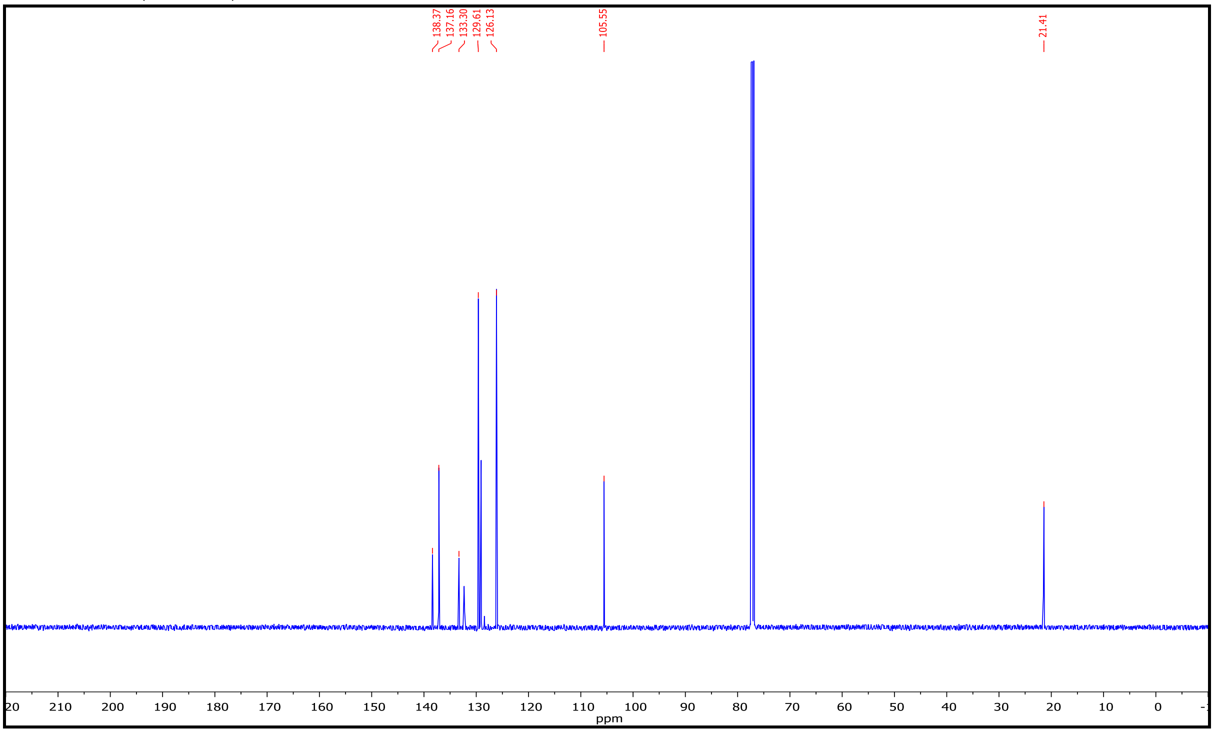

2-(allyloxy)-1-bromo-4-methoxybenzene (2A)

^{1}H NMR ($CDCl_3$)

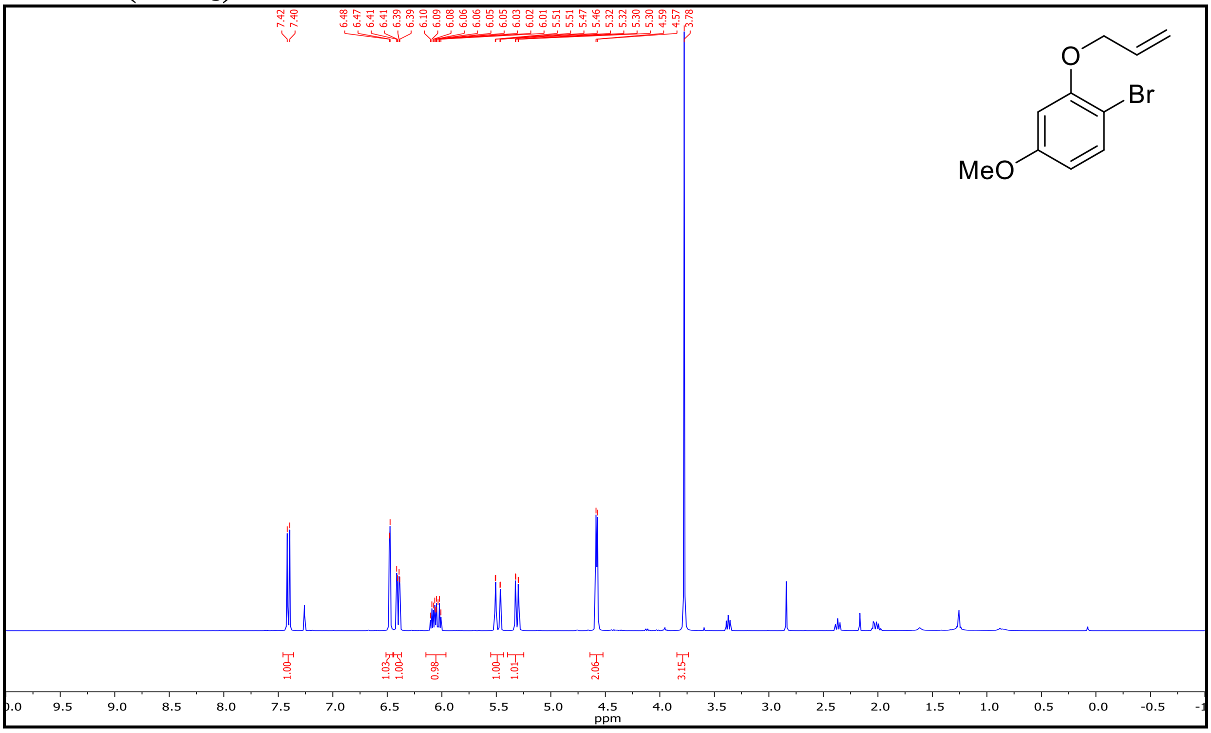

^{13}C NMR ($CDCl_3$)

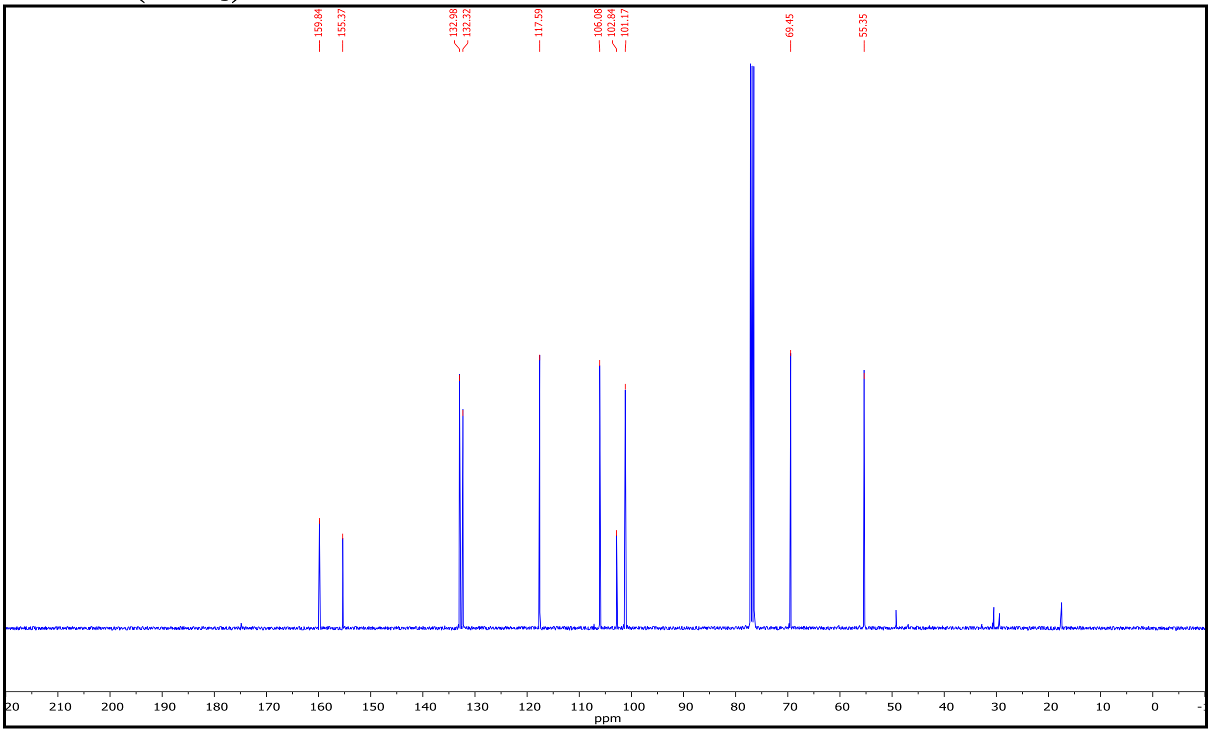

References

[1] G. J. P. Perry, J. M. Quibell, A. Panigrahi, I. Larrosa, *J. Am. Chem. Soc.* 2017, **139**, 11527–11536.
[2] S. J. Park, J. R. Price, M. H. Todd, *J. Org. Chem.* 2012, **77**, 949–955.
[3] Y. Imazaki, E. Shirakawa, R. Ueno, T. Hayashi, *J. Am. Chem. Soc.* 2012, **134**, 14760–14763.
[4] S. Song, X. Sun, X. Li, Y. Yuan, N. Jiao, *Org. Lett.* 2015, **17**, 2886–2889.
[5] Z. Fu, Z. Li, Y. Song, R. Yang, Y. Liu, H. Cai, *J. Org. Chem.* 2016, **81**, 2794–2803.
[6] P. Wucher, V. Goldbach, S. Mecking, *Organometallics*, 2013, **32**, 4516–4522.
[7] X. Xiong, F. Tan, Y.-Y. Yeung, *Org. Lett.* 2017, **19**, 4243–4246.
[8] R. Prebil, K. K. Laali, S. Stavber, *Org. Lett.* 2013, **15**, 2108–2111.
[9] X. Shen, A. M. Hyde, S. L. Buchwald, *J. Am. Chem. Soc.* 2010, **132**, 14076–14078.
[10] D. Feldman, M. Halpern, M. Rabinovitz, *J. Org. Chem.* 1985, **50**, 1746–1749.
[11] S. K. Sontag, J. A. Bilbrey, N. E. Huddleston, G. R. Sheppard, W. D. Allen, J. Locklin, *J. Org. Chem.* 2014, **79**, 1836–1841.
[12] (a) S. Orbisaglia, B. Jacques, P. Braunstein, D. Hueber, P. Pale, A. Blanc, P. de Frémont, *Organometallics*, 2013, **32**, 4153–4164. (b) W. Guo, E. Faggi, R. M. Sebastián, A. Vallribera, R. Pleixats, A. Shafir, *J. Org. Chem.*, 2013, **78**, 8169–8175.
[13] (a) H. Maeda, S. Matsuda, K. Mizuno, *J. Org. Chem.* 2016, **81**, 8544–8551. (b) M. P. Doyle, B. Siegfried, J. F. Dellaria, Jr., *J. Org. Chem.* 1977, **42**, 2426–2431.
[14] S. G. Ouellet, A. Bernardi, R. Angelaud, P. D. O'Shea, *Tetrahedron Lett.* 2009, **50**, 3776–3779.
[15] H. Kakinuma, T. Oi, Y. Hashimoto-Tsuchiya, M. Arai, Y. Kawakita, Y. Fukasawa, I. Iida, N. Hagima, H. Takeuchi, Y. Chino, J. Asami, L. Okumura-Kitajima, F. Io, D. Yamamoto, N. Miyata, T. Takahashi, S. Uchida, K. Yamamoto, *J. Med. Chem.* 2010, **53**, 3247–3261.
[16] D.-W. Lee, T. Kim, M. Lee, *Chem. Commun.* 2011, **47**, 8259–8261.
[17] M. Dabrowski, J. Kubicka, S. Lulliński, J. Serwatowski, *Tetrahedron*, 2005, **61**, 6590–6595.
[18] K. Iwanaga, J. Kobayashi, T. Kawashima, *Tetrahedron*, 2007, **63**, 10127–10132.
[19] M. A. B. Mostafa, R. M. Bowley, D. T. Racys, M. C. Henry, A. Sutherland, *J. Org. Chem.* 2017, **82**, 7529–7537.
[20] B. Akgun, D. G. Hall, *Angew. Chem. Int. Ed.* 2016, **55**, 3909–3913.
[21] P. K. Pramanick, Z.-L. Hou, B. Yao, *Tetrahedron*, 2017, **73**, 7105–7114.
[22] R. Bolton, E. S. E. Owen, *J. Fluorine Chem.* 1990, **46**, 393–406.
[23] B. Banks, M. R. Cargill, G. Sandford, A. J. Tadeusiak, H. Westemeier, D. S. Yufit, J. A. K. Howard, P. Kilickiran, G. Nelles, *J. Fluorine Chem.* 2010, **131**, 627–634.
[24] C. Douvris, C. M. Nagaraja, C.-H. Chen, B. M. Foxman, O. V. Ozerov, *J. Am. Chem. Soc.* 2010, **132**, 4946–4953.
[25] T. Abe, T. Suzuki, M. Anada, S. Matsunaga, K. Yamada, *Org. Lett.* 2017, **19**, 4275–4278.
[26] C. O. Hayes, W. K. Bell, B. R. Cassidy, C. G. Willson, *J. Org. Chem.* 2015, **80**, 7530–7535.
[27] S. G. Newman, V. Aureggi, C. S. Bryan, M. Lautens, *Chem. Commun.* 2009, 5236–5238.
[28] M. L. N. Rao, J. B. Talode, V. N. Murty, *Beilstein J. Org. Chem.* 2016**,** **12**, 2065–2076.
[29] D. D. Kenning, K. A. Mitchell, T. R, Calhoun, M. R. Funfar, D. J. Sattler, S. C. Rasmussen, *J. Org. Chem.* 2002, **67**, 9073–9076.
[30] O. S. Kim, J. H. Jang, H. T. Kim, S. J. Han, G. C. Tsui, J. M. Joo, *Org. Lett.* 2017, **19**, 1450–1453.
[31] Z. Chen, C. Hamdouchi, E. Hembre, P. Hipskind, J. Myers, T. Takakuwa, J. Toth, Patent Number: WO2008/36579. 2008.
[32] M.-J. Shiao, K.-Y. Tarng, *Heterocycles*, 1990, **31**, 819–824.
[33] K. Mal, A. Kaur, F. Haque, I. Das, *J. Org. Chem.* 2015, **80**, 6400–6410.
[34] J. J. Mousseau, J. A. Bull, C. L. Ladd, A. Fortier, D. S. Roman, A. B. Charette, *J. Org. Chem.*2011,**76**, 8243–8261.
[35] Gaussian 09, Revision D.01, M. J. Frisch, G. W. Trucks, H. B. Schlegel, G. E. Scuseria, M. A. Robb, J. R. Cheeseman, G. Scalmani, V. Barone, G. A. Petersson, H. Nakatsuji, X. Li, M. Caricato, A. Marenich, J. Bloino, B. G. Janesko, R. Gomperts, B. Mennucci, H. P. Hratchian, J. V. Ortiz, A. F. Izmaylov, J. L. Sonnenberg, D. Williams-Young, F. Ding, F. Lipparini, F. Egidi, J. Goings, B. Peng, A. Petrone, T. Henderson, D. Ranasinghe, V. G. Zakrzewski, J. Gao, N. Rega, G. Zheng, W. Liang, M. Hada, M. Ehara, K. Toyota, R. Fukuda, J. Hasegawa, M. Ishida, T. Nakajima, Y. Honda, O. Kitao, H. Nakai, T. Vreven, K. Throssell, J. A. Montgomery, Jr., J. E. Peralta, F. Ogliaro, M. Bearpark, J. J. Heyd, E. Brothers, K. N. Kudin, V. N. Staroverov, T. Keith, R. Kobayashi, J. Normand, K. Raghavachari, A. Rendell, J. C. Burant, S. S. Iyengar, J. Tomasi, M. Cossi, J. M. Millam, M. Klene, C. Adamo, R. Cammi, J. W. Ochterski, R. L. Martin, K. Morokuma, O. Farkas, J. B. Foresman, and D. J. Fox, Gaussian, Inc., Wallingford CT, 2016.

[36] D. A. Singleton, A. A. Thomas, *J. Am. Chem. Soc.* 1995, **117**, 9357–9358.
[37] L. Melander; W. H. Saunders, In *Reactions Rates of Isotopic Molecules;* Wiley: New York, 1980; pp. 95–102.
[38] The combination of B3LYP and a Pople basis set in gas phase has been widely used in the calculation of kinetic isotope effects. For examples see: (a) B. R. Beno, K. N. Houk, D. A. Singleton, *J. Am .Chem. Soc.* 1996, **118**, 9984–9985. (b) X. Zhang, A. Datta, D. A. Hrovat, W. T. Borden, *J.Am. Chem. Soc.* 2009, **131**, 16002-16003. (c) M. Huang, G. E. Garret, N. Birlirakis, L. Bohe, D. A. Pratt, D. Crich, *Nat. Chem.* 2012, **4**, 663–667.
[39] A. P. Scott, L. Radom, *J. Phys. Chem.* 1996, **100**, 16502–16513.
